# Supplementary material for: Identification of an early subset of cerebellar nuclei neurons in mice
Source: eLife. 2024 Dec 16;13:RP93778. doi: 10.7554/eLife.93778 (PMC11649241; doi:10.7554/eLife.93778)
Supplement: Figure 3—source data 1. [file elife-93778-fig3-data1.pdf]

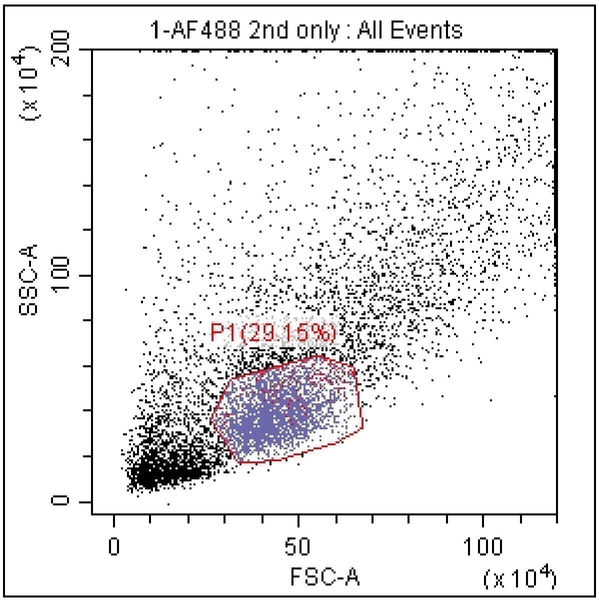

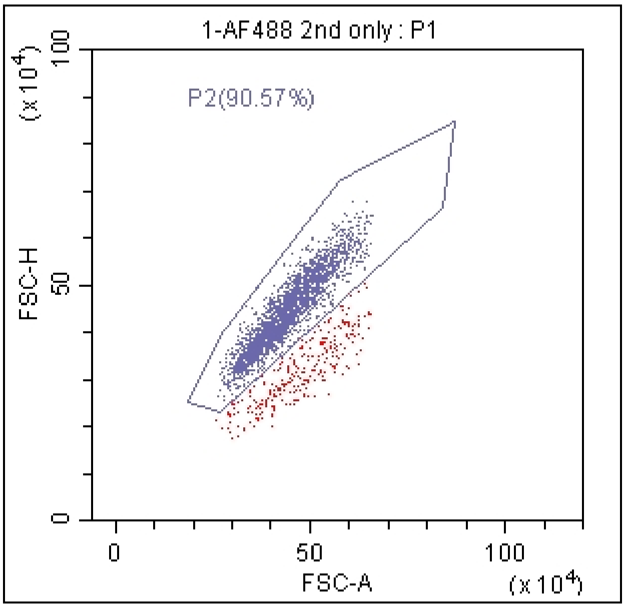

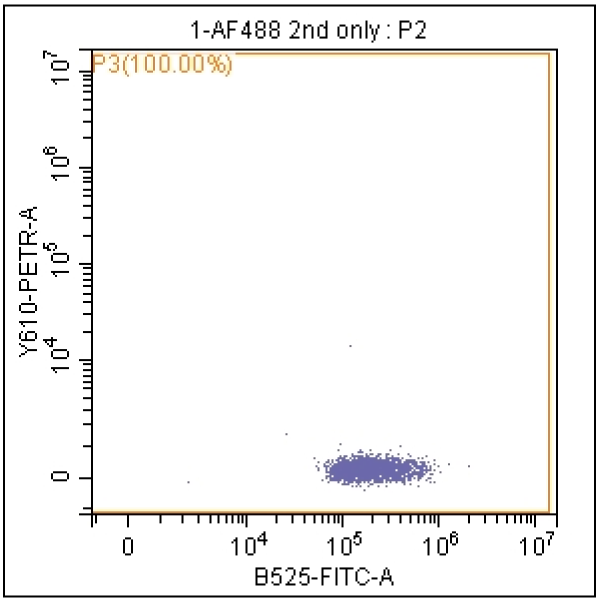

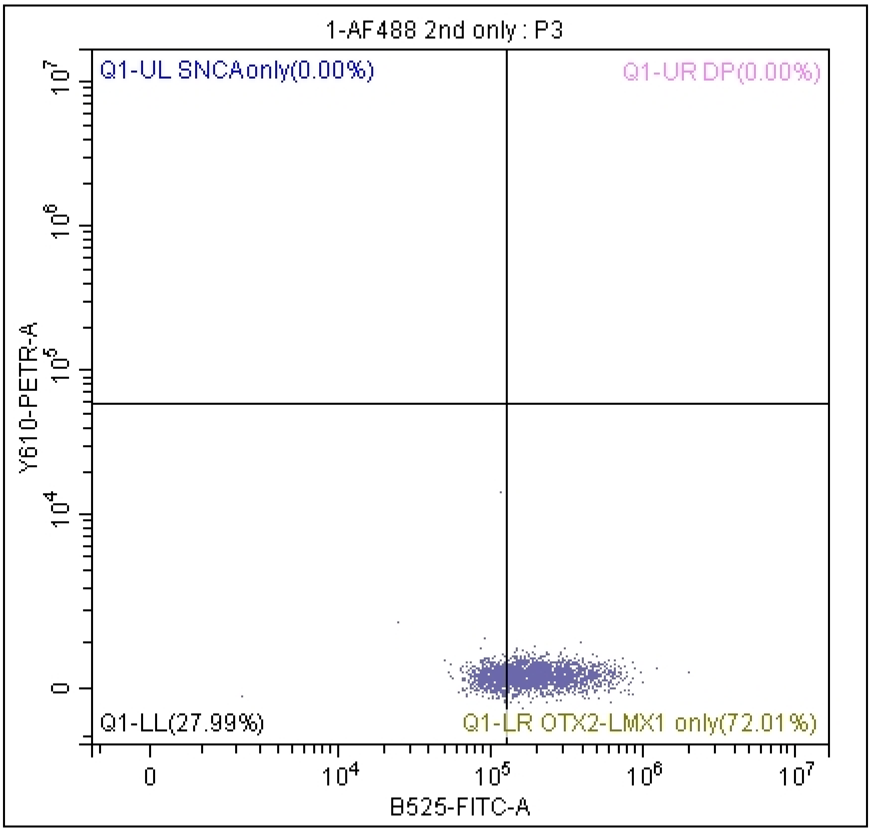

Tube Name: 1-AF488 2nd only

Sample ID:

Population	Events	% Total	% Parent
All Events	10000	100.00%	100.00%
P1	2915	29.15%	29.15%
P2	2640	26.40%	90.57%
P3	2640	26.40%	100.00%
Q1-UR DP	0	0.00%	0.00%
Q1-UL SNCA only	0	0.00%	0.00%
Q1-LL	739	7.39%	27.99%
Q1-LR OTX2-LMX1 only	1901	19.01%	72.01%

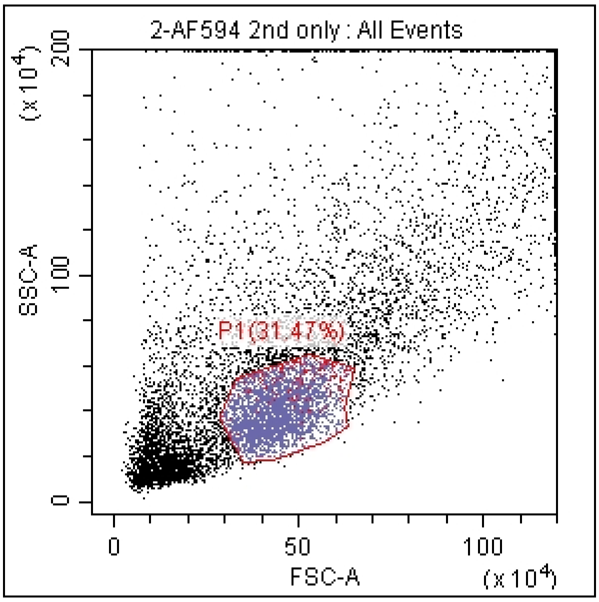

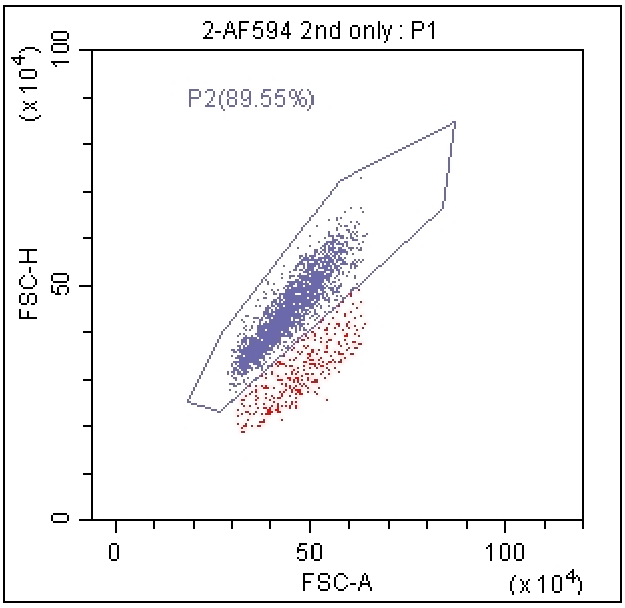

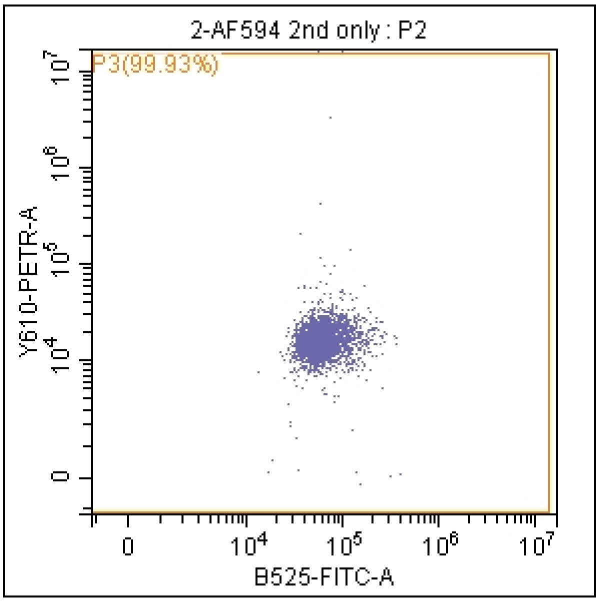

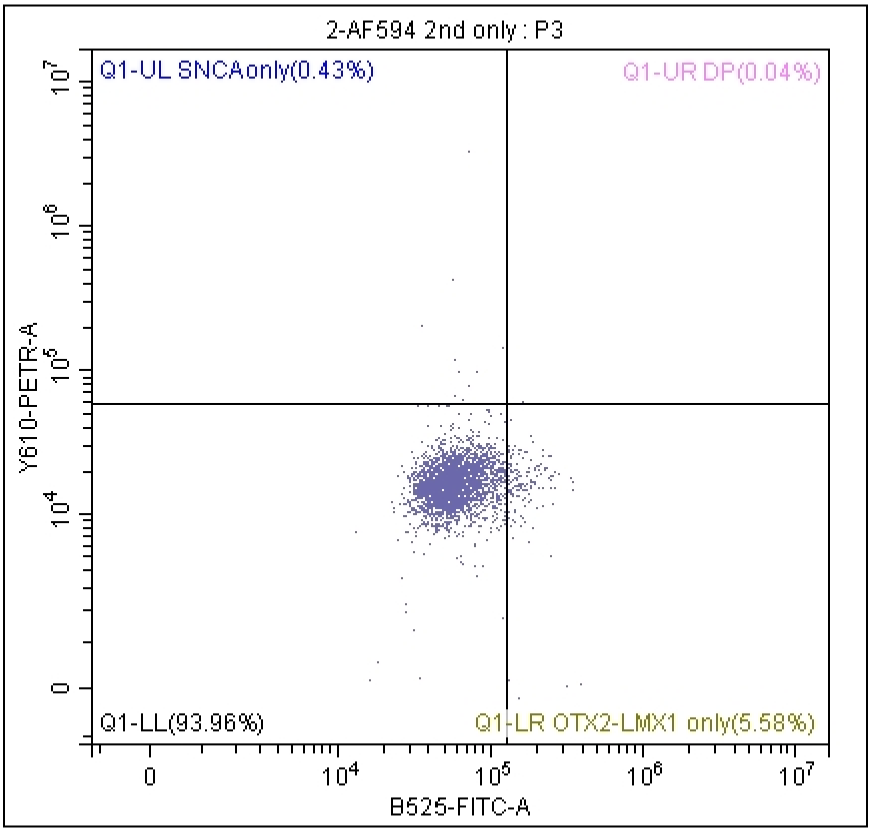

Tube Name: 2-AF594 2nd only
Sample ID:

Population	Events	% Total	% Parent
All Events	10000	100.00%	100.00%
P1	3147	31.47%	31.47%
P2	2818	28.18%	89.55%
P3	2816	28.16%	99.93%
Q1-UR DP	1	0.01%	0.04%
Q1-UL SNCA only	12	0.12%	0.43%
Q1-LL	2646	26.46%	93.96%
Q1-LR OTX2-LMX1 only	157	1.57%	5.58%

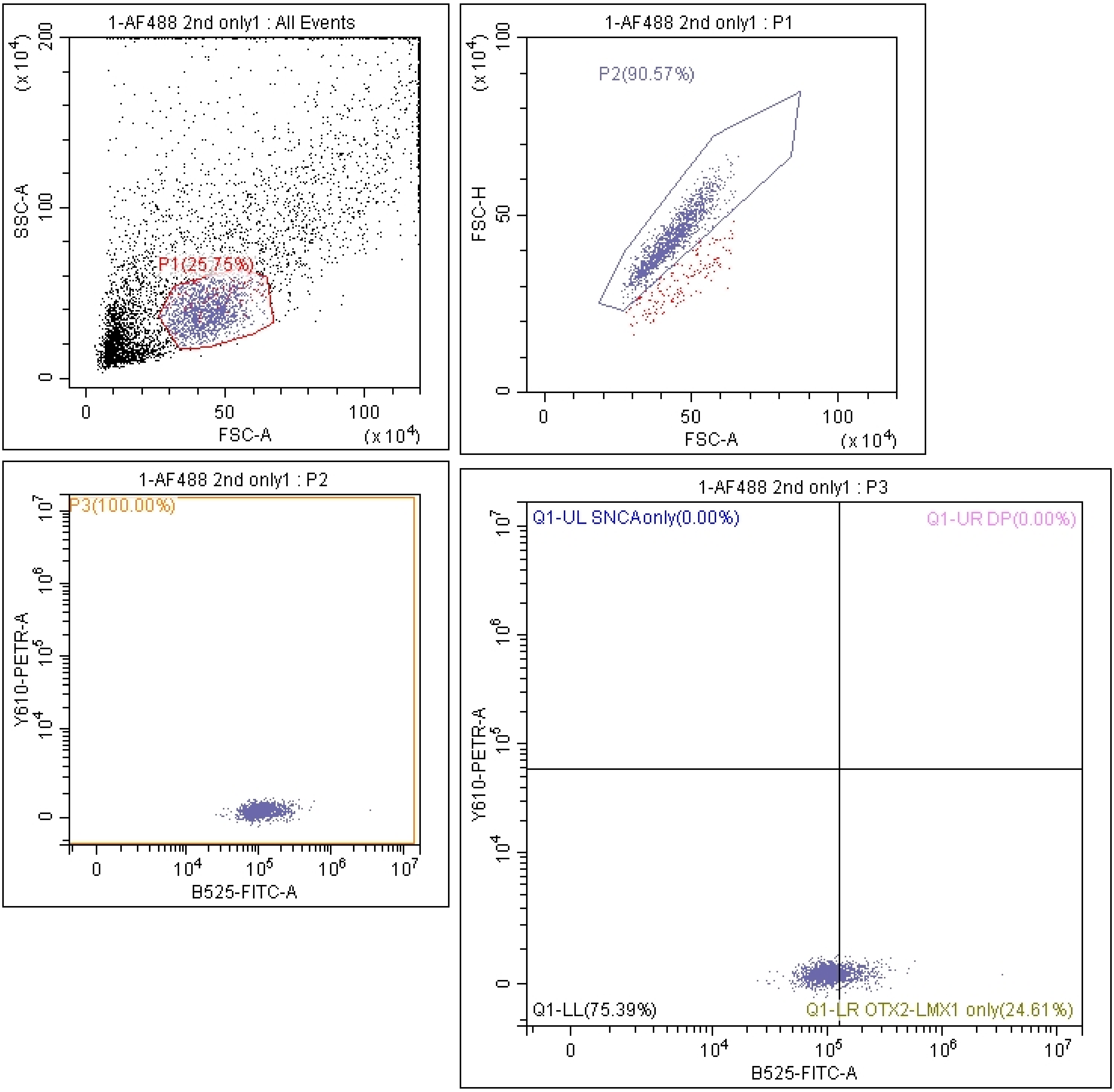

Tube Name: 1-AF488 2nd only1

Sample ID:

Population	Events	% Total	% Parent
All Events	6342	100.00%	100.00%
P1	1633	25.75%	25.75%
P2	1479	23.32%	90.57%
P3	1479	23.32%	100.00%
Q1-UR DP	0	0.00%	0.00%
Q1-UL SNCA only	0	0.00%	0.00%
Q1-LL	1115	17.58%	75.39%
Q1-LR OTX2-LMX1 only	364	5.74%	24.61%

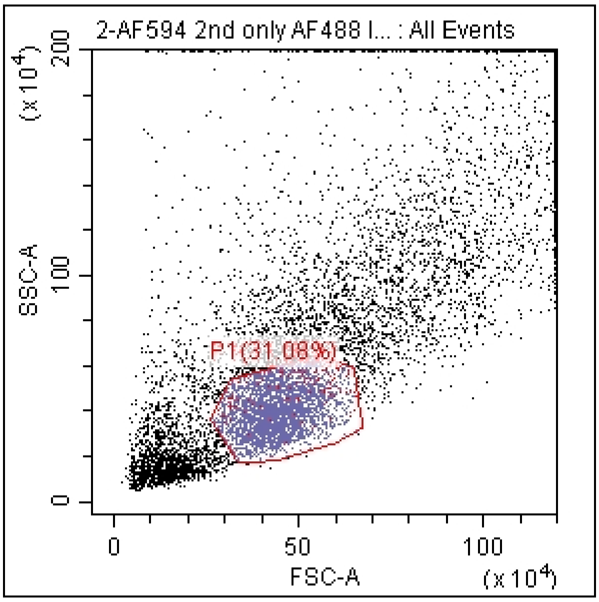

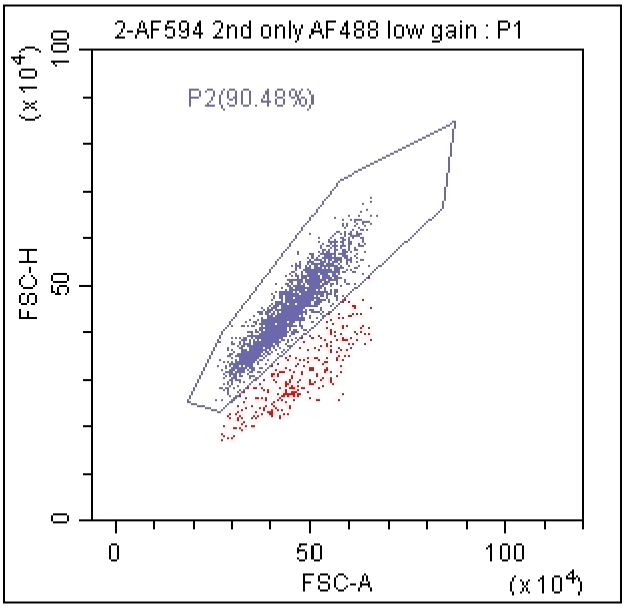

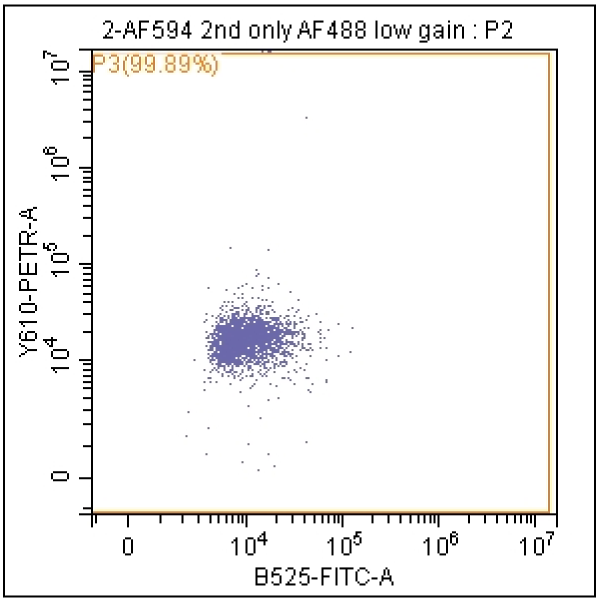

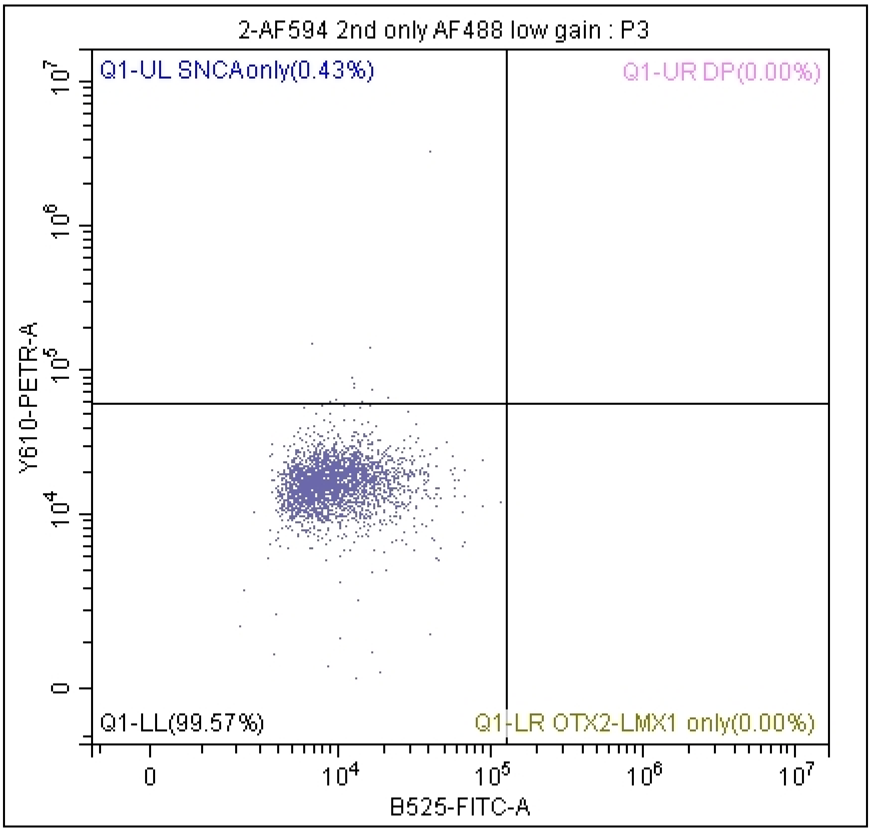

Tube Name: 2-AF594 2nd only AF488 low gain

Sample ID:

Population	Events	% Total	% Parent
All Events	10000	100.00%	100.00%
P1	3108	31.08%	31.08%
P2	2812	28.12%	90.48%
P3	2809	28.09%	99.89%
Q1-UR DP	0	0.00%	0.00%
Q1-UL SNCA only	12	0.12%	0.43%
Q1-LL	2797	27.97%	99.57%
Q1-LR OTX2-LMX1 only	0	0.00%	0.00%

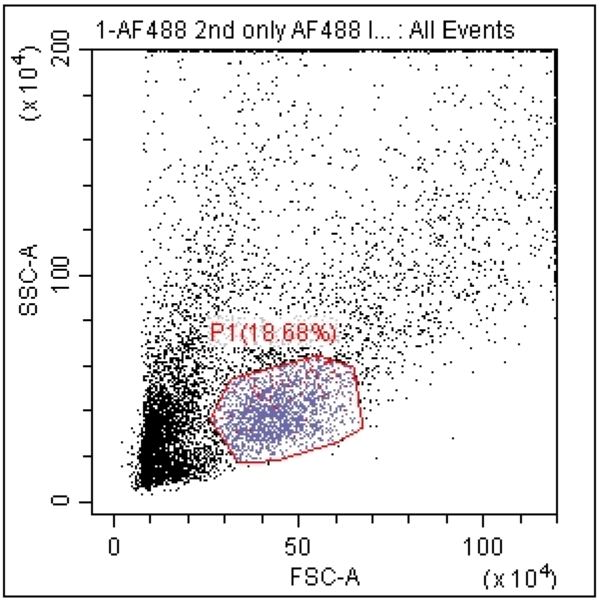

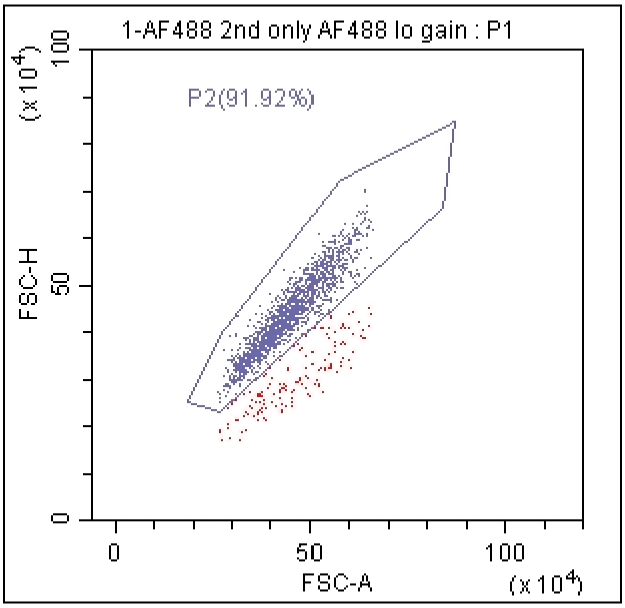

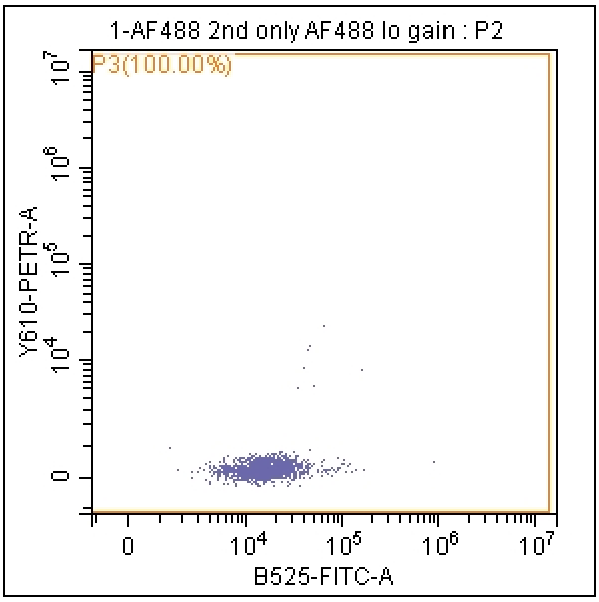

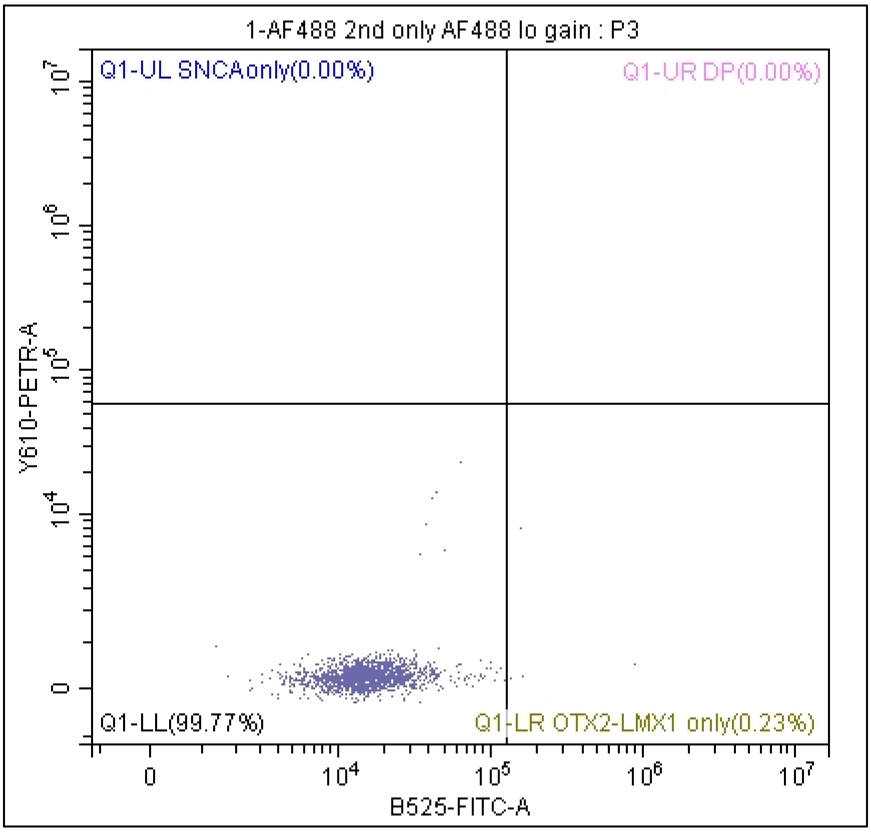

Tube Name: 1-AF488 2nd only AF488 lo gain

Sample ID:

Population	Events	% Total	% Parent
All Events	10000	100.00%	100.00%
P1	1868	18.68%	18.68%
P2	1717	17.17%	91.92%
P3	1717	17.17%	100.00%
Q1-UR DP	0	0.00%	0.00%
Q1-UL SNCA only	0	0.00%	0.00%
Q1-LL	1713	17.13%	99.77%
Q1-LR OTX2-LMX1 only	4	0.04%	0.23%

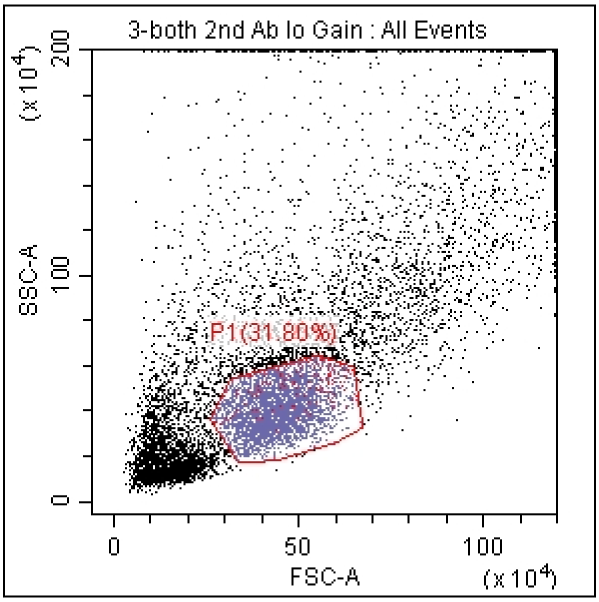

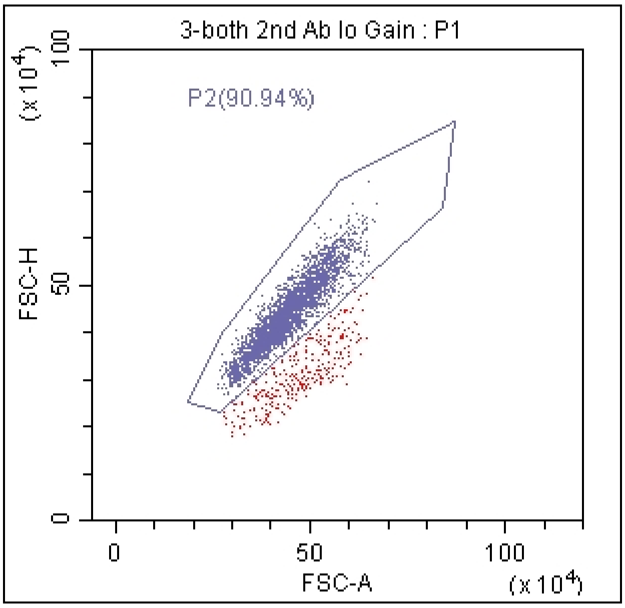

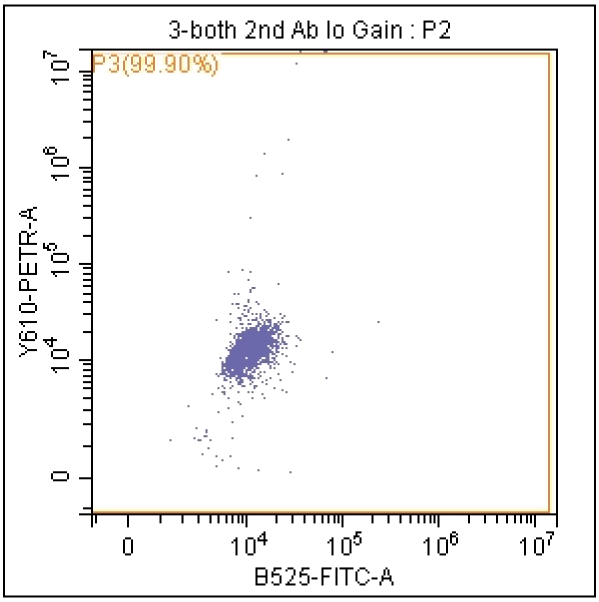

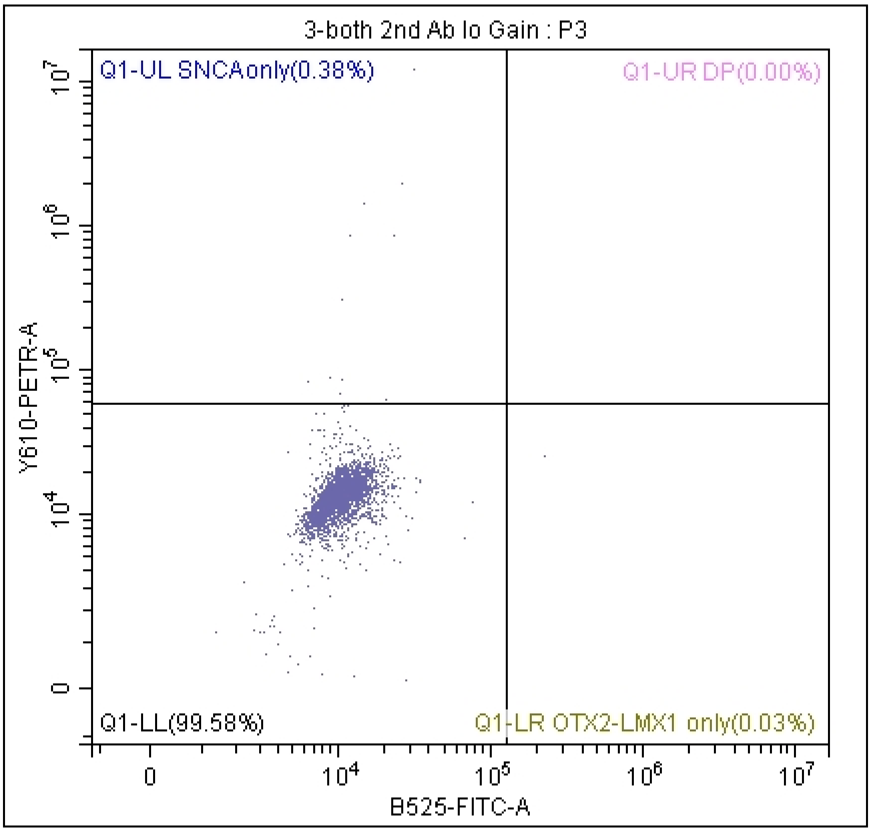

Tube Name: 3-both 2nd Ab lo Gain

Sample ID:

Population	Events	% Total	% Parent
All Events	10000	100.00%	100.00%
P1	3180	31.80%	31.80%
P2	2892	28.92%	90.94%
P3	2889	28.89%	99.90%
Q1-UR DP	0	0.00%	0.00%
Q1-UL SNCA only	11	0.11%	0.38%
Q1-LL	2877	28.77%	99.58%
Q1-LR OTX2-LMX1 only	1	0.01%	0.03%

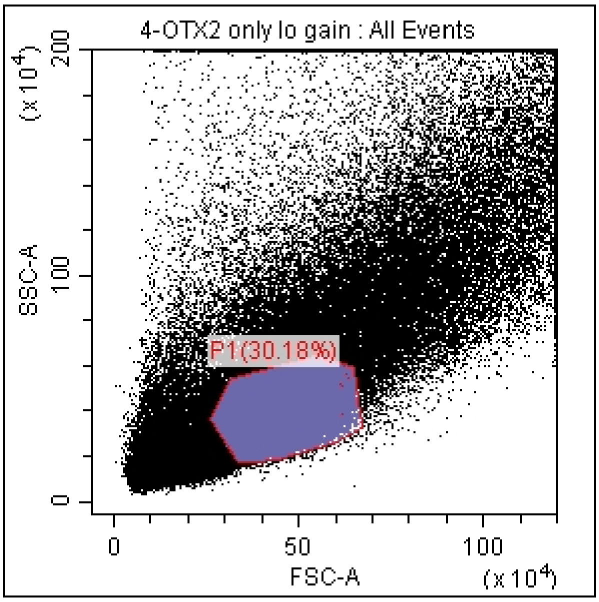

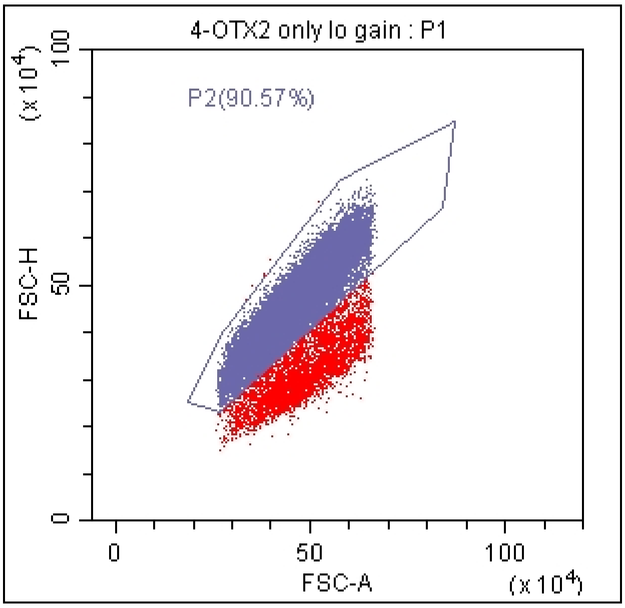

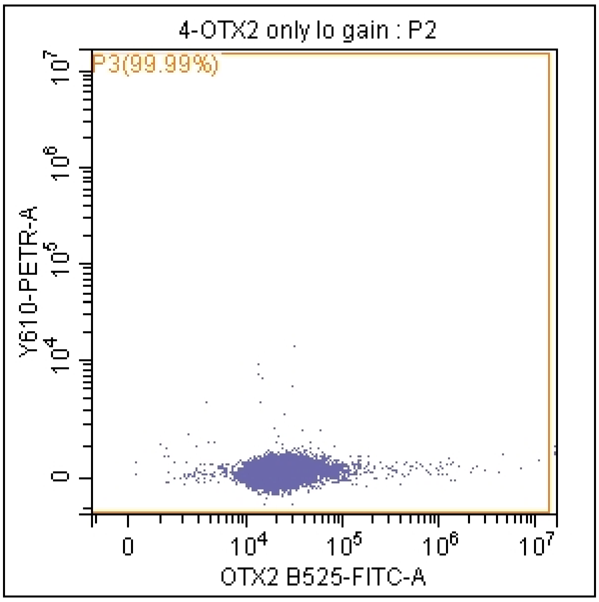

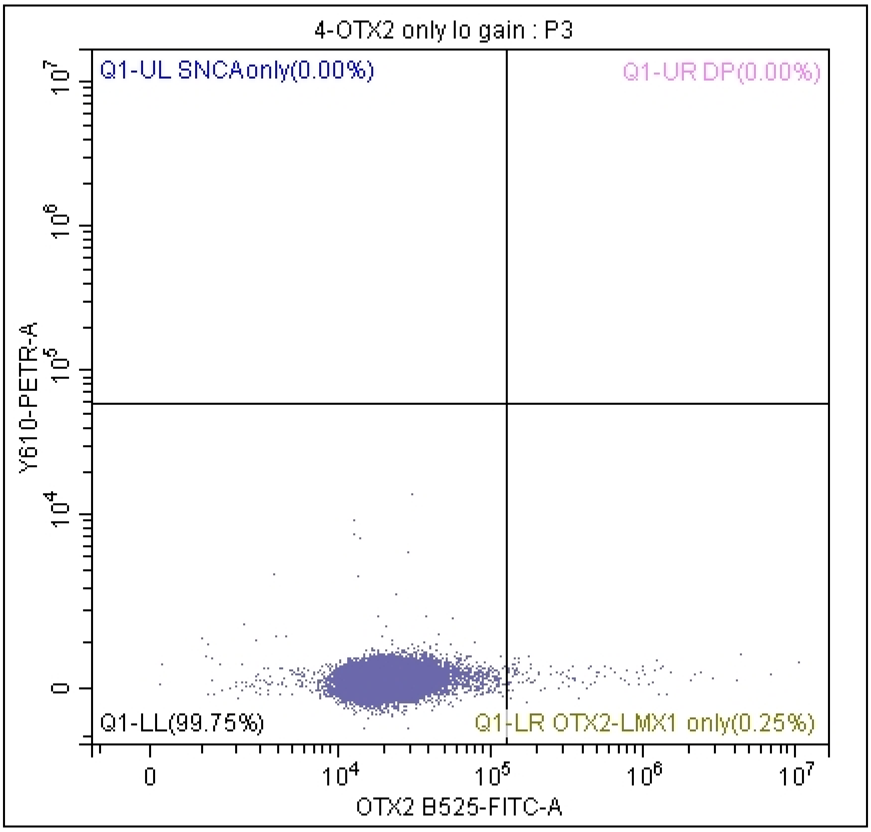

Tube Name: 4-OTX2 only lo gain

Sample ID:

Population	Events	% Total	% Parent
All Events	150000	100.00%	100.00%
P1	45267	30.18%	30.18%
P2	40999	27.33%	90.57%
P3	40995	27.33%	99.99%
Q1-UR DP	0	0.00%	0.00%
Q1-UL SNCA only	0	0.00%	0.00%
Q1-LL	40893	27.26%	99.75%
Q1-LR OTX2-LMX1 only	102	0.07%	0.25%

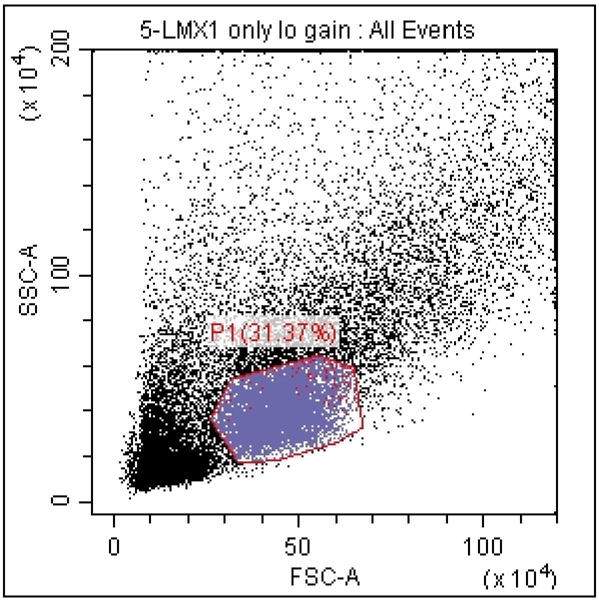

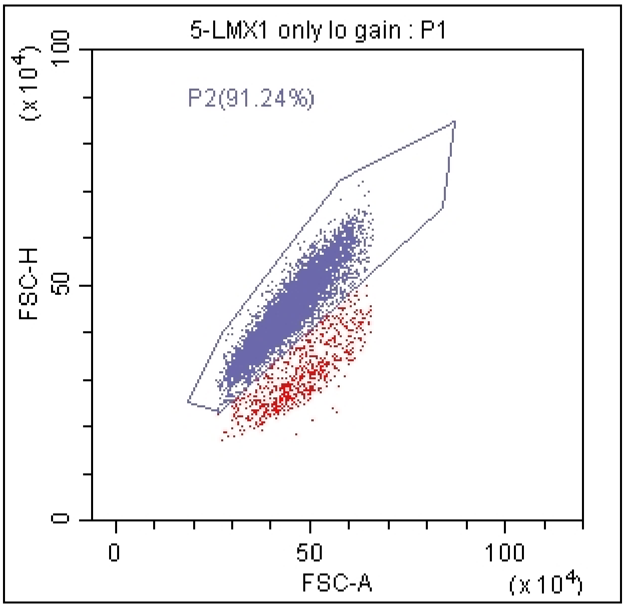

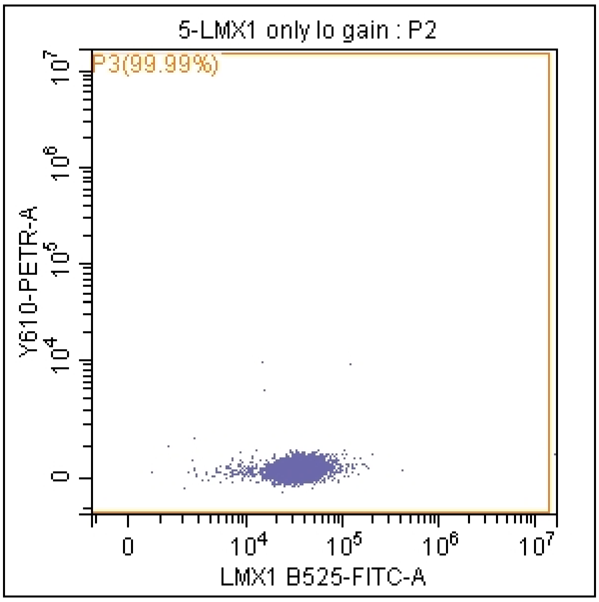

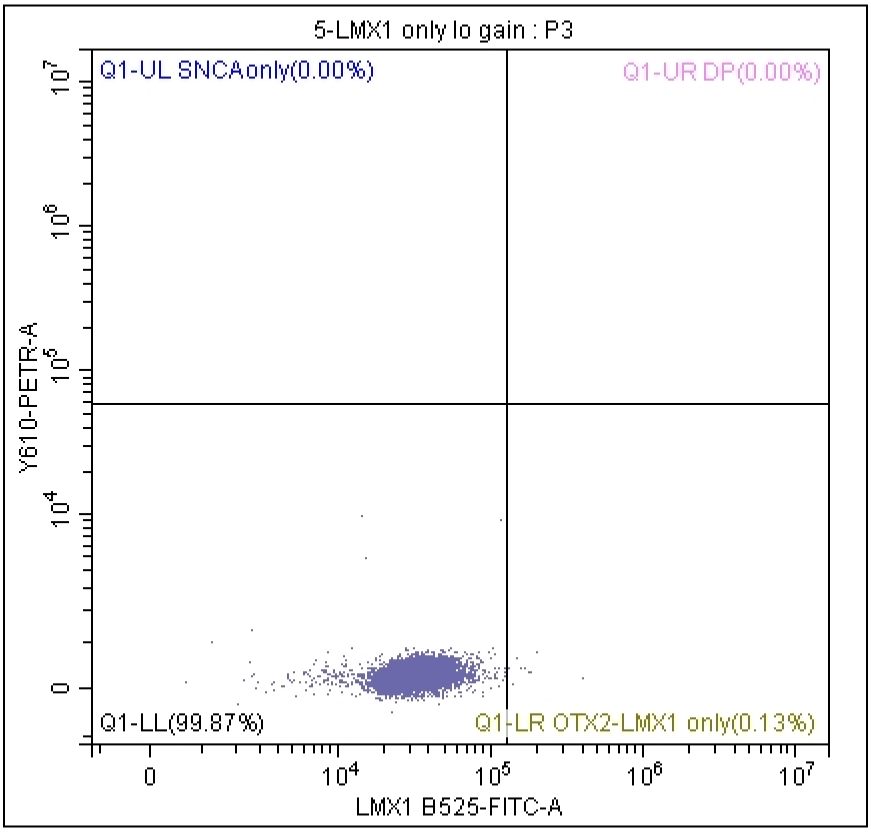

Tube Name: 5-LMX1 only lo gain

Sample ID:

Population	Events	% Total	% Parent
All Events	24918	100.00%	100.00%
P1	7816	31.37%	31.37%
P2	7131	28.62%	91.24%
P3	7130	28.61%	99.99%
Q1-UR DP	0	0.00%	0.00%
Q1-UL SNCA only	0	0.00%	0.00%
Q1-LL	7121	28.58%	99.87%
Q1-LR OTX2-LMX1 only	9	0.04%	0.13%

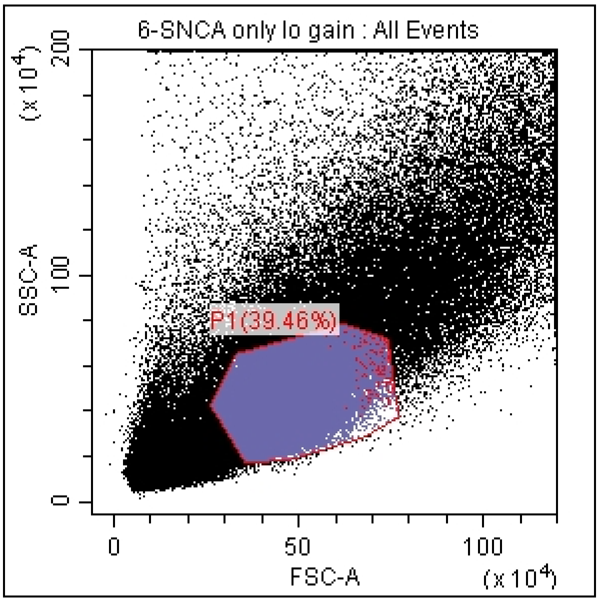

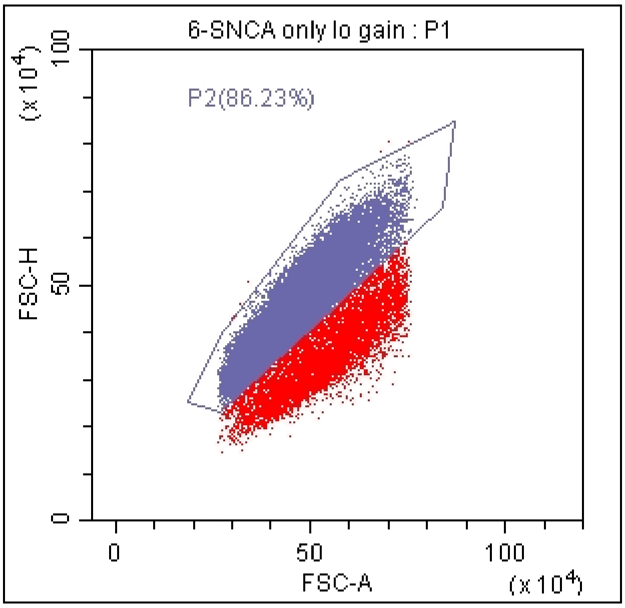

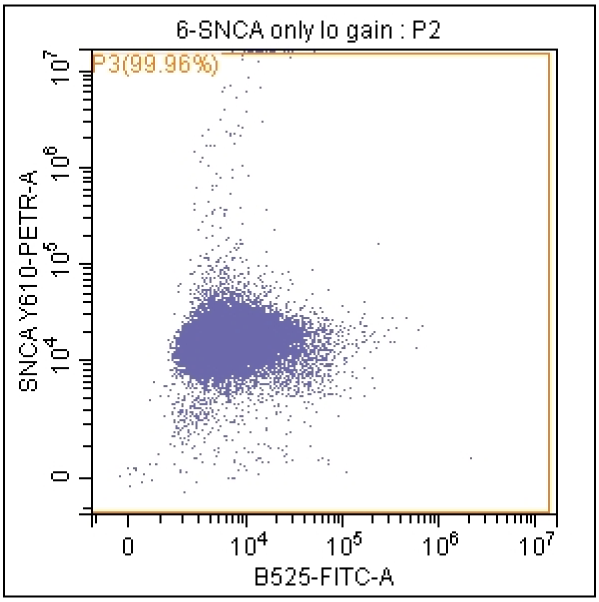

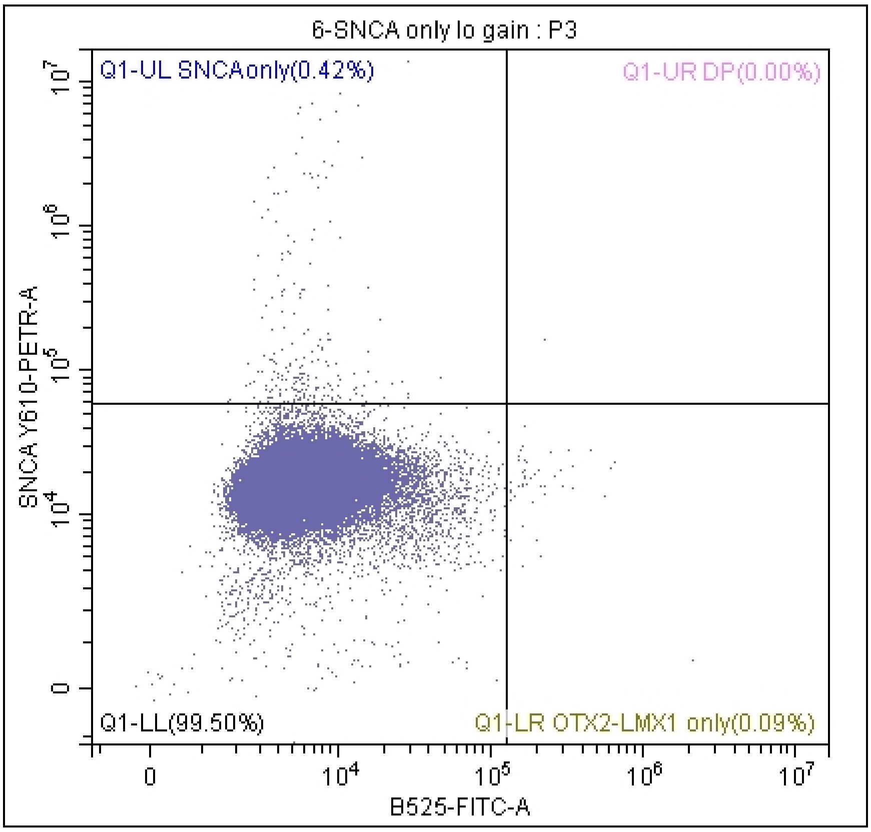

Tube Name: 6-SNCA only lo gain

Sample ID:

Population	Events	% Total	% Parent
All Events	150000	100.00%	100.00%
P1	59192	39.46%	39.46%
P2	51044	34.03%	86.23%
P3	51024	34.02%	99.96%
Q1-UR DP	1	0.00%	0.00%
Q1-UL SNCA only	212	0.14%	0.42%
Q1-LL	50767	33.84%	99.50%
Q1-LR OTX2-LMX1 only	44	0.03%	0.09%

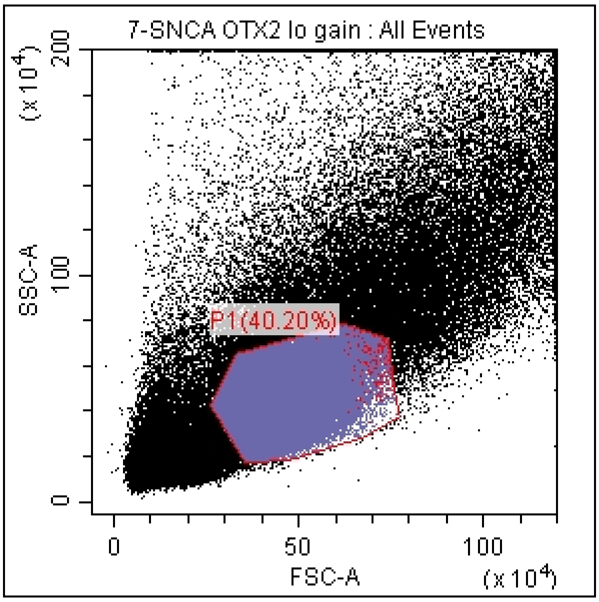

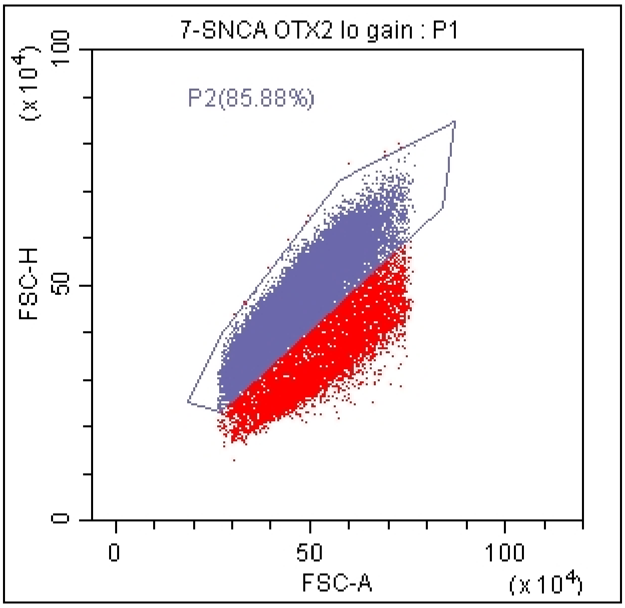

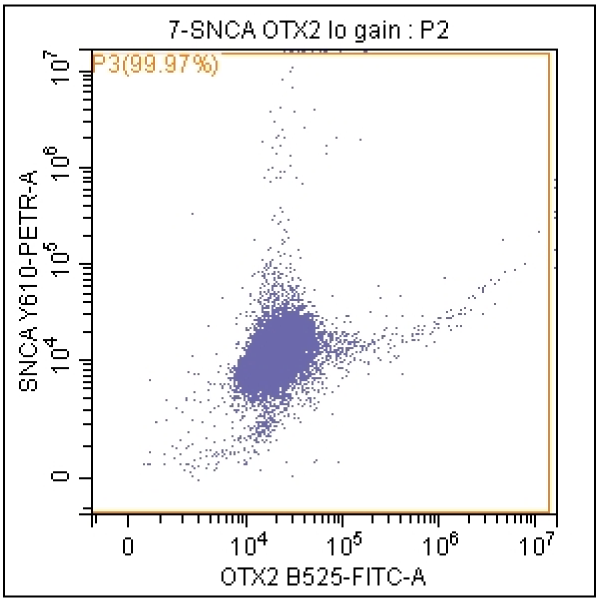

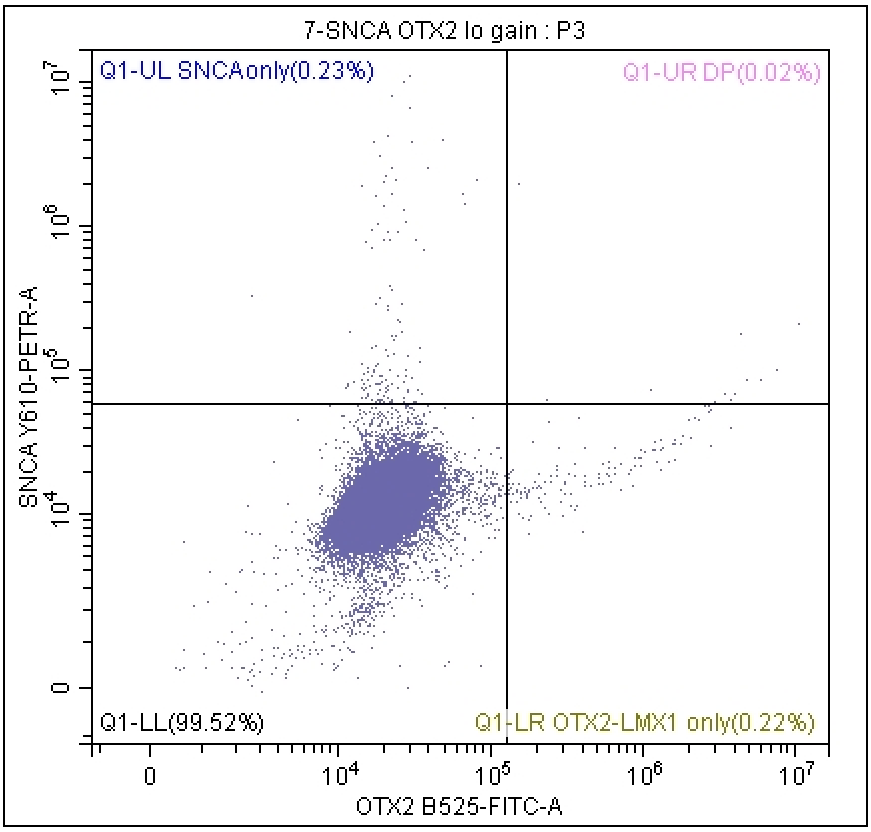

Tube Name: 7-SNCA OTX2 lo gain

Sample ID:

Population	Events	% Total	% Parent
All Events	158072	100.00%	100.00%
P1	63546	40.20%	40.20%
P2	54571	34.52%	85.88%
P3	54552	34.51%	99.97%
Q1-UR DP	12	0.01%	0.02%
Q1-UL SNCA only	128	0.08%	0.23%
Q1-LL	54290	34.35%	99.52%
Q1-LR OTX2-LMX1 only	122	0.08%	0.22%

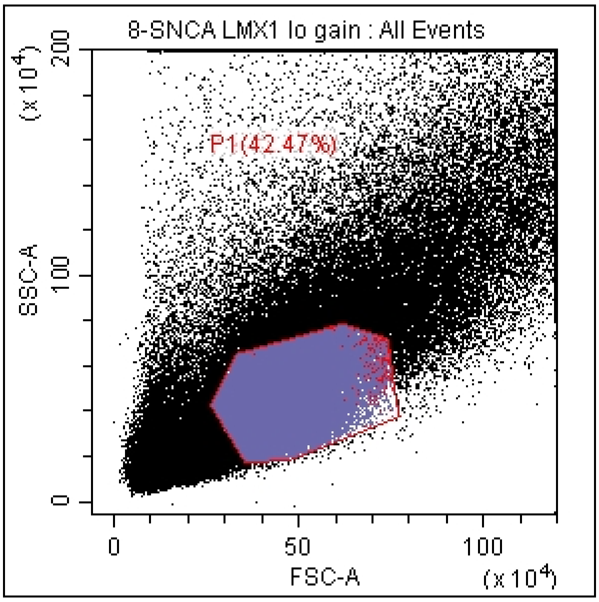

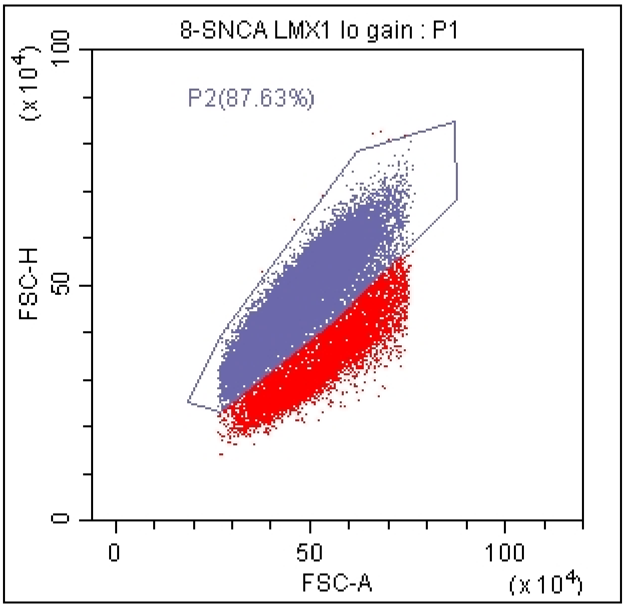

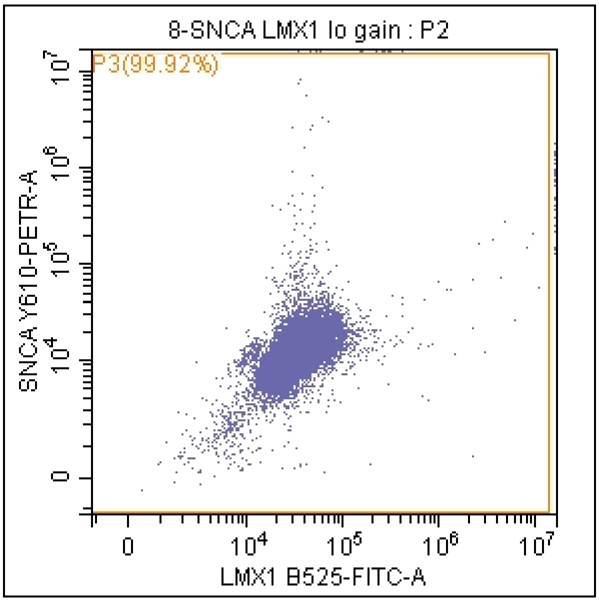

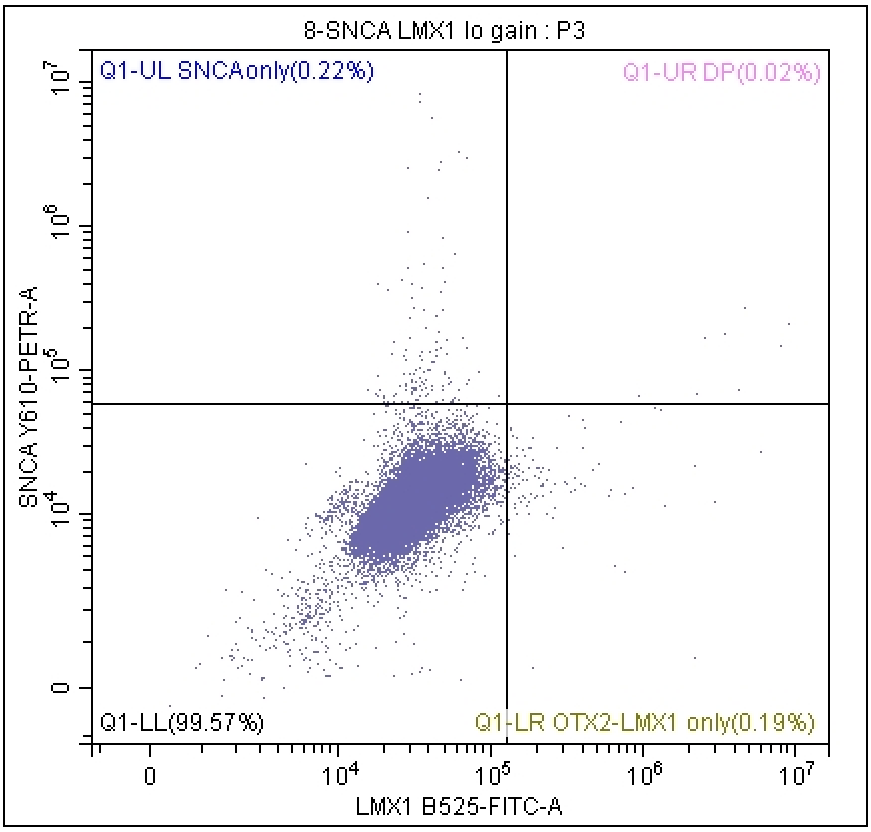

Tube Name: 8-SNCA LMX1 lo gain

Sample ID:

Population	Events	% Total	% Parent
All Events	150000	100.00%	100.00%
P1	63704	42.47%	42.47%
P2	55823	37.22%	87.63%
P3	55781	37.19%	99.92%
Q1-UR DP	9	0.01%	0.02%
Q1-UL SNCA only	125	0.08%	0.22%
Q1-LL	55539	37.03%	99.57%
Q1-LR OTX2-LMX1 only	108	0.07%	0.19%
