# Supplementary material for: Identification of an early subset of cerebellar nuclei neurons in mice
Source: eLife. 2024 Dec 16;13:RP93778. doi: 10.7554/eLife.93778 (PMC11649241; doi:10.7554/eLife.93778)
Supplement: Figure 3—source data 2. [file elife-93778-fig3-data2.zip › 190903 Maryam Celebellum flow.pdf]

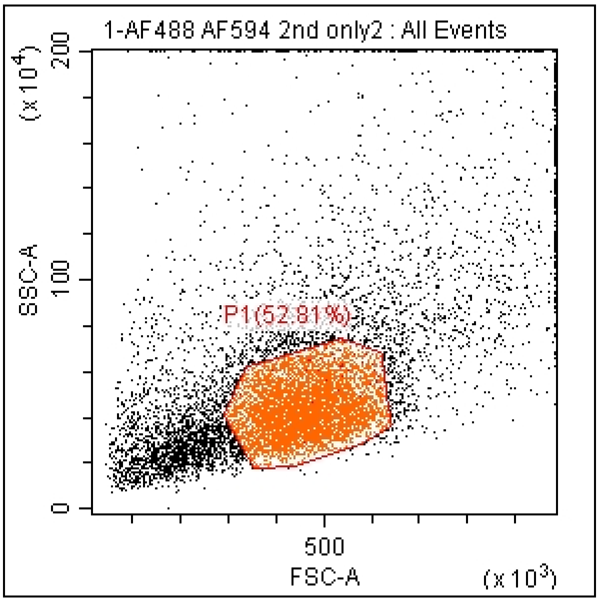

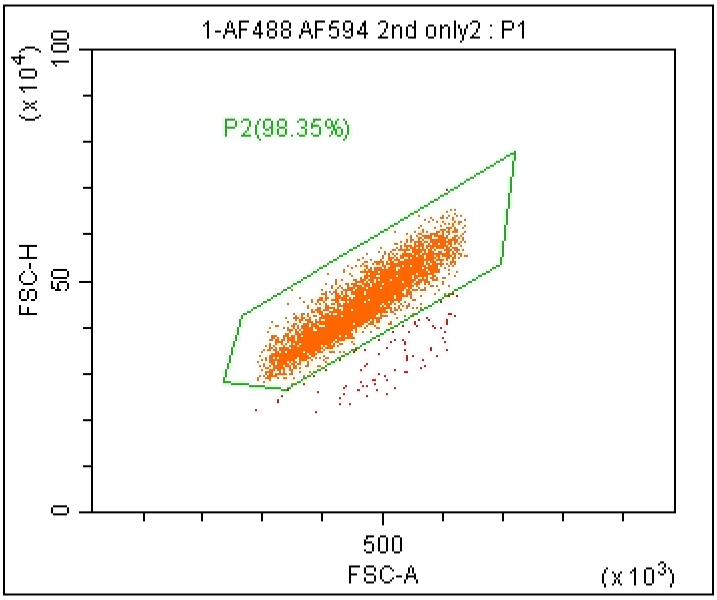

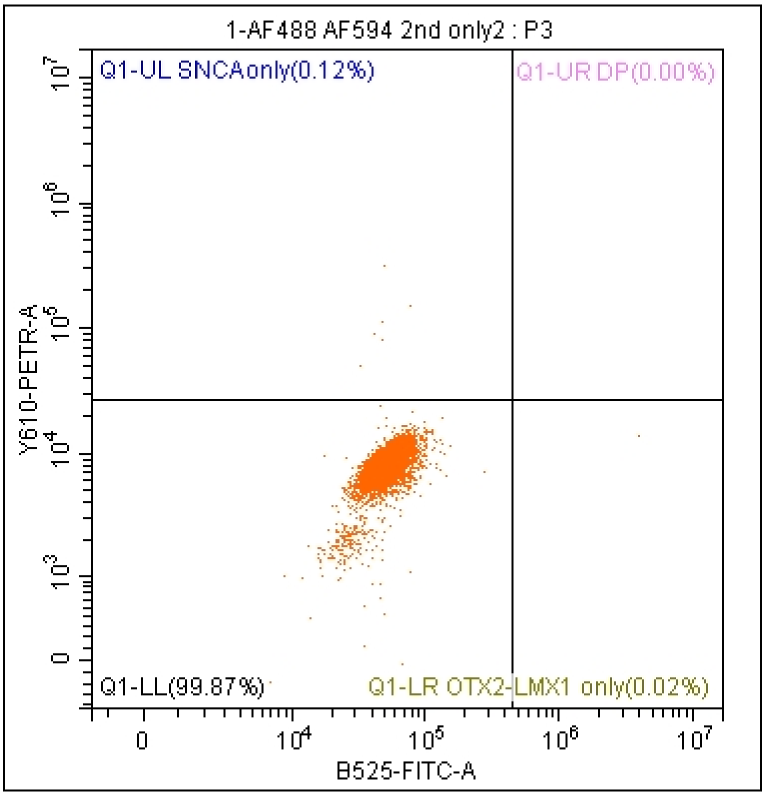

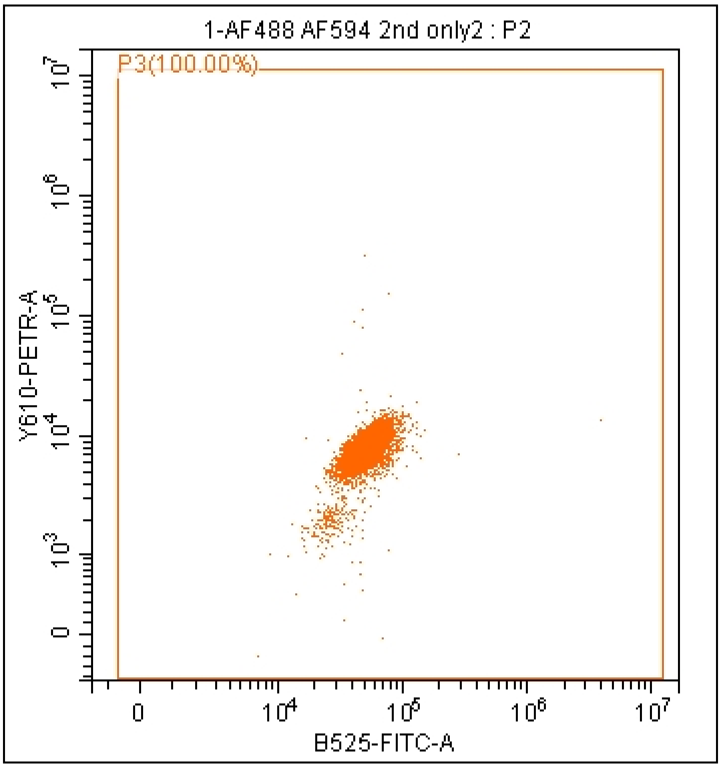

Tube Name: 1-AF488 AF594 2nd only2

Sample ID:

Population	Events	% Total	% Parent
All Events	10000	100.00%	100.00%
P1	5281	52.81%	52.81%
P2	5194	51.94%	98.35%
P3	5194	51.94%	100.00%
Q1-UR DP	0	0.00%	0.00%
Q1-UL SNCA only	6	0.06%	0.12%
Q1-LL	5187	51.87%	99.87%
Q1-LR OTX2-LMX1 only	1	0.01%	0.02%

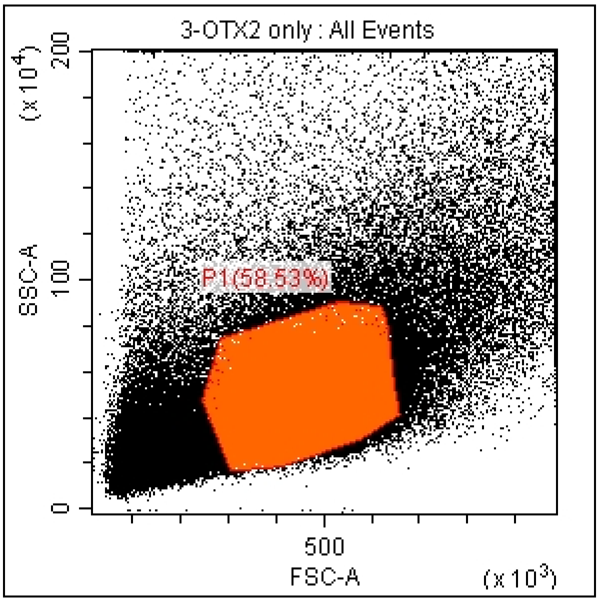

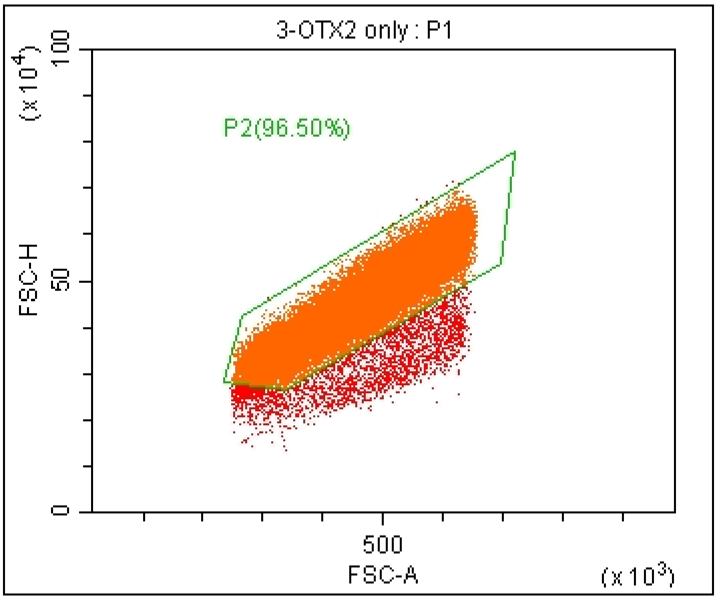

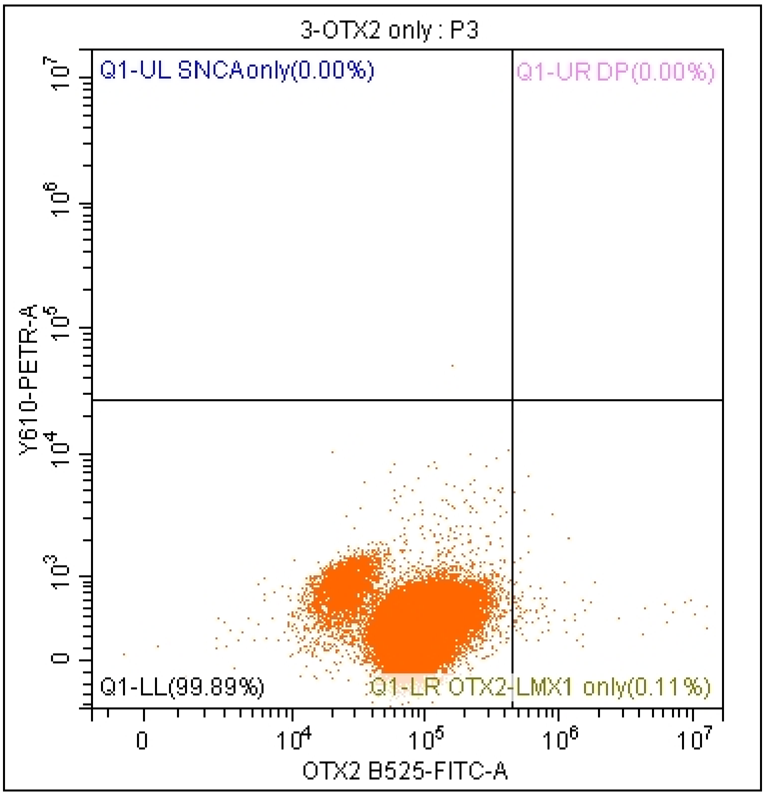

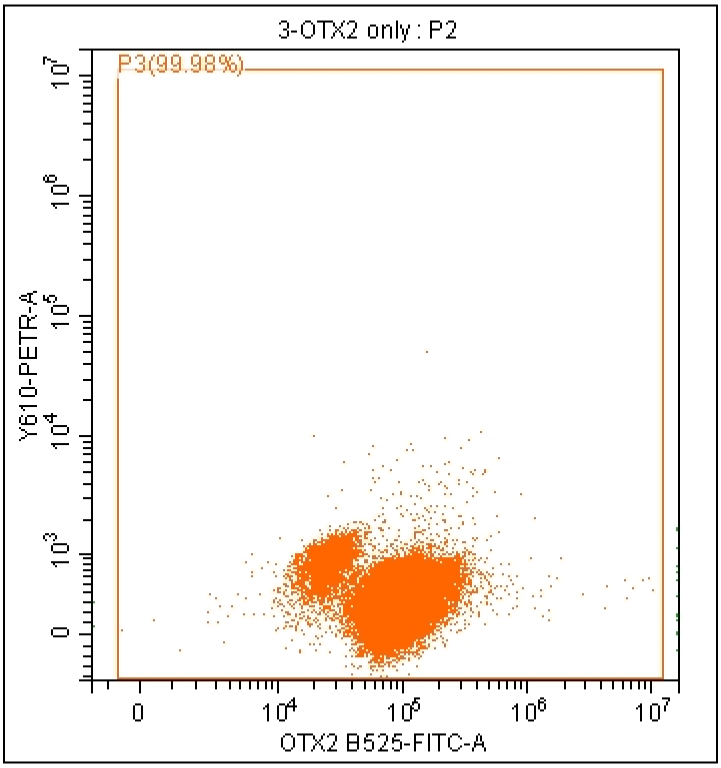

Tube Name: 3-OTX2 only

Sample ID:

Population	Events	% Total	% Parent
All Events	140000	100.00%	100.00%
P1	81935	58.53%	58.53%
P2	79070	56.48%	96.50%
P3	79055	56.47%	99.98%
Q1-UR DP	0	0.00%	0.00%
Q1-UL SNCA only	1	0.00%	0.00%
Q1-LL	78970	56.41%	99.89%
Q1-LR OTX2-LMX1 only	84	0.06%	0.11%

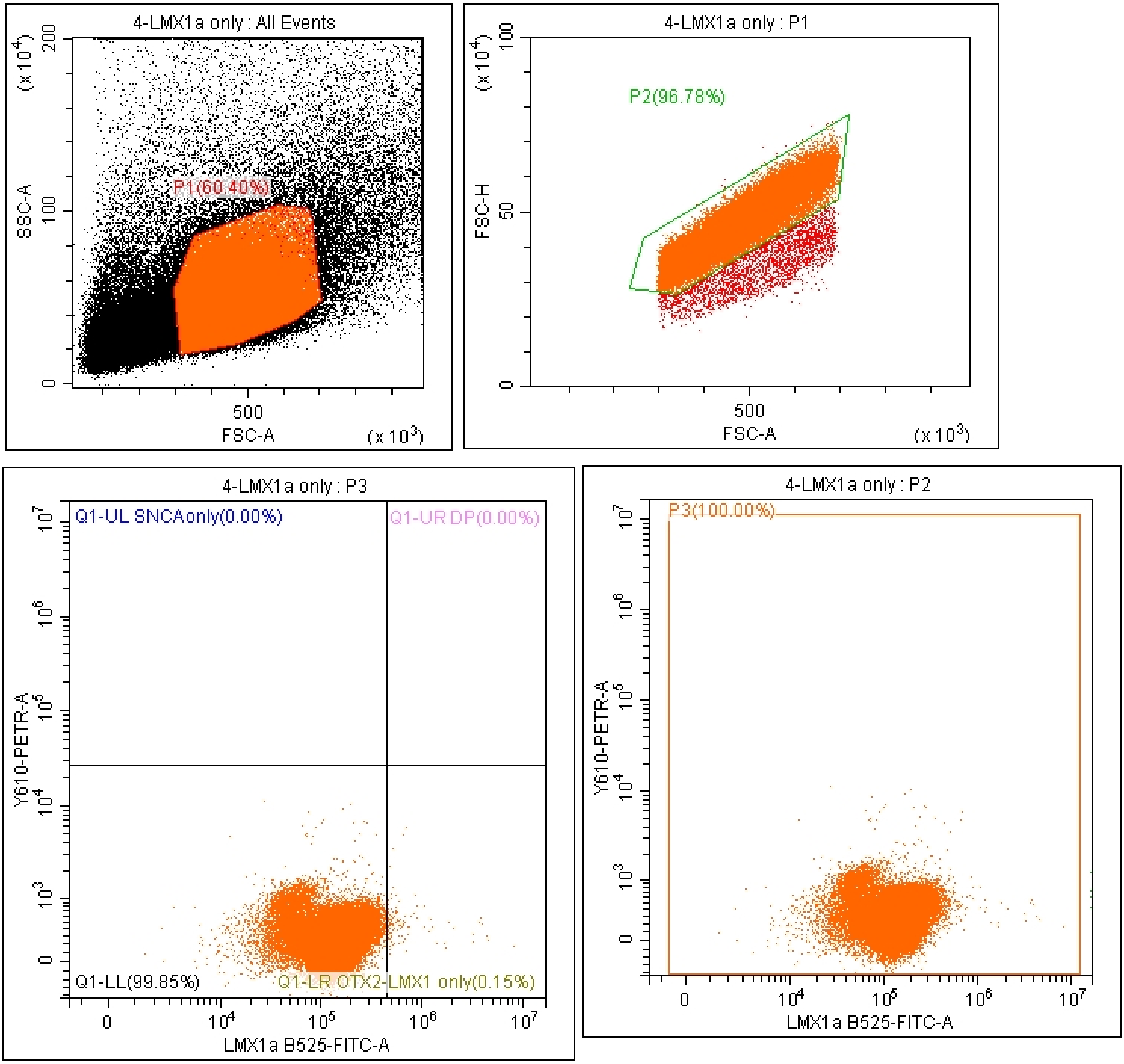

Tube Name: 4-LMX1a only
Sample ID:

Population	Events	% Total	% Parent
All Events	140000	100.00%	100.00%
P1	84557	60.40%	60.40%
P2	81836	58.45%	96.78%
P3	81832	58.45%	100.00%
Q1-UR DP	0	0.00%	0.00%
Q1-UL SNCA only	0	0.00%	0.00%
Q1-LL	81710	58.36%	99.85%
Q1-LR OTX2-LMX1 only	122	0.09%	0.15%

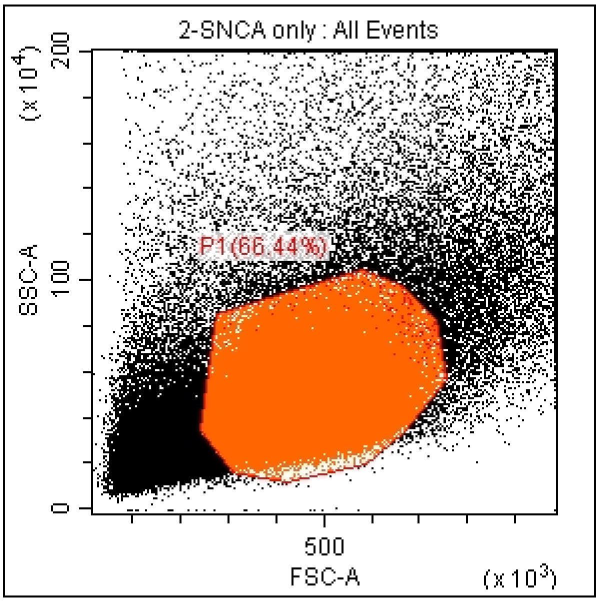

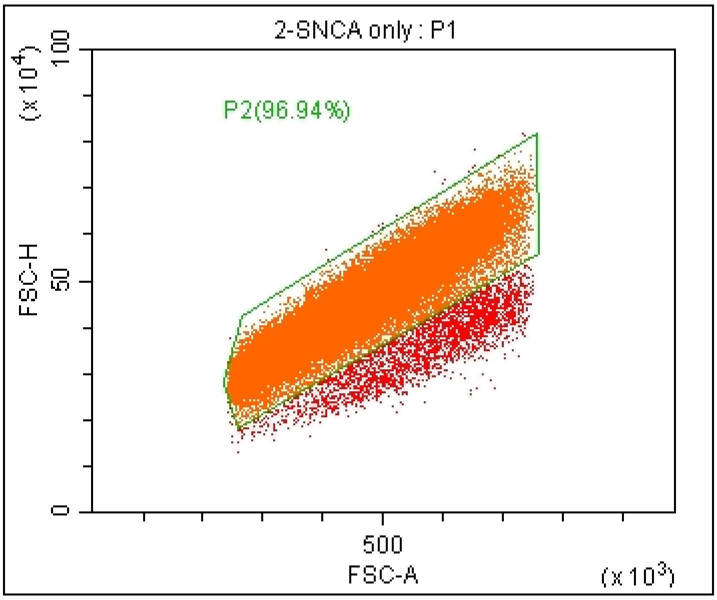

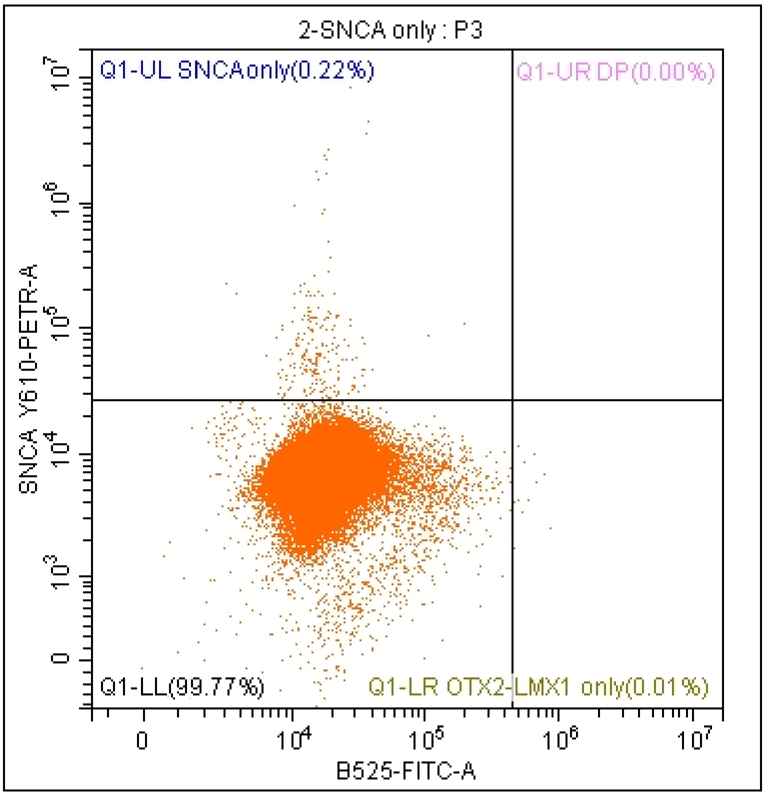

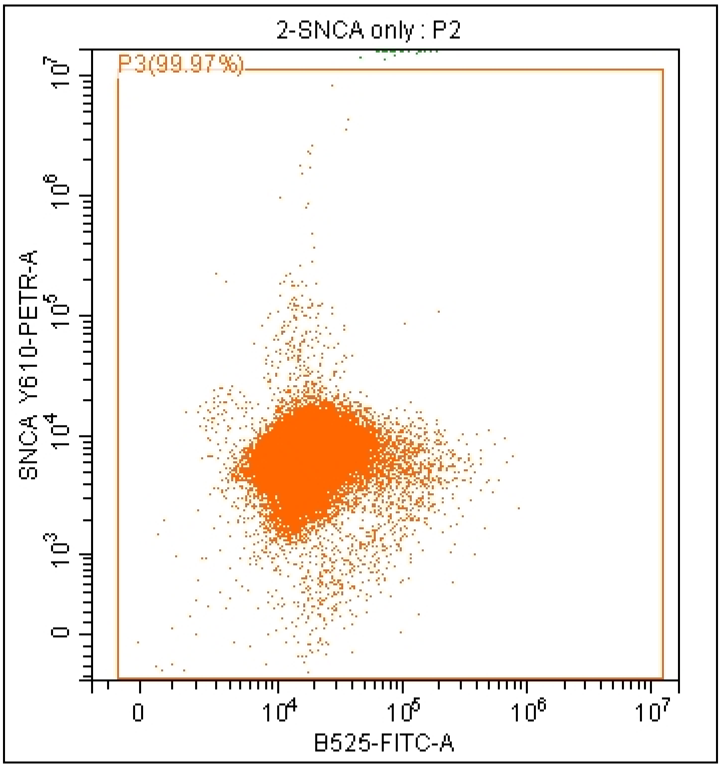

Tube Name: 2-SNCA only

Sample ID:

Population	Events	% Total	% Parent
All Events	150000	100.00%	100.00%
P1	99658	66.44%	66.44%
P2	96606	64.40%	96.94%
P3	96581	64.39%	99.97%
Q1-UR DP	0	0.00%	0.00%
Q1-UL SNCA only	214	0.14%	0.22%
Q1-LL	96356	64.24%	99.77%
Q1-LR OTX2-LMX1 only	11	0.01%	0.01%

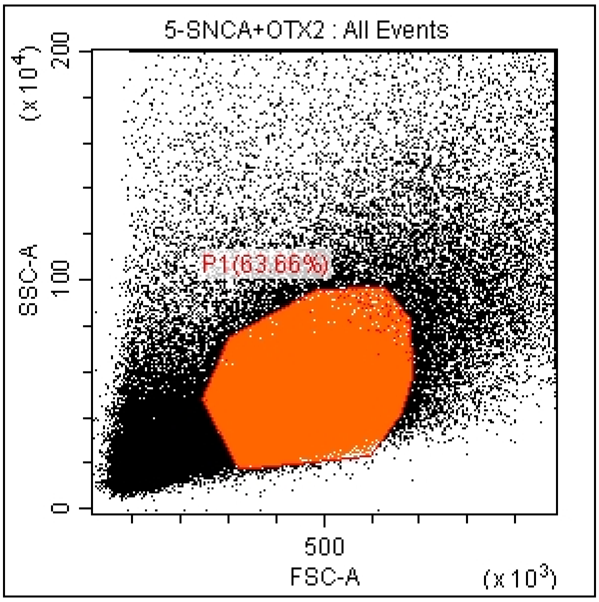

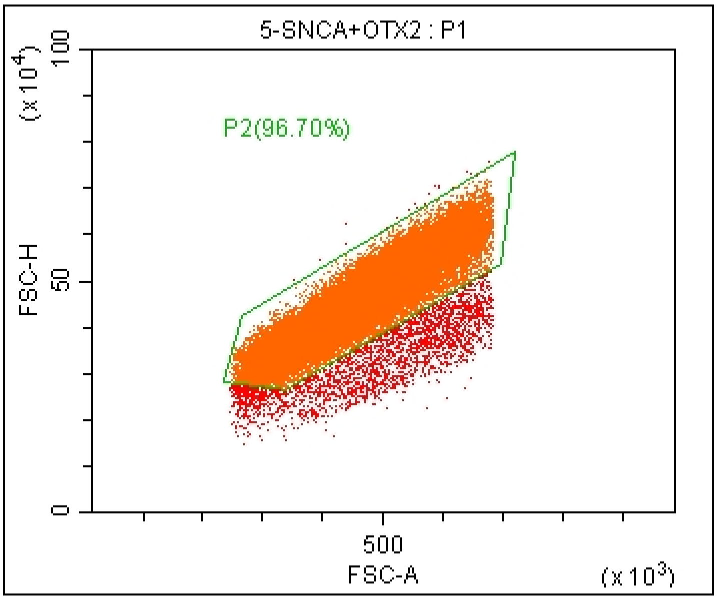

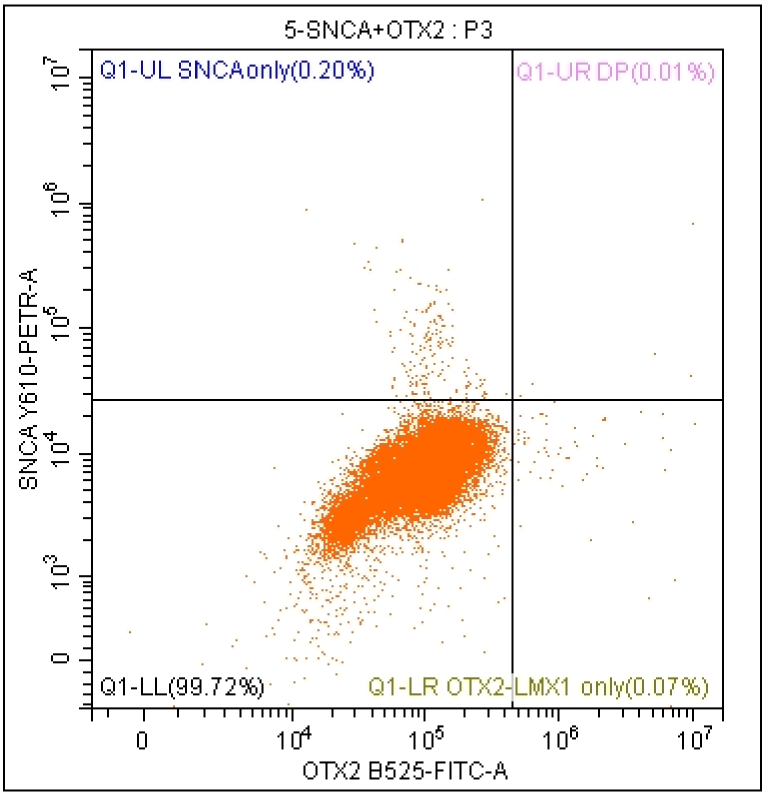

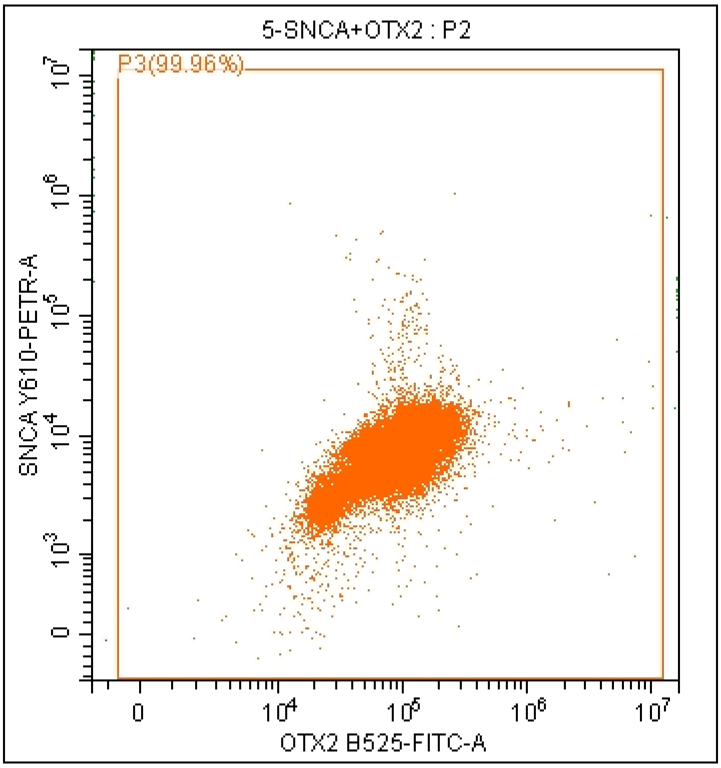

Tube Name: 5-SNCA+OTX2

Sample ID:

Population	Events	% Total	% Parent
All Events	140000	100.00%	100.00%
P1	89122	63.66%	63.66%
P2	86178	61.56%	96.70%
P3	86144	61.53%	99.96%
Q1-UR DP	5	0.00%	0.01%
Q1-UL SNCA only	176	0.13%	0.20%
Q1-LL	85900	61.36%	99.72%
Q1-LR OTX2-LMX1 only	63	0.05%	0.07%

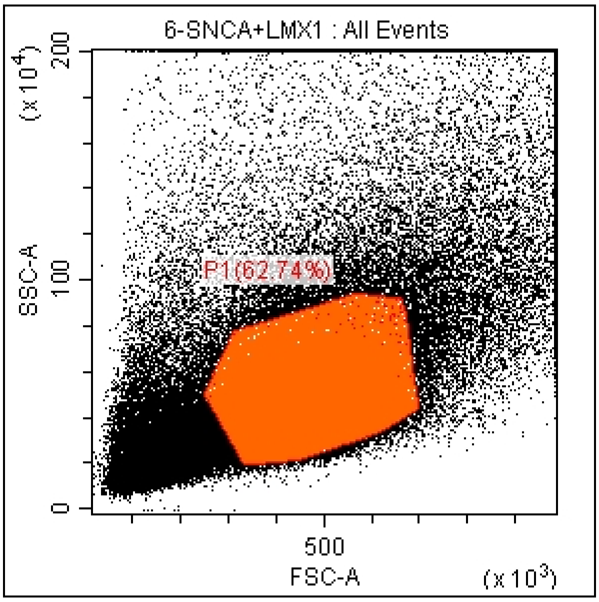

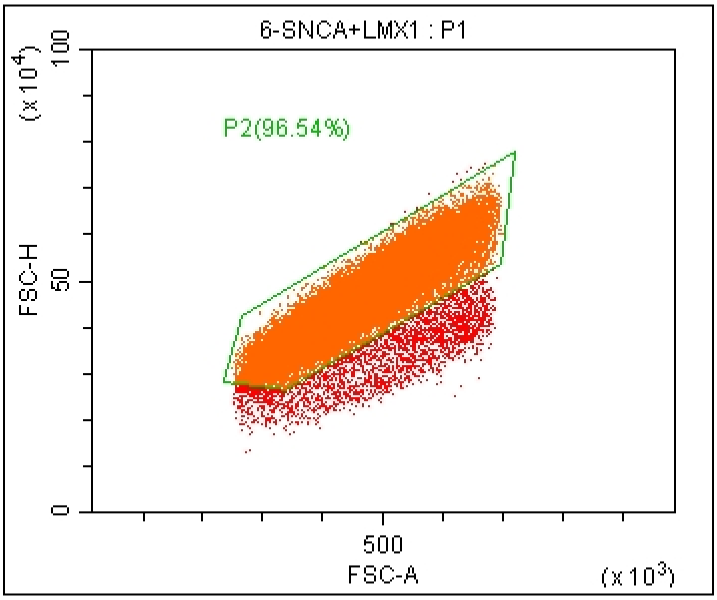

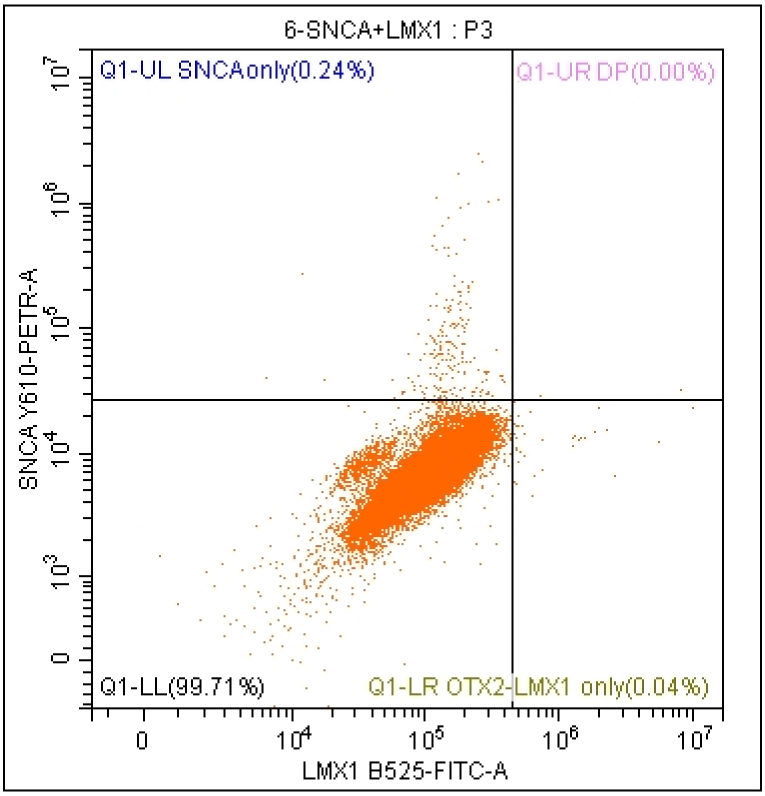

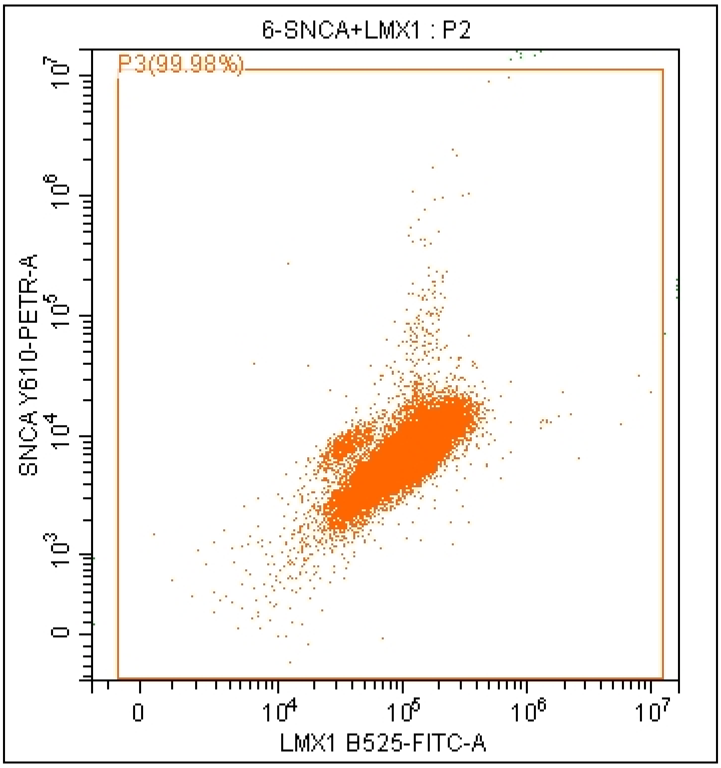

Tube Name: 6-SNCA+LMX1

Sample ID:

Population	Events	% Total	% Parent
All Events	140000	100.00%	100.00%
P1	87840	62.74%	62.74%
P2	84801	60.57%	96.54%
P3	84786	60.56%	99.98%
Q1-UR DP	4	0.00%	0.00%
Q1-UL SNCA only	202	0.14%	0.24%
Q1-LL	84542	60.39%	99.71%
Q1-LR OTX2-LMX1 only	38	0.03%	0.04%
