# Supplementary material for: Identification of an early subset of cerebellar nuclei neurons in mice
Source: eLife. 2024 Dec 16;13:RP93778. doi: 10.7554/eLife.93778 (PMC11649241; doi:10.7554/eLife.93778)
Supplement: Figure 3—source data 2. [file elife-93778-fig3-data2.zip › 190722 Maryam celebellum flow 1.pdf]

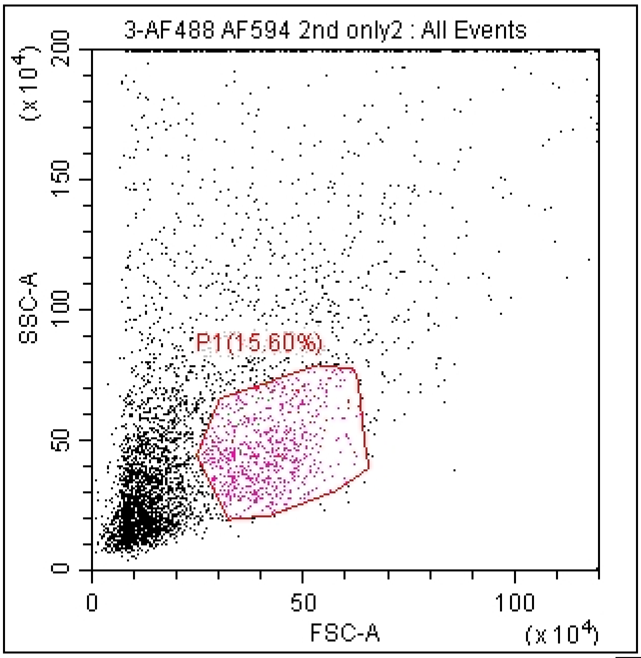

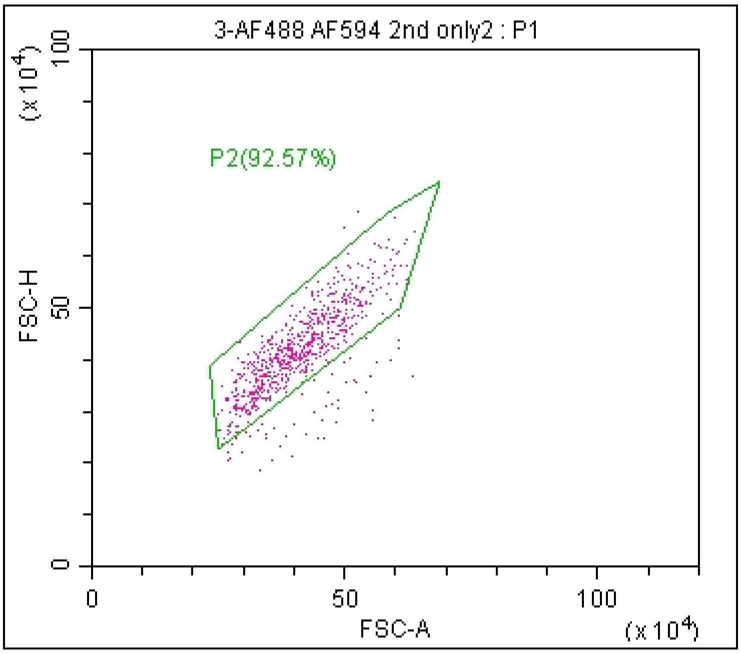

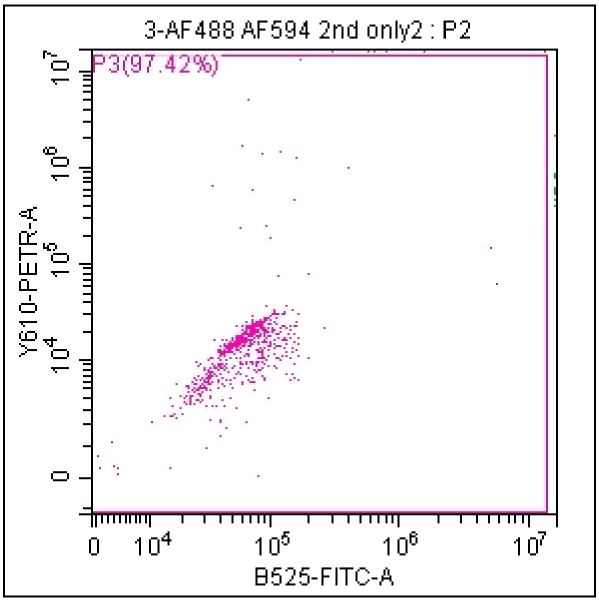

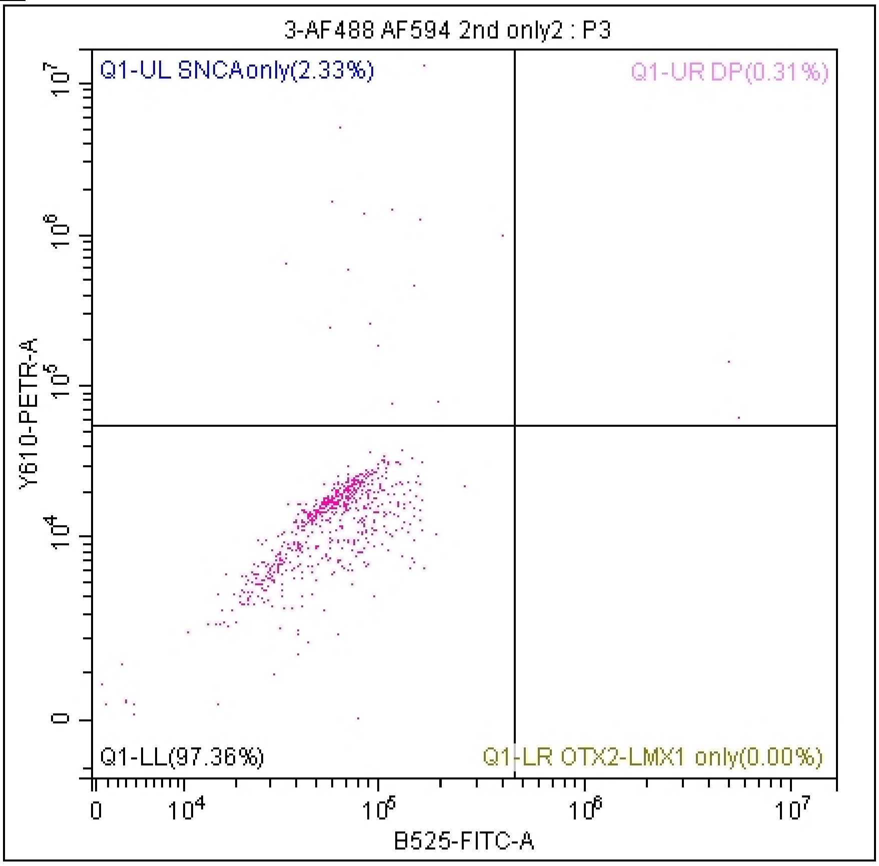

Tube Name: 3-AF488 AF594 2nd only2

Sample ID:

Population	Events	% Total	% Parent
All Events	4570	100.00%	100.00%
P1	713	15.60%	15.60%
P2	660	14.44%	92.57%
P3	643	14.07%	97.42%
Q1-UR DP	2	0.04%	0.31%
Q1-UL SNCA only	15	0.33%	2.33%
Q1-LL	626	13.70%	97.36%
Q1-LR OTX2-LMX1 only	0	0.00%	0.00%

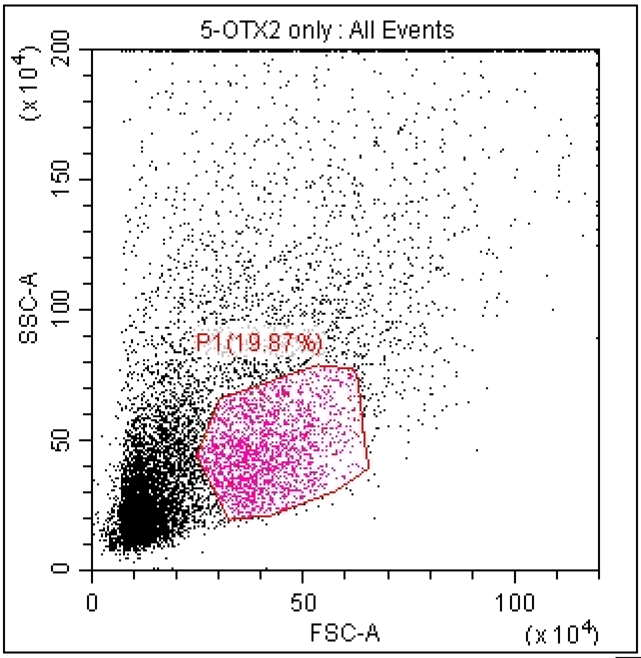

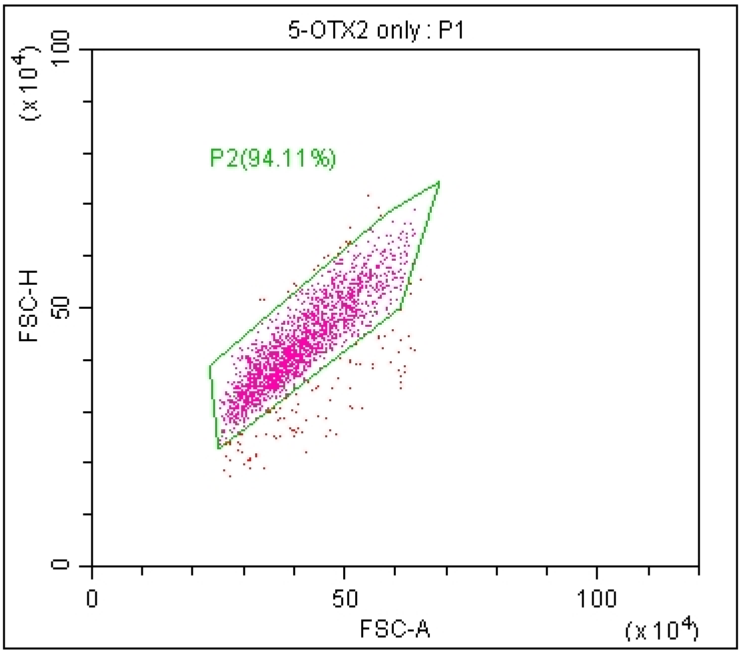

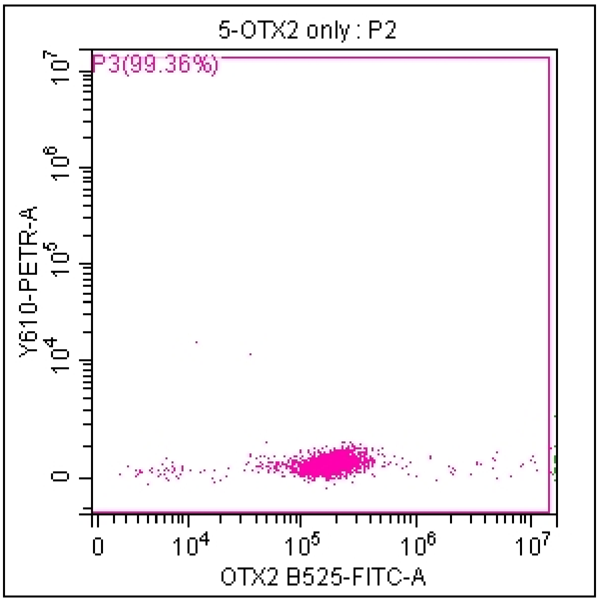

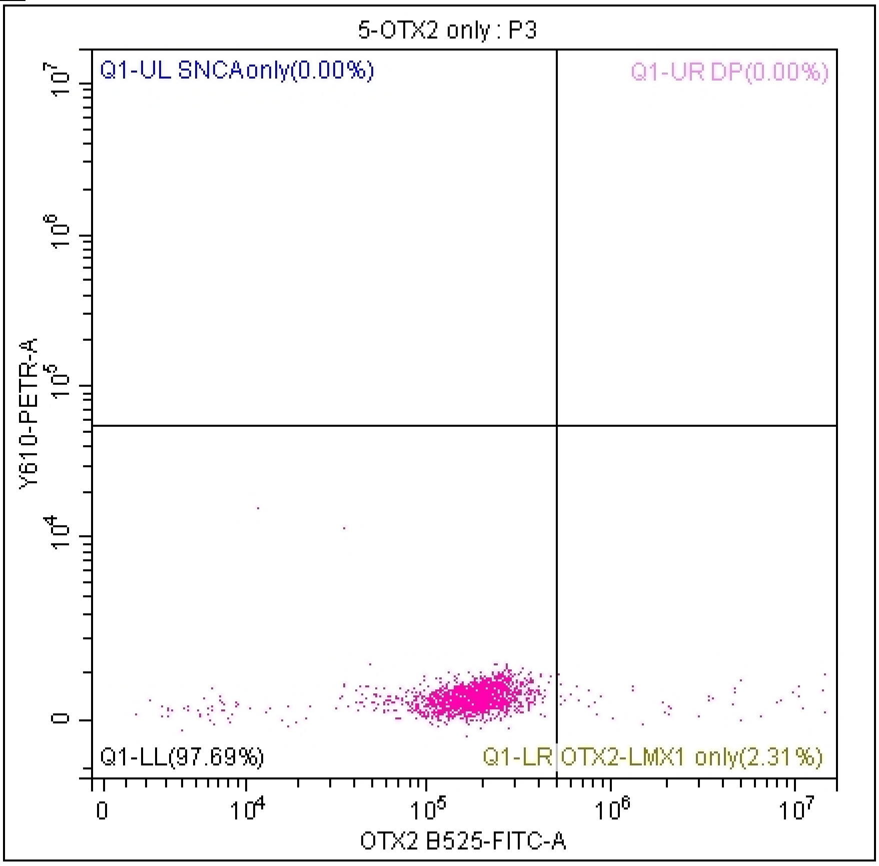

Tube Name: 5-OTX2 only
Sample ID:

Population	Events	% Total	% Parent
All Events	10000	100.00%	100.00%
P1	1987	19.87%	19.87%
P2	1870	18.70%	94.11%
P3	1858	18.58%	99.36%
Q1-UR DP	0	0.00%	0.00%
Q1-UL SNCA only	0	0.00%	0.00%
Q1-LL	1815	18.15%	97.69%
Q1-LR OTX2-LMX1 only	43	0.43%	2.31%

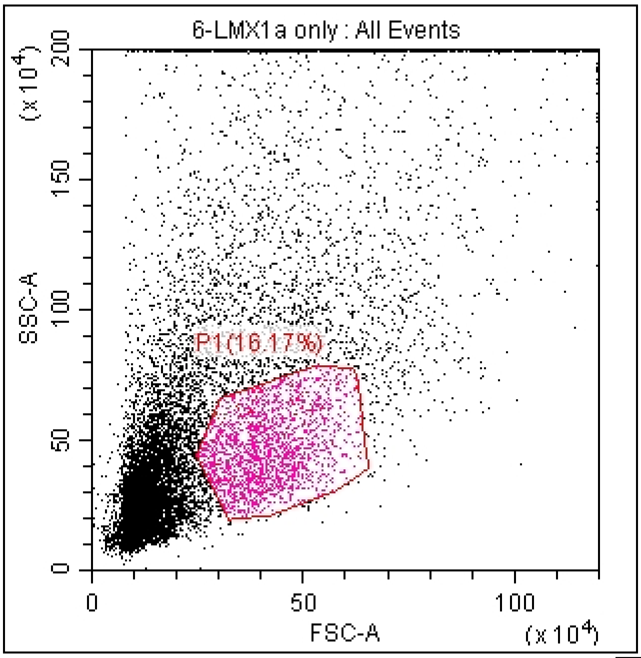

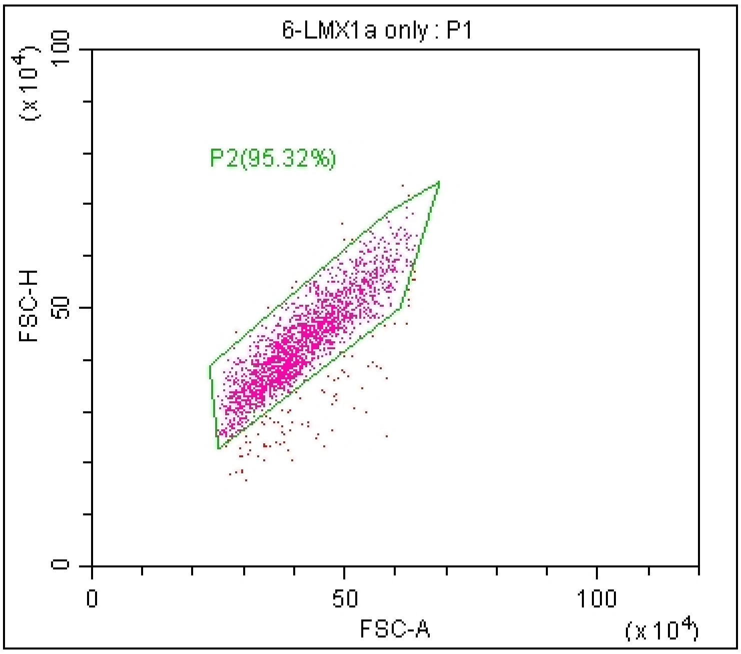

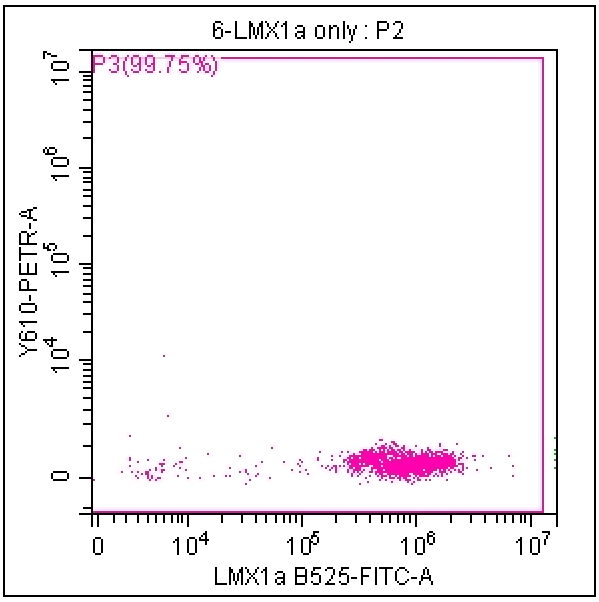

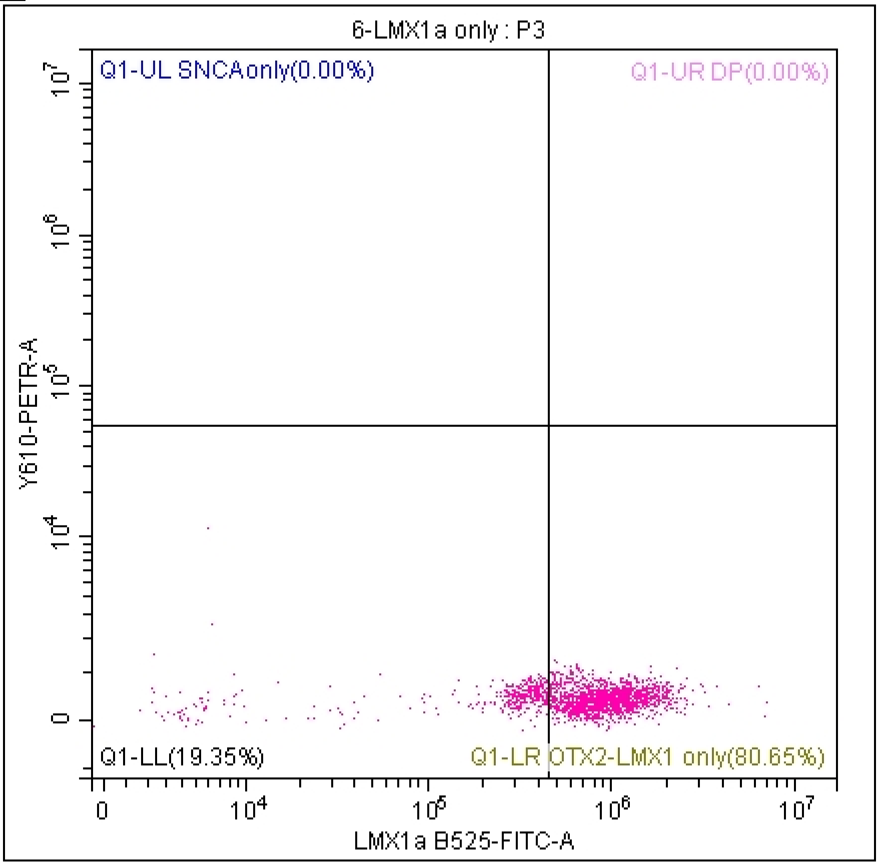

Tube Name: 6-LMX1a only

Sample ID:

Population	Events	% Total	% Parent
All Events	12809	100.00%	100.00%
P1	2071	16.17%	16.17%
P2	1974	15.41%	95.32%
P3	1969	15.37%	99.75%
Q1-UR DP	0	0.00%	0.00%
Q1-UL SNCA only	0	0.00%	0.00%
Q1-LL	381	2.97%	19.35%
Q1-LR OTX2-LMX1 only	1588	12.40%	80.65%

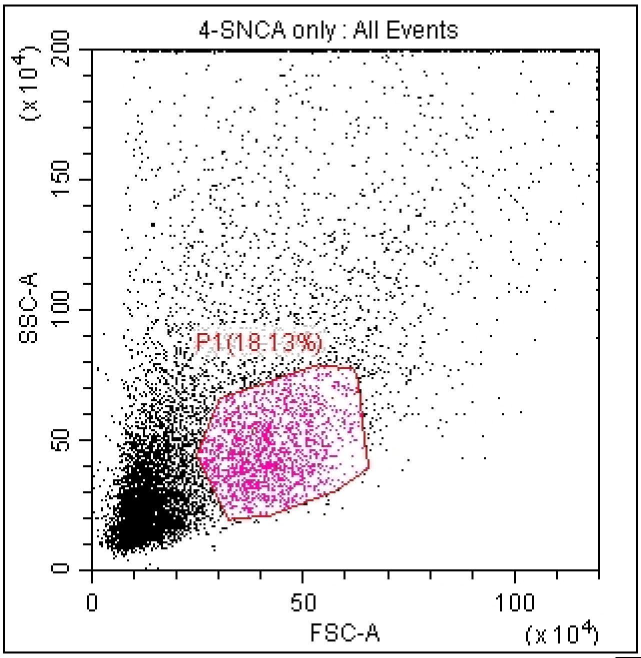

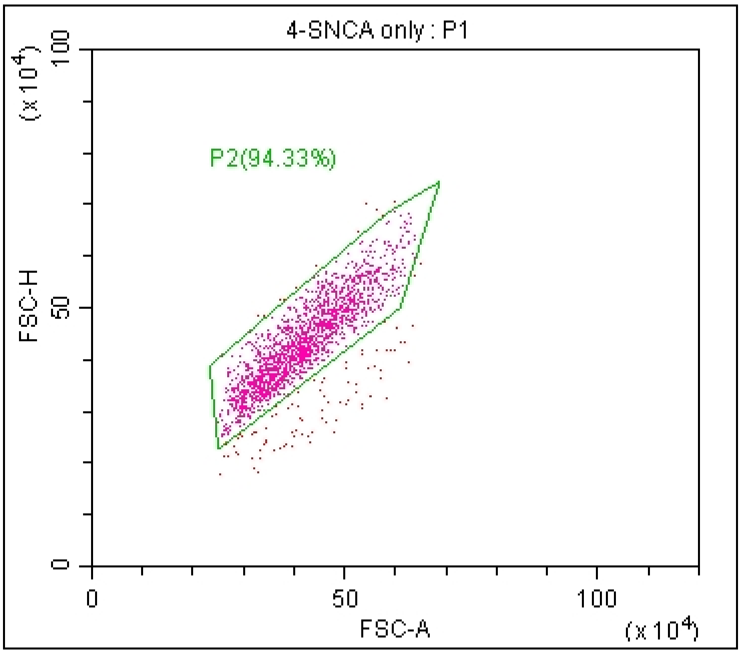

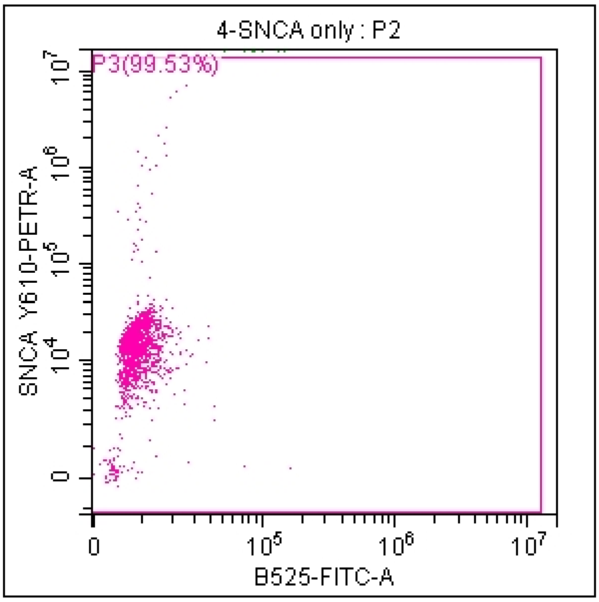

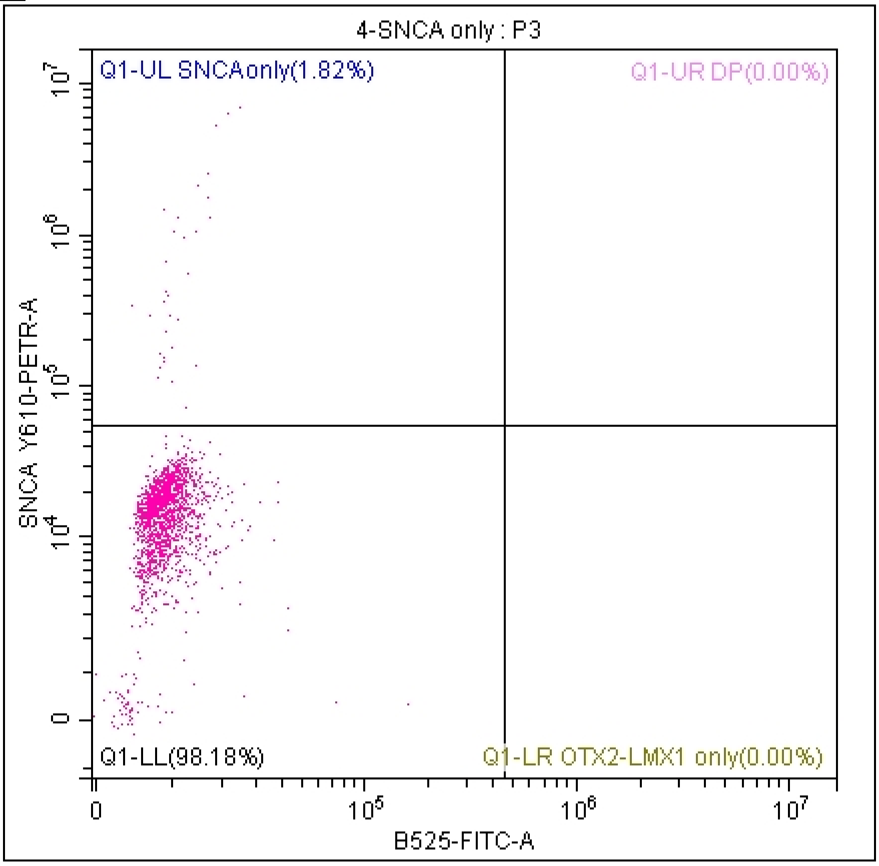

Tube Name: 4-SNCA only

Sample ID:

Population	Events	% Total	% Parent
All Events	10028	100.00%	100.00%
P1	1818	18.13%	18.13%
P2	1715	17.10%	94.33%
P3	1707	17.02%	99.53%
Q1-UR DP	0	0.00%	0.00%
Q1-UL SNCA only	31	0.31%	1.82%
Q1-LL	1676	16.71%	98.18%
Q1-LR OTX2-LMX1 only	0	0.00%	0.00%

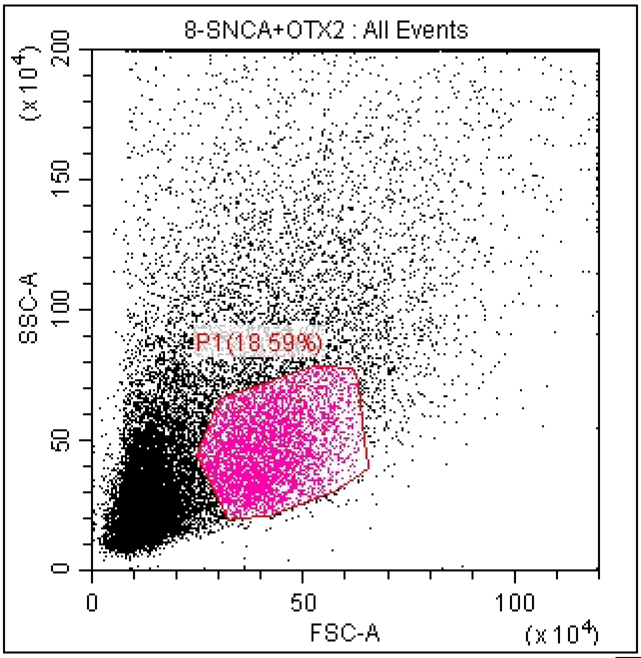

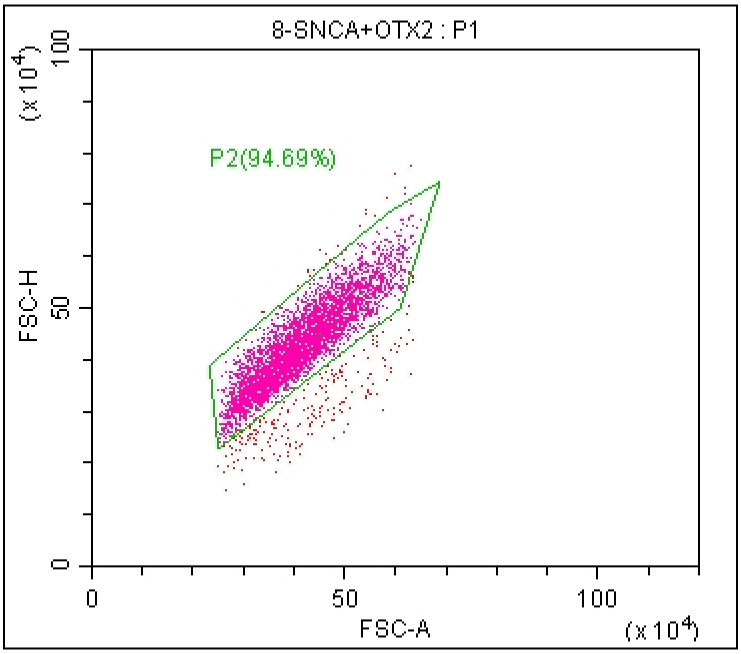

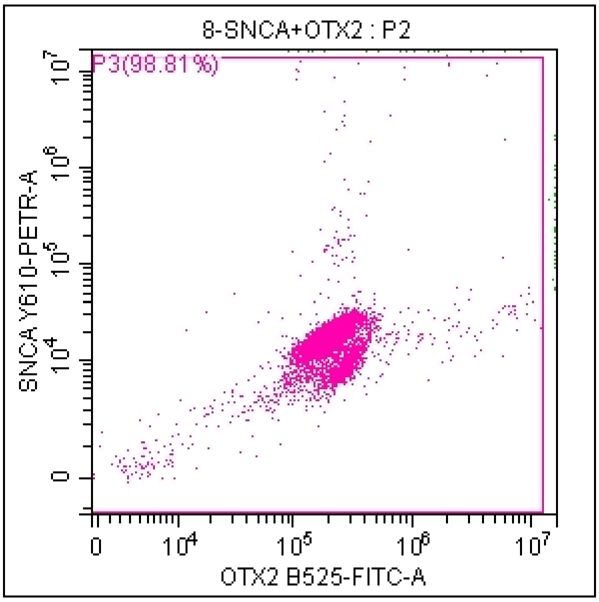

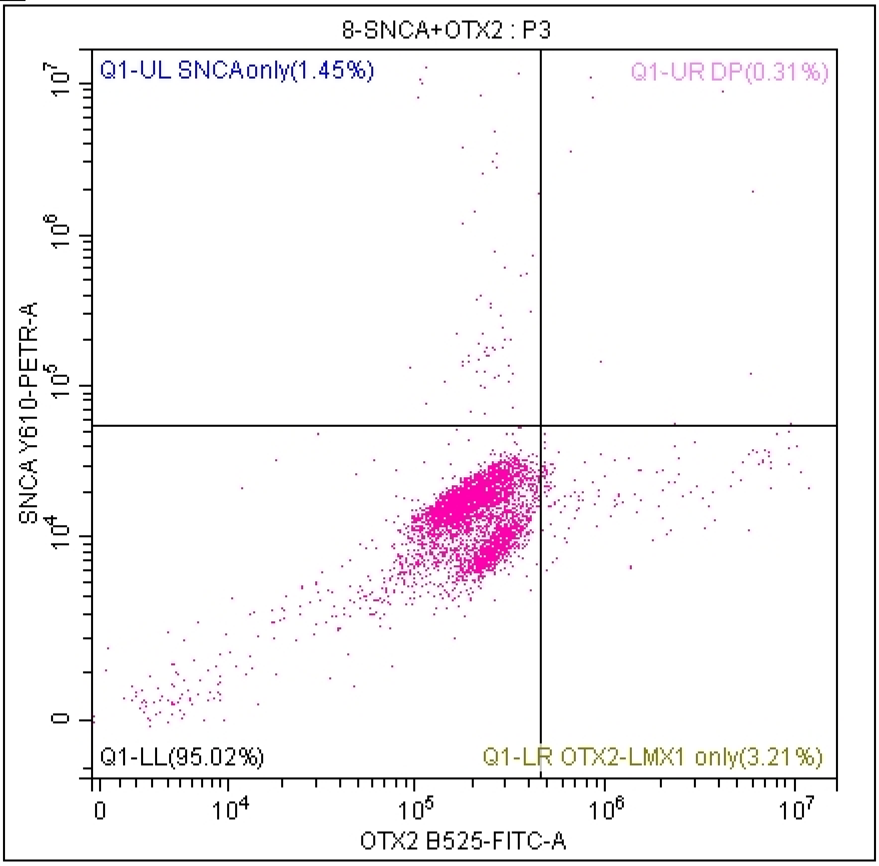

Tube Name: 8-SNCA+OTX2

Sample ID:

Population	Events	% Total	% Parent
All Events	23788	100.00%	100.00%
P1	4422	18.59%	18.59%
P2	4187	17.60%	94.69%
P3	4137	17.39%	98.81%
Q1-UR DP	13	0.05%	0.31%
Q1-UL SNCA only	60	0.25%	1.45%
Q1-LL	3931	16.53%	95.02%
Q1-LR OTX2-LMX1 only	133	0.56%	3.21%

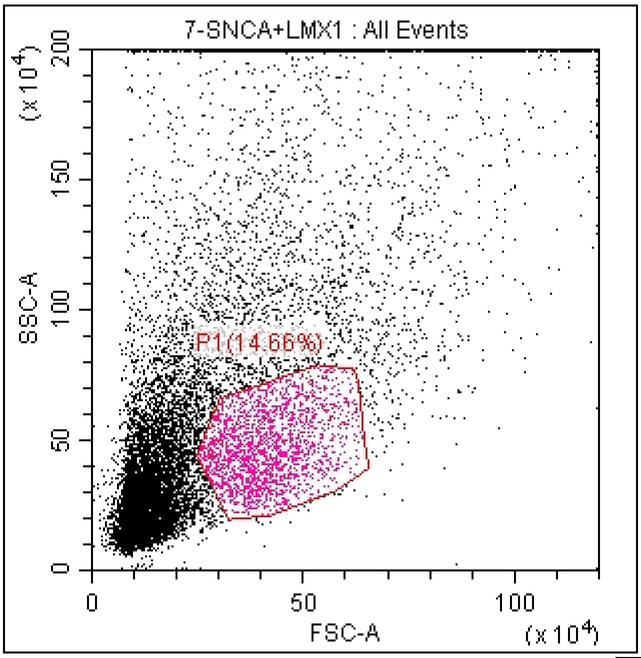

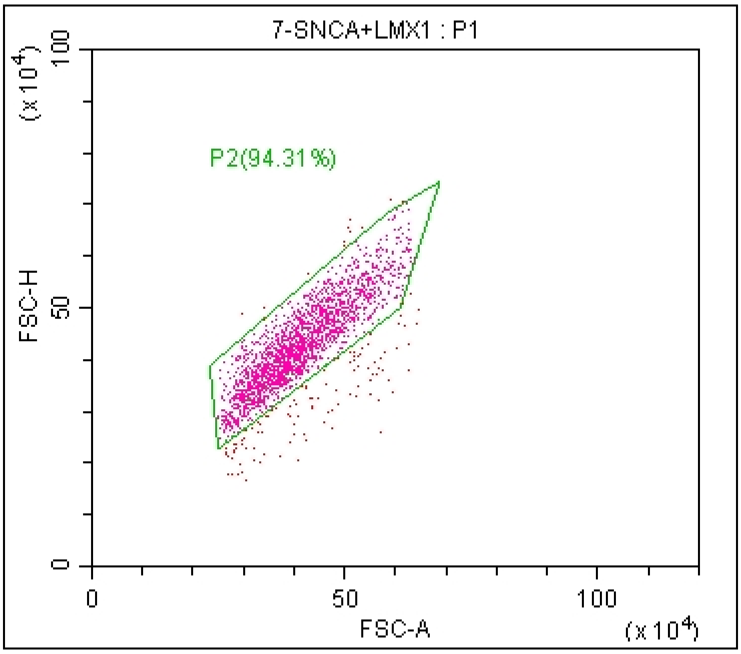

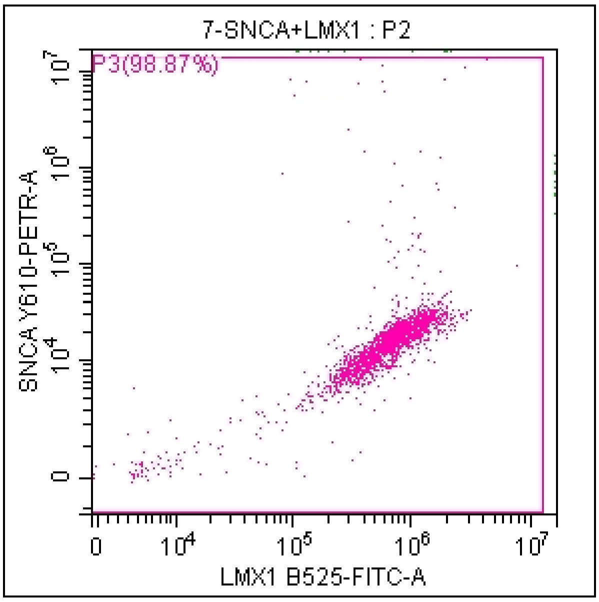

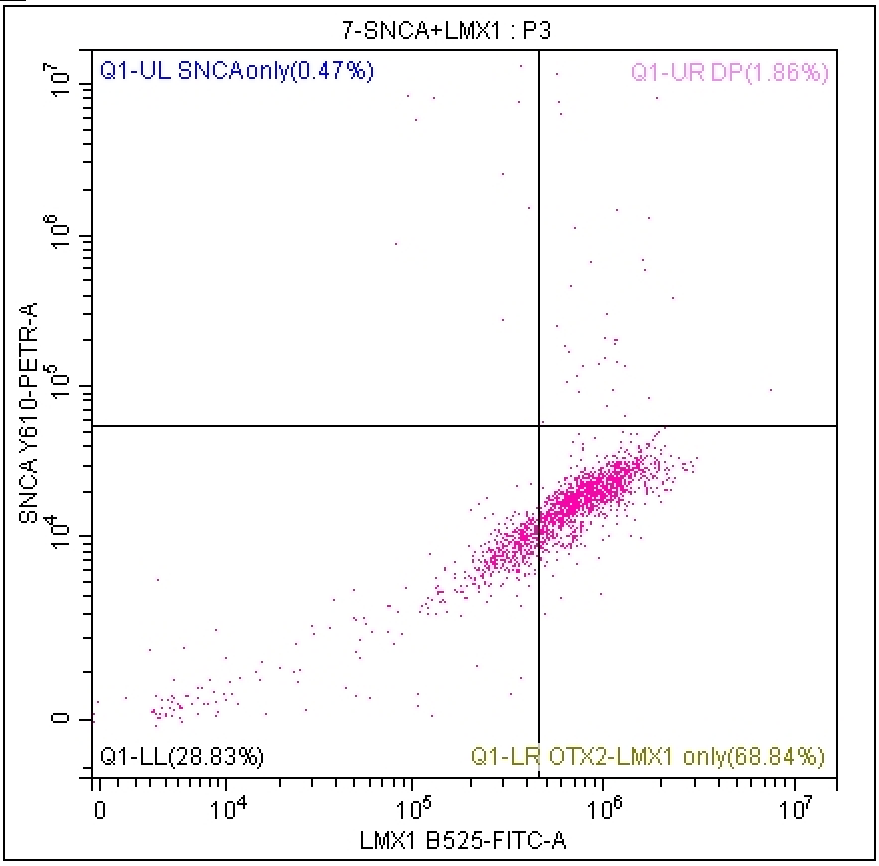

Tube Name: 7-SNCA+LMX1

Sample ID:

Population	Events	% Total	% Parent
All Events	14135	100.00%	100.00%
P1	2072	14.66%	14.66%
P2	1954	13.82%	94.31%
P3	1932	13.67%	98.87%
Q1-UR DP	36	0.25%	1.86%
Q1-UL SNCA only	9	0.06%	0.47%
Q1-LL	557	3.94%	28.83%
Q1-LR OTX2-LMX1 only	1330	9.41%	68.84%
