# Supplementary material for: Identification of an early subset of cerebellar nuclei neurons in mice
Source: eLife. 2024 Dec 16;13:RP93778. doi: 10.7554/eLife.93778 (PMC11649241; doi:10.7554/eLife.93778)
Supplement: Figure 3—source data 2. [file elife-93778-fig3-data2.zip › 190726 Maryam Celebellum flow.pdf]

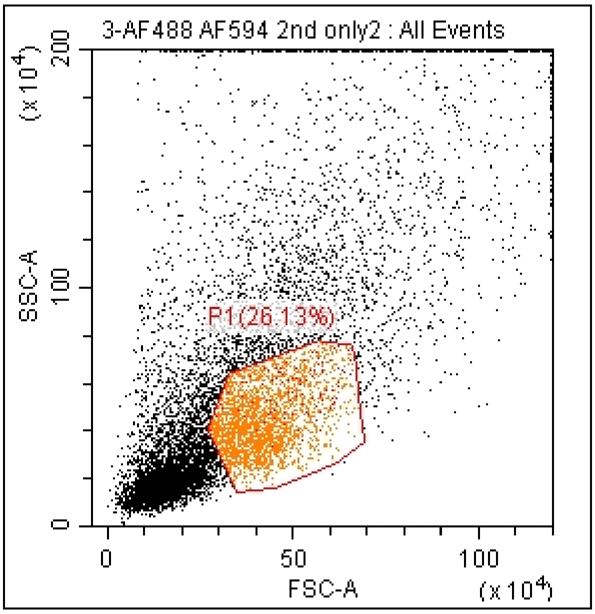

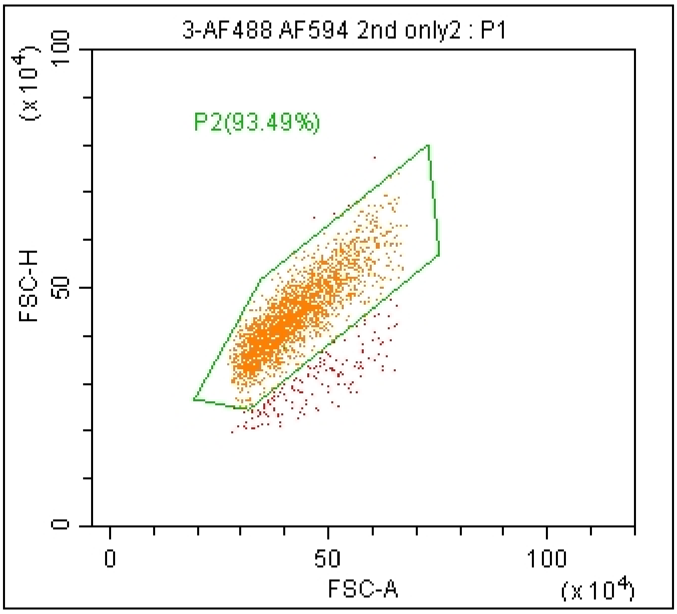

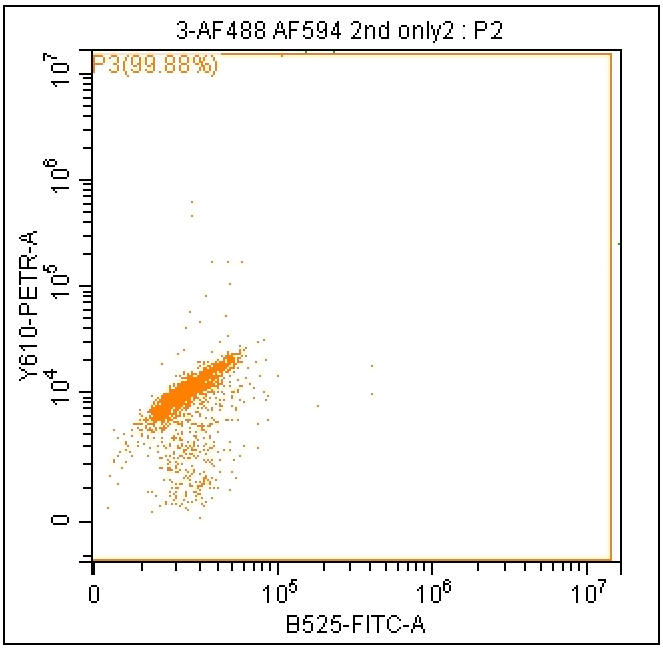

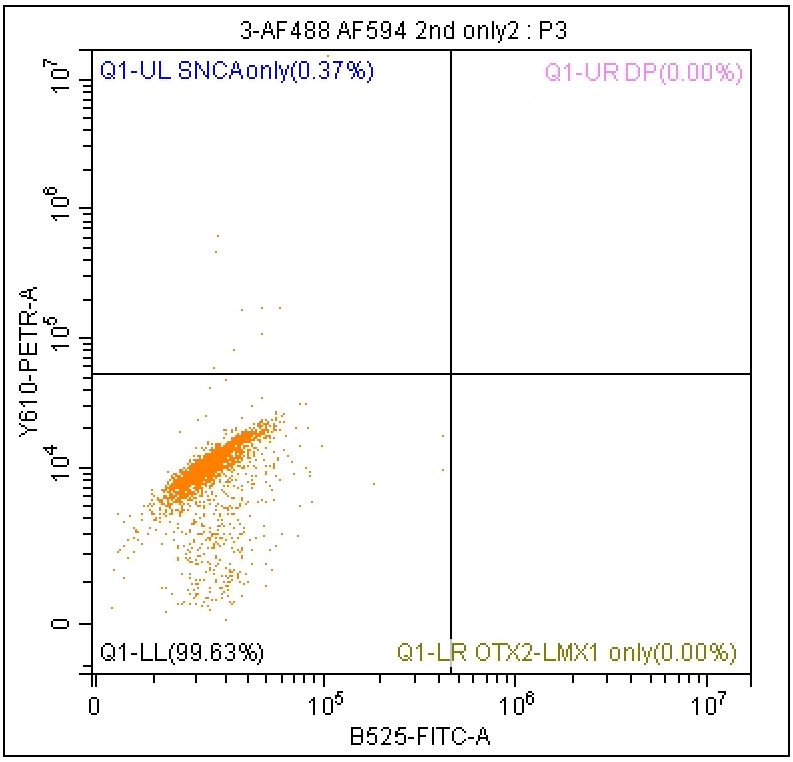

Tube Name: 3-AF488 AF594 2nd only2

Sample ID:

Population	Events	% Total	% Parent
All Events	10000	100.00%	100.00%
P1	2613	26.13%	26.13%
P2	2443	24.43%	93.49%
P3	2440	24.40%	99.88%
Q1-UR DP	0	0.00%	0.00%
Q1-UL SNCA only	9	0.09%	0.37%
Q1-LL	2431	24.31%	99.63%
Q1-LR OTX2-LMX1 only	0	0.00%	0.00%

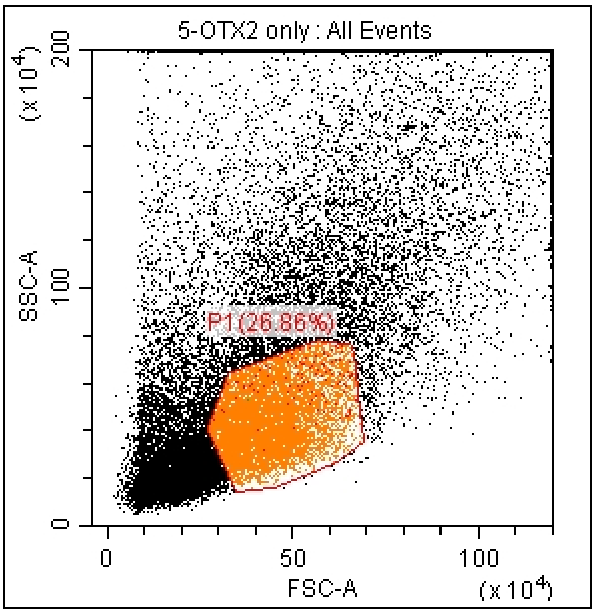

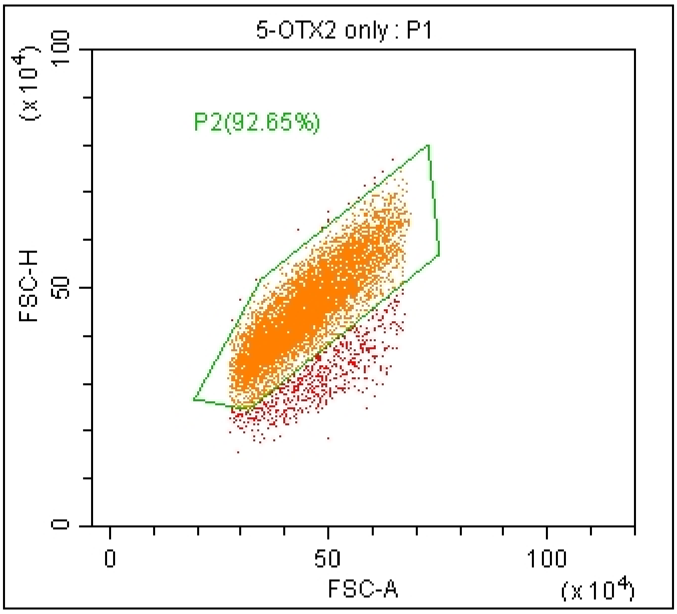

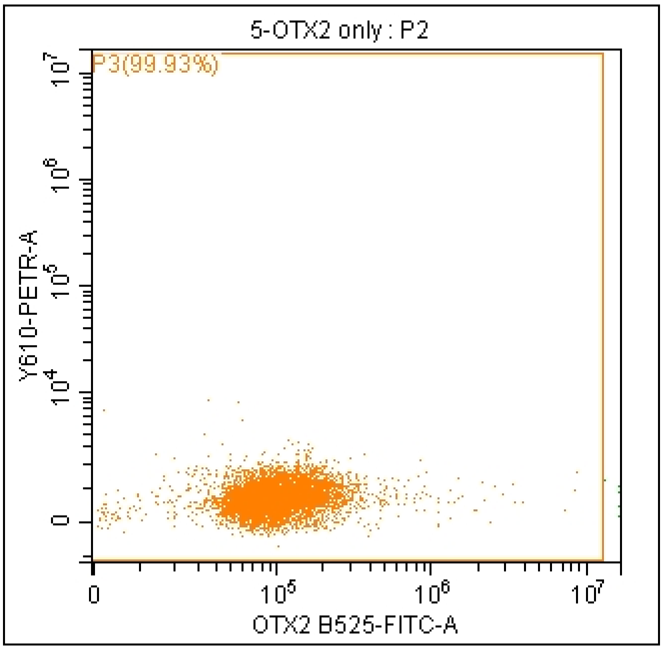

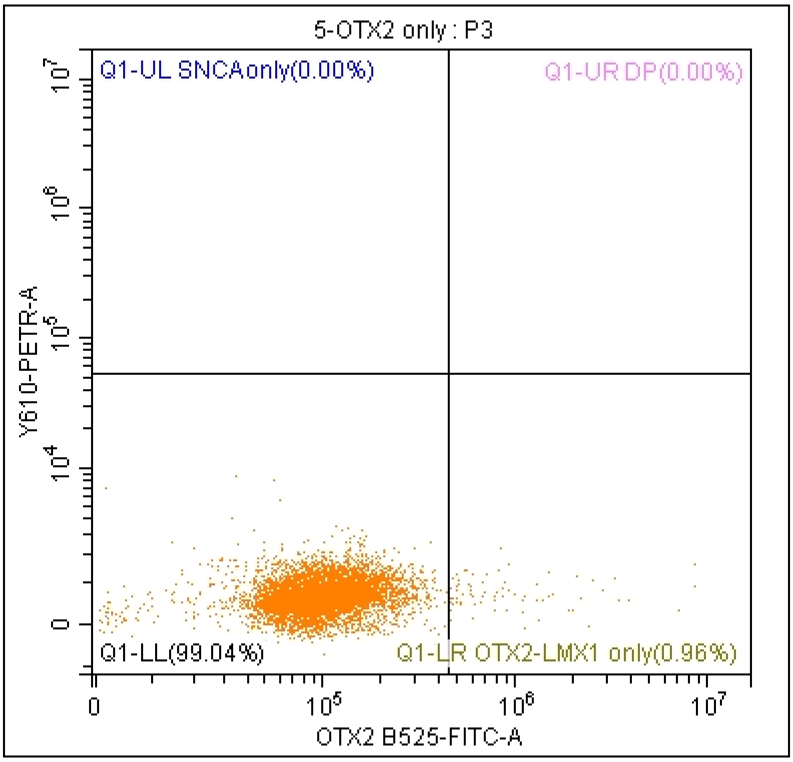

Tube Name: 5-OTX2 only

Sample ID:

Population	Events	% Total	% Parent
All Events	30000	100.00%	100.00%
P1	8059	26.86%	26.86%
P2	7467	24.89%	92.65%
P3	7462	24.87%	99.93%
Q1-UR DP	0	0.00%	0.00%
Q1-UL SNCA only	0	0.00%	0.00%
Q1-LL	7390	24.63%	99.04%
Q1-LR OTX2-LMX1 only	72	0.24%	0.96%

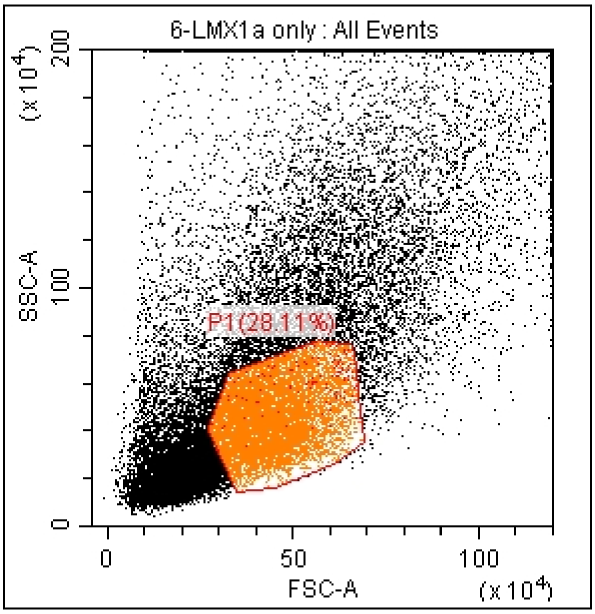

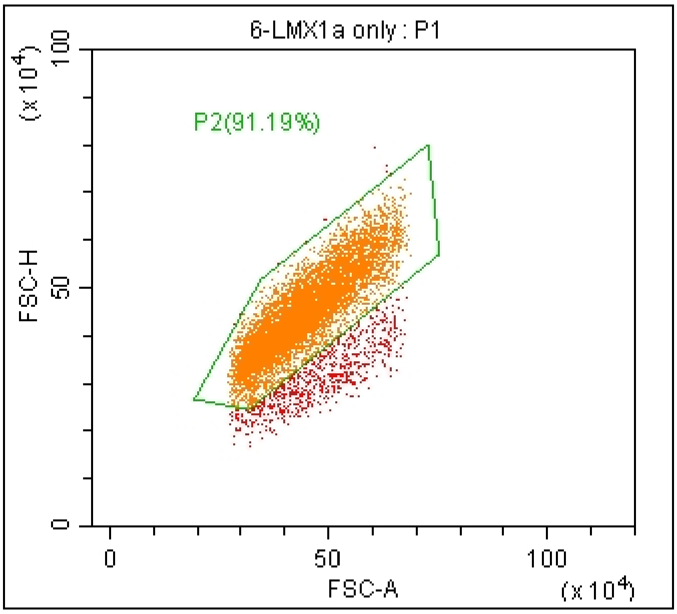

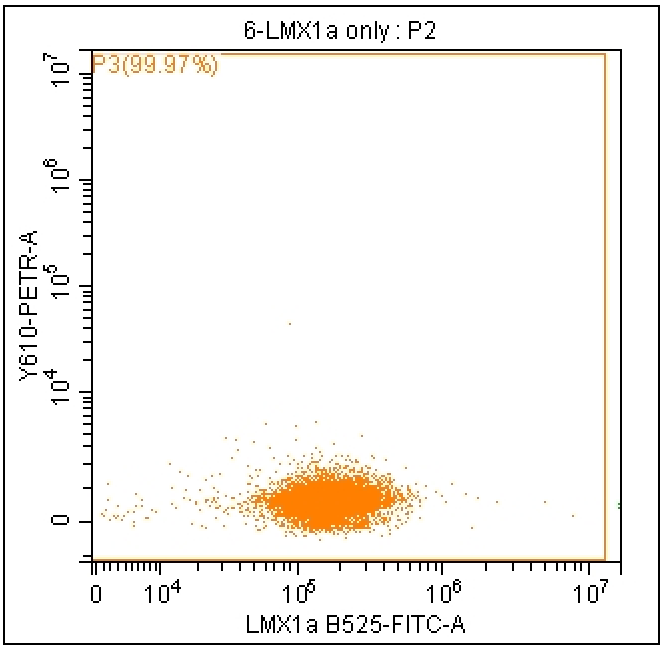

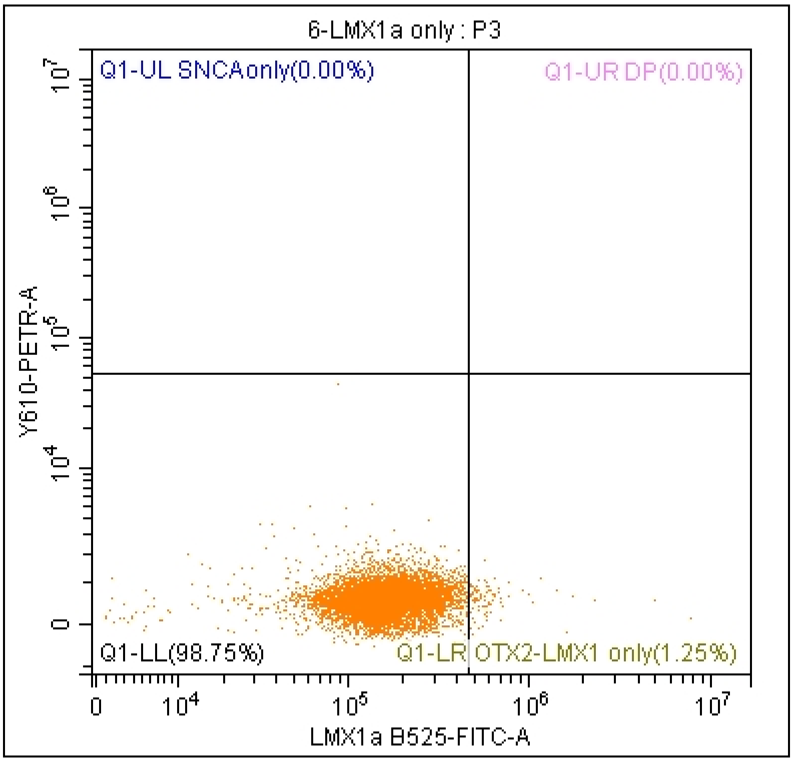

Tube Name: 6-LMX1a only

Sample ID:

Population	Events	% Total	% Parent
All Events	30000	100.00%	100.00%
P1	8433	28.11%	28.11%
P2	7690	25.63%	91.19%
P3	7688	25.63%	99.97%
Q1-UR DP	0	0.00%	0.00%
Q1-UL SNCA only	0	0.00%	0.00%
Q1-LL	7592	25.31%	98.75%
Q1-LR OTX2-LMX1 only	96	0.32%	1.25%

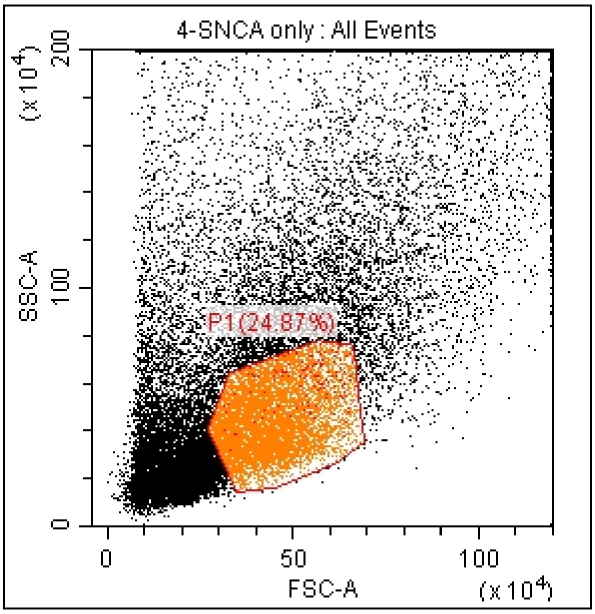

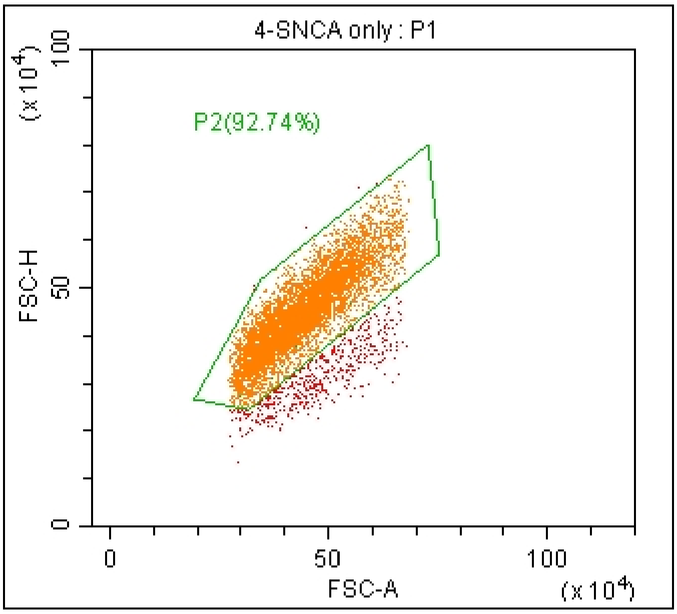

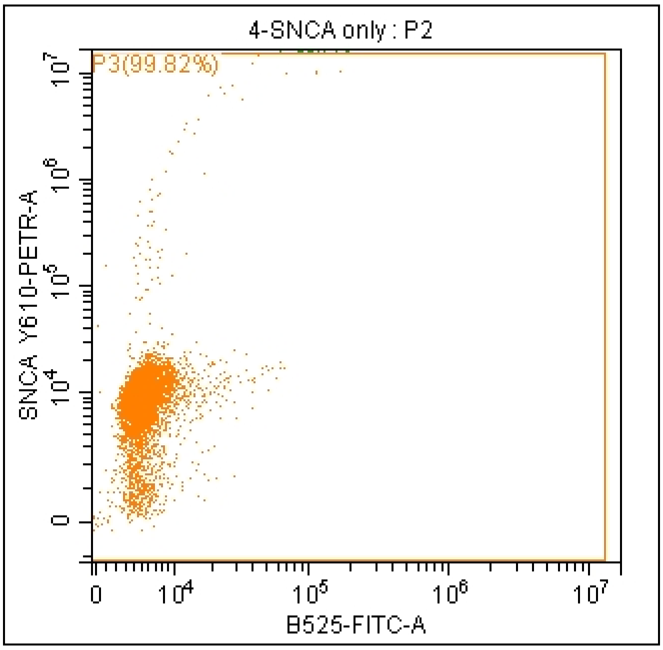

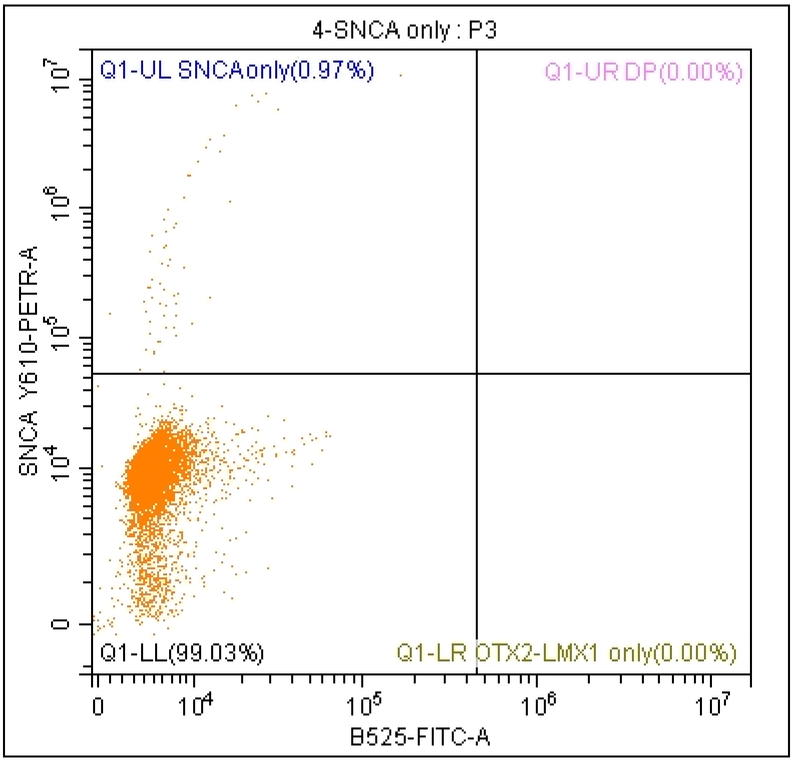

Tube Name: 4-SNCA only

Sample ID:

Population	Events	% Total	% Parent
All Events	28140	100.00%	100.00%
P1	6998	24.87%	24.87%
P2	6490	23.06%	92.74%
P3	6478	23.02%	99.82%
Q1-UR DP	0	0.00%	0.00%
Q1-UL SNCA only	63	0.22%	0.97%
Q1-LL	6415	22.80%	99.03%
Q1-LR OTX2-LMX1 only	0	0.00%	0.00%

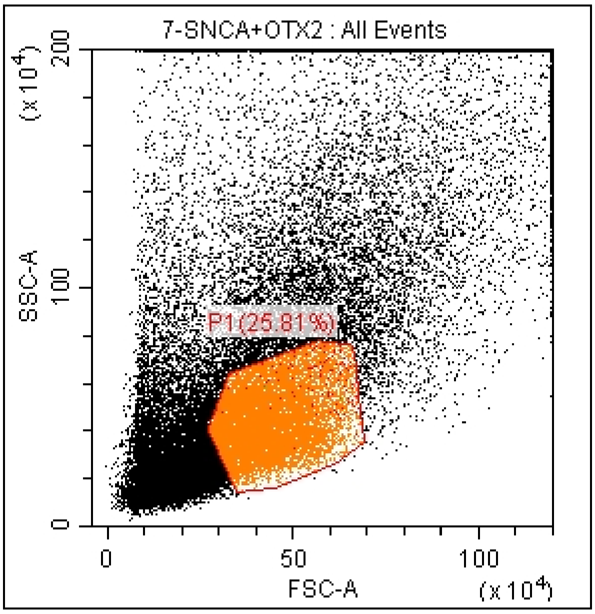

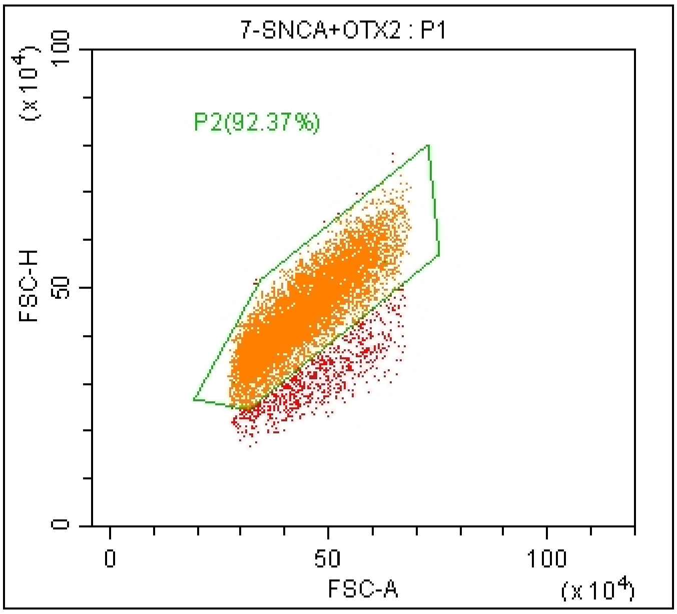

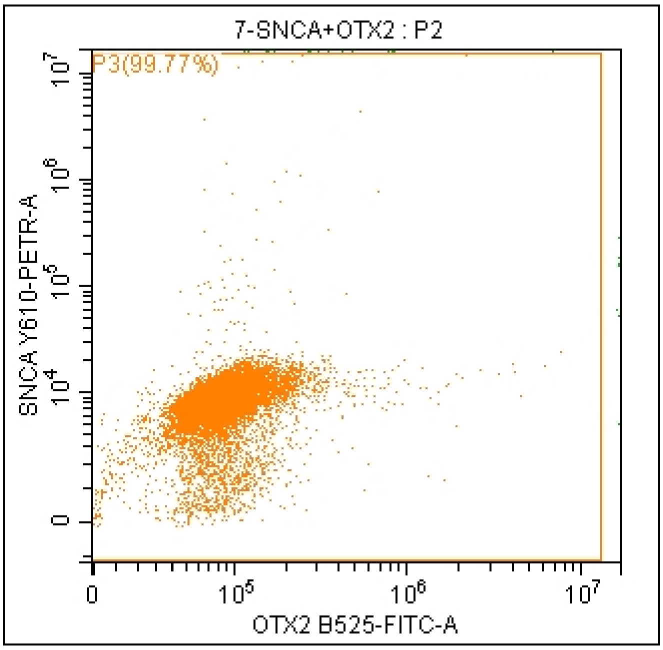

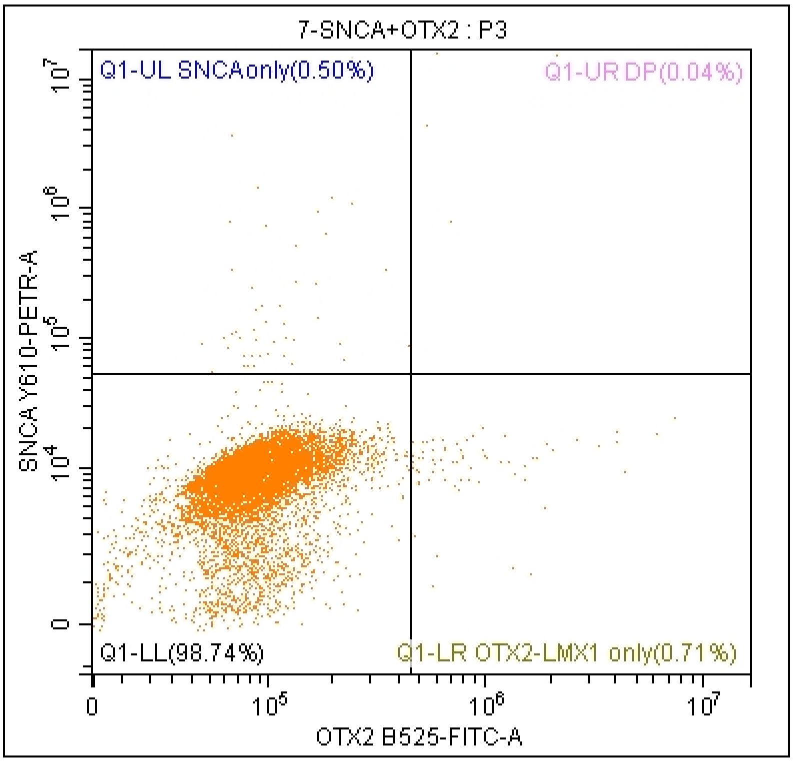

Tube Name: 7-SNCA+OTX2

Sample ID:

Population	Events	% Total	% Parent
All Events	40000	100.00%	100.00%
P1	10325	25.81%	25.81%
P2	9537	23.84%	92.37%
P3	9515	23.79%	99.77%
Q1-UR DP	4	0.01%	0.04%
Q1-UL SNCA only	48	0.12%	0.50%
Q1-LL	9395	23.49%	98.74%
Q1-LR OTX2-LMX1 only	68	0.17%	0.71%

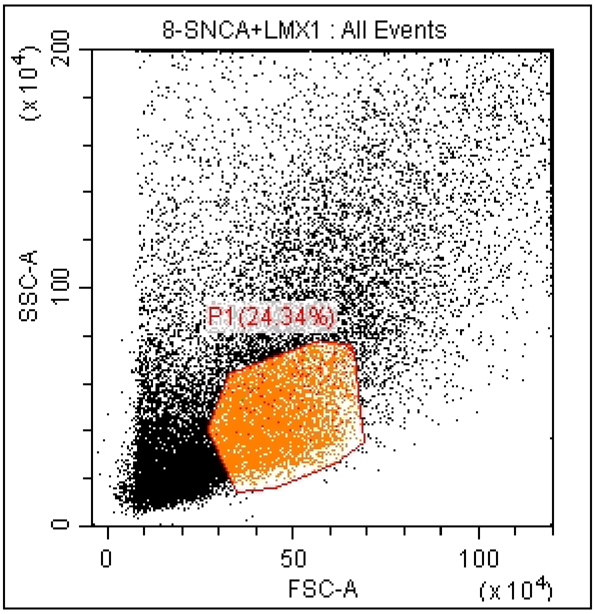

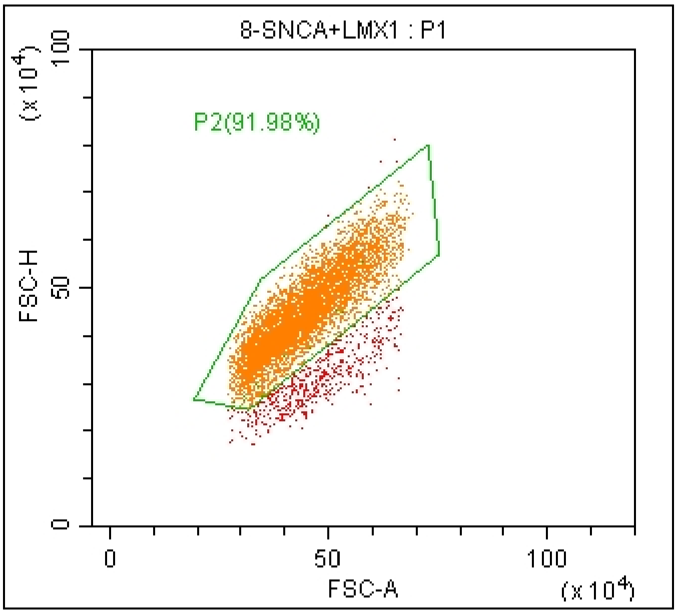

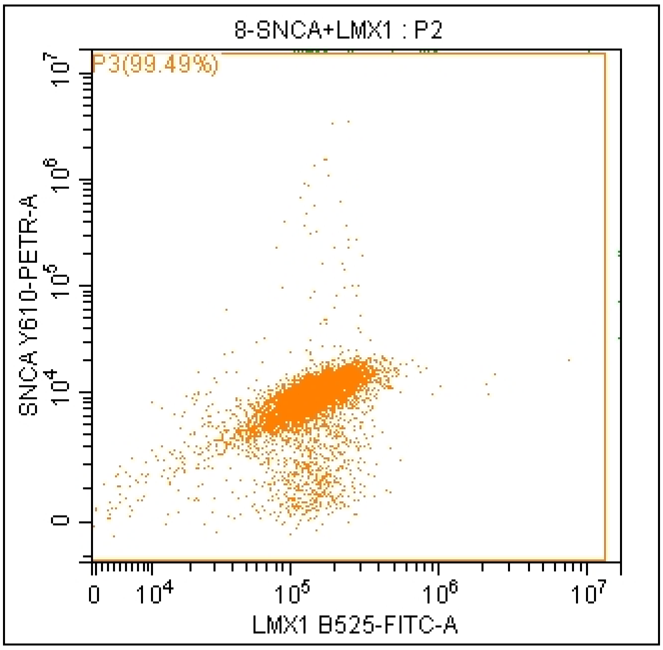

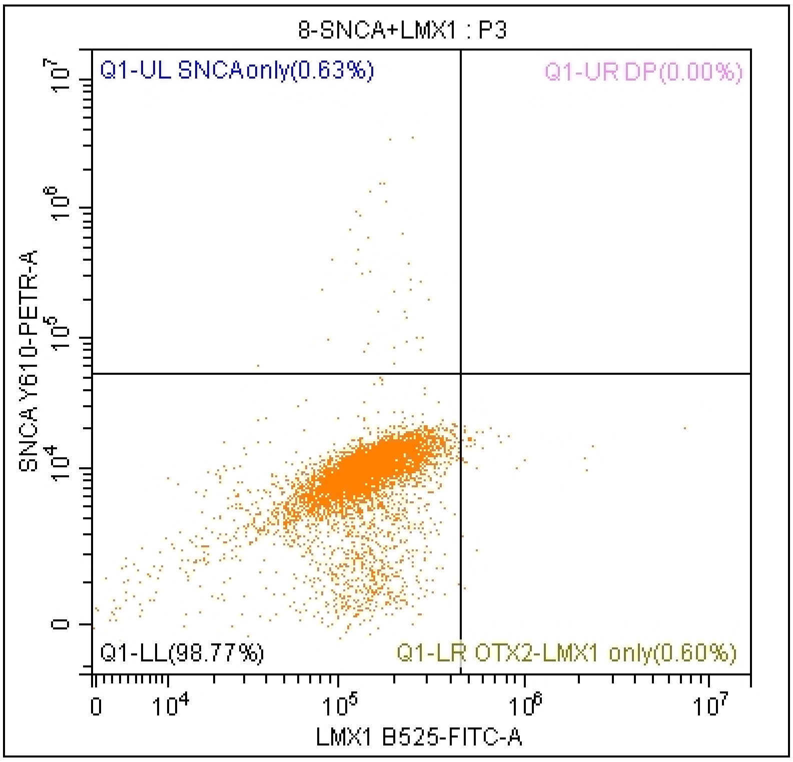

Tube Name: 8-SNCA+LMX1

Sample ID:

Population	Events	% Total	% Parent
All Events	26328	100.00%	100.00%
P1	6408	24.34%	24.34%
P2	5894	22.39%	91.98%
P3	5864	22.27%	99.49%
Q1-UR DP	0	0.00%	0.00%
Q1-UL SNCA only	37	0.14%	0.63%
Q1-LL	5792	22.00%	98.77%
Q1-LR OTX2-LMX1 only	35	0.13%	0.60%
