# Supplementary material for: Identification of an early subset of cerebellar nuclei neurons in mice
Source: eLife. 2024 Dec 16;13:RP93778. doi: 10.7554/eLife.93778 (PMC11649241; doi:10.7554/eLife.93778)
Supplement: Figure 3—source data 2. [file elife-93778-fig3-data2.zip › 190729 Maryam Celebellum flow.pdf]

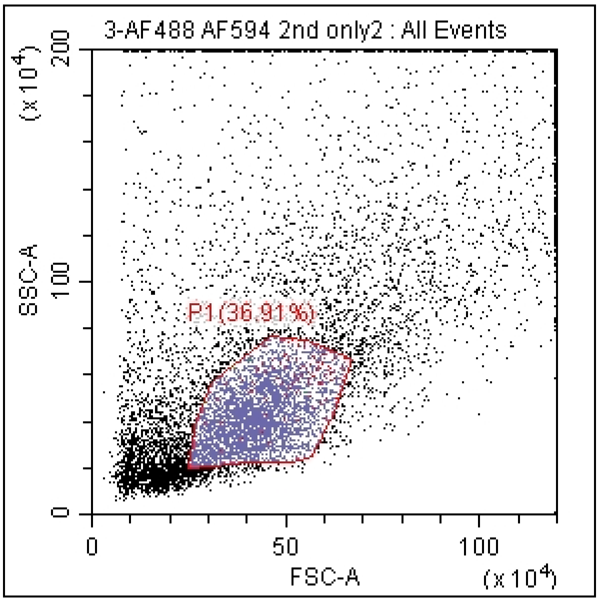

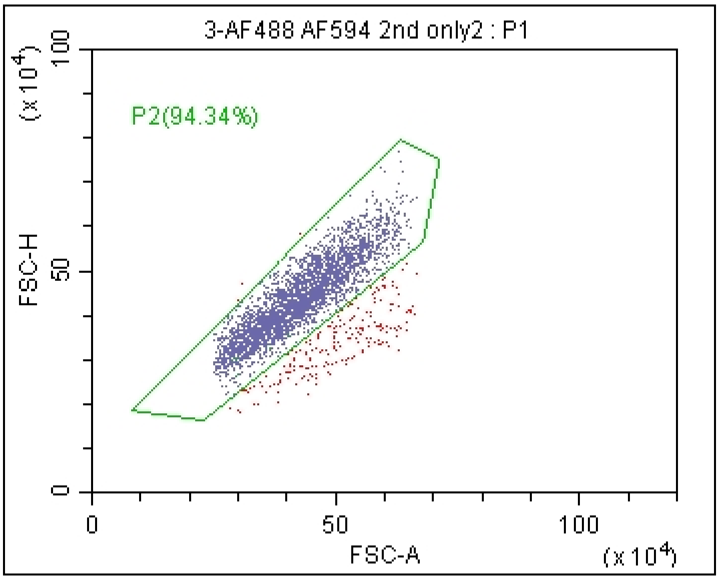

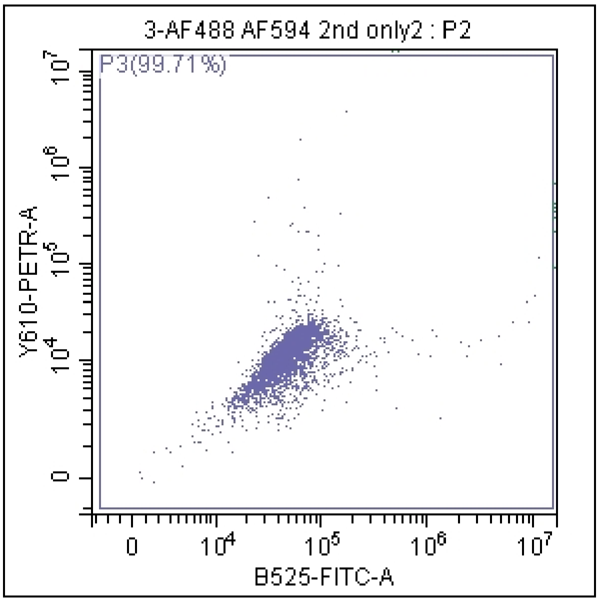

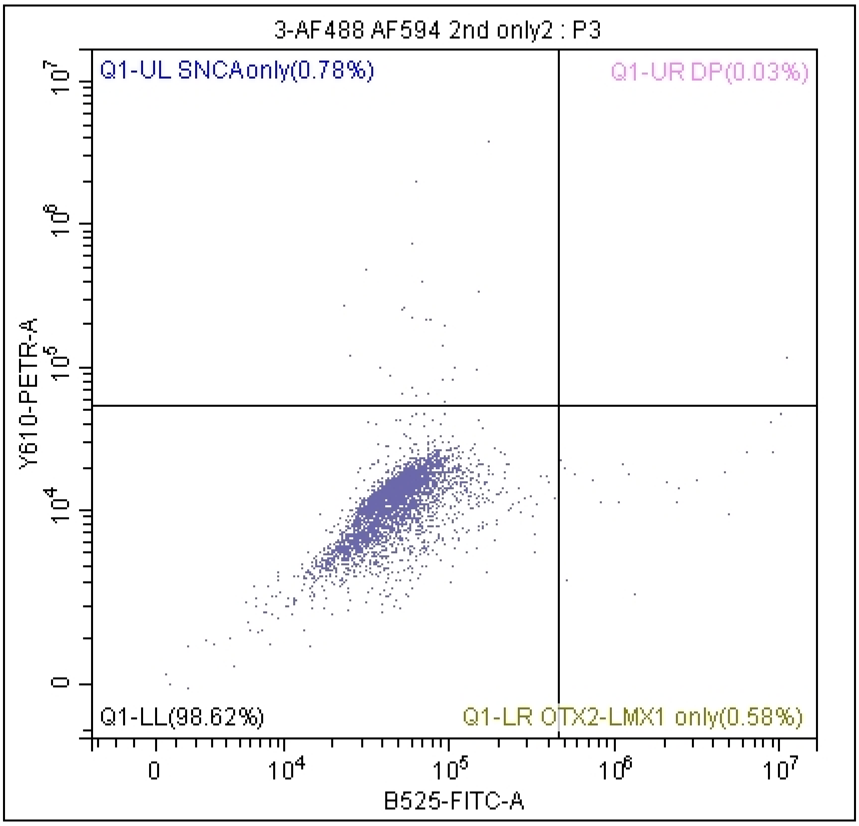

Tube Name: 3-AF488 AF594 2nd only2

Sample ID:

Population	Events	% Total	% Parent
All Events	10000	100.00%	100.00%
P1	3691	36.91%	36.91%
P2	3482	34.82%	94.34%
P3	3472	34.72%	99.71%
Q1-UR DP	1	0.01%	0.03%
Q1-UL SNCA only	27	0.27%	0.78%
Q1-LL	3424	34.24%	98.62%
Q1-LR OTX2-LMX1 only	20	0.20%	0.58%

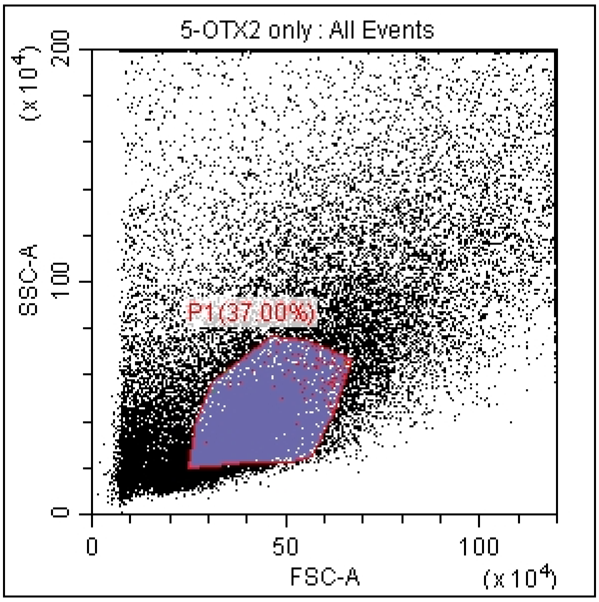

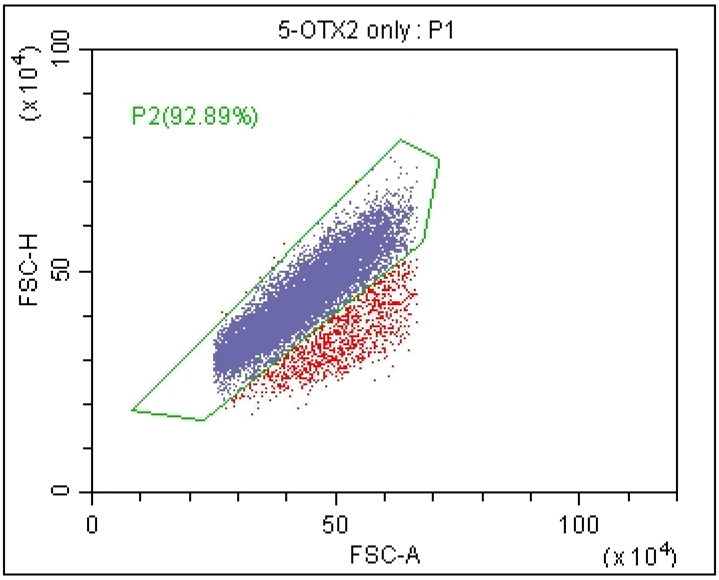

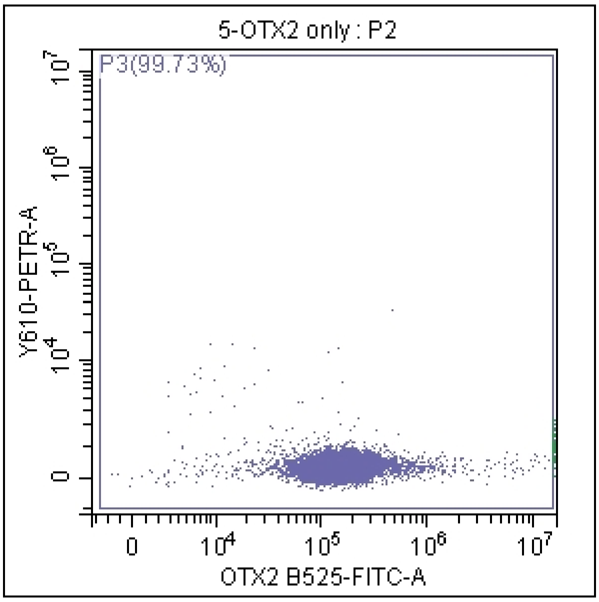

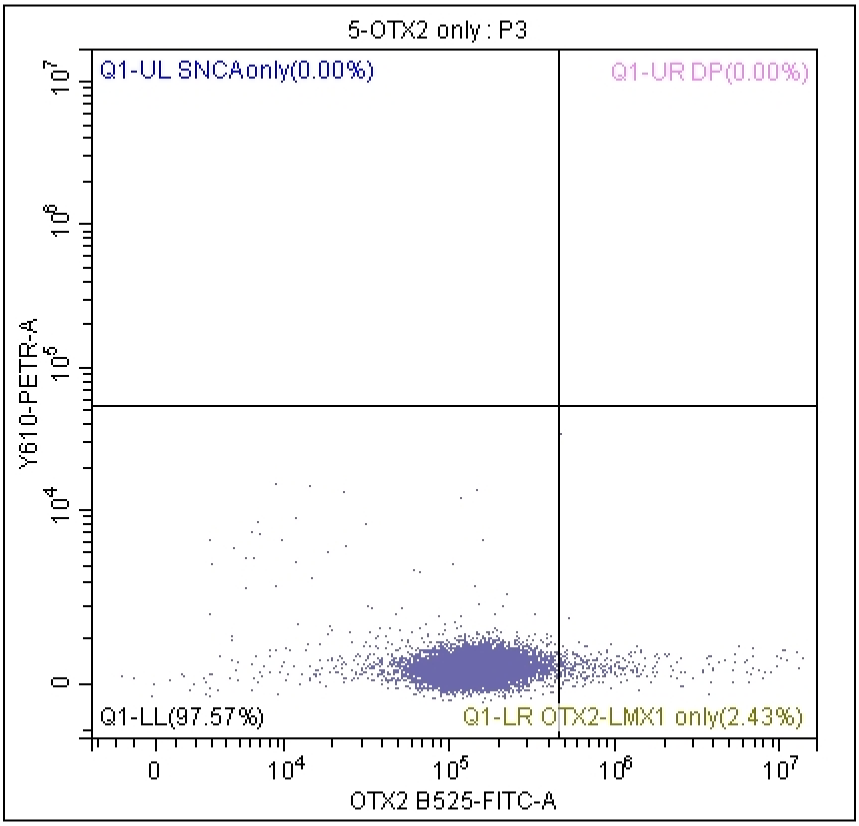

Tube Name: 5-OTX2 only

Sample ID:

Population	Events	% Total	% Parent
All Events	40000	100.00%	100.00%
P1	14800	37.00%	37.00%
P2	13748	34.37%	92.89%
P3	13711	34.28%	99.73%
Q1-UR DP	0	0.00%	0.00%
Q1-UL SNCA only	0	0.00%	0.00%
Q1-LL	13378	33.45%	97.57%
Q1-LR OTX2-LMX1 only	333	0.83%	2.43%

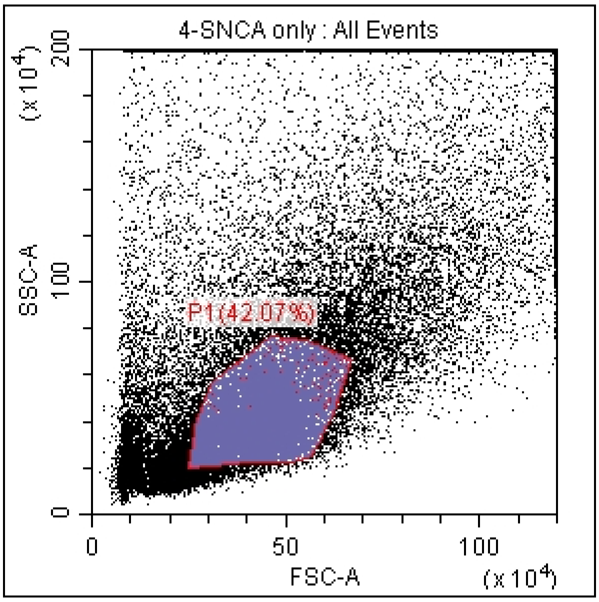

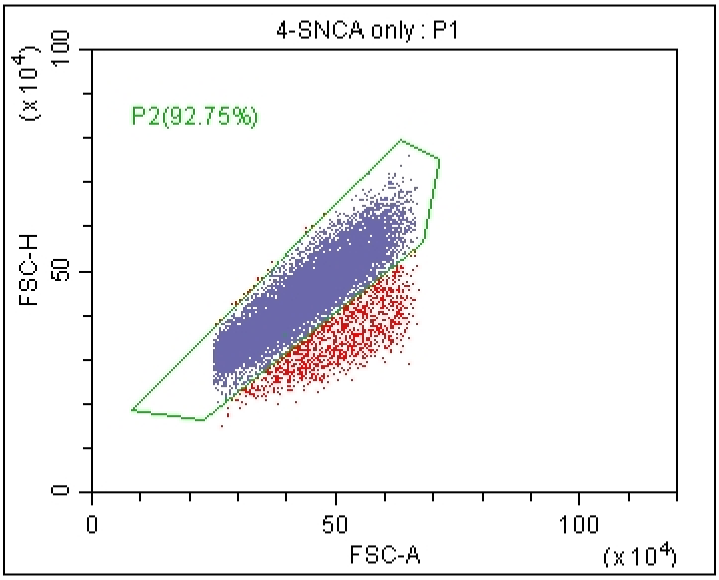

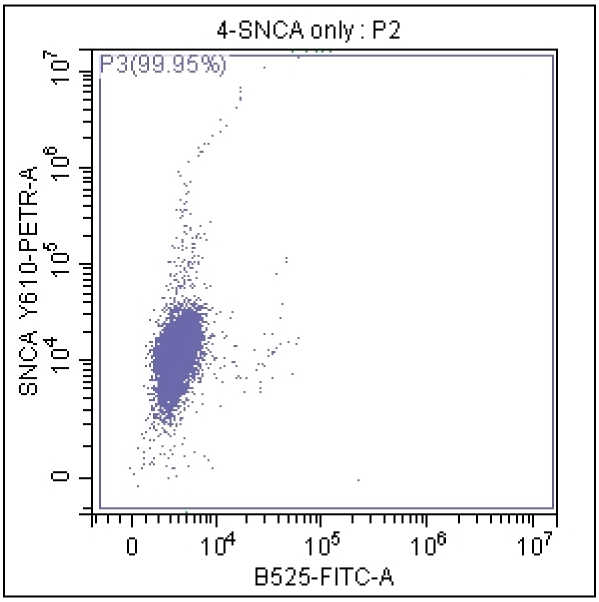

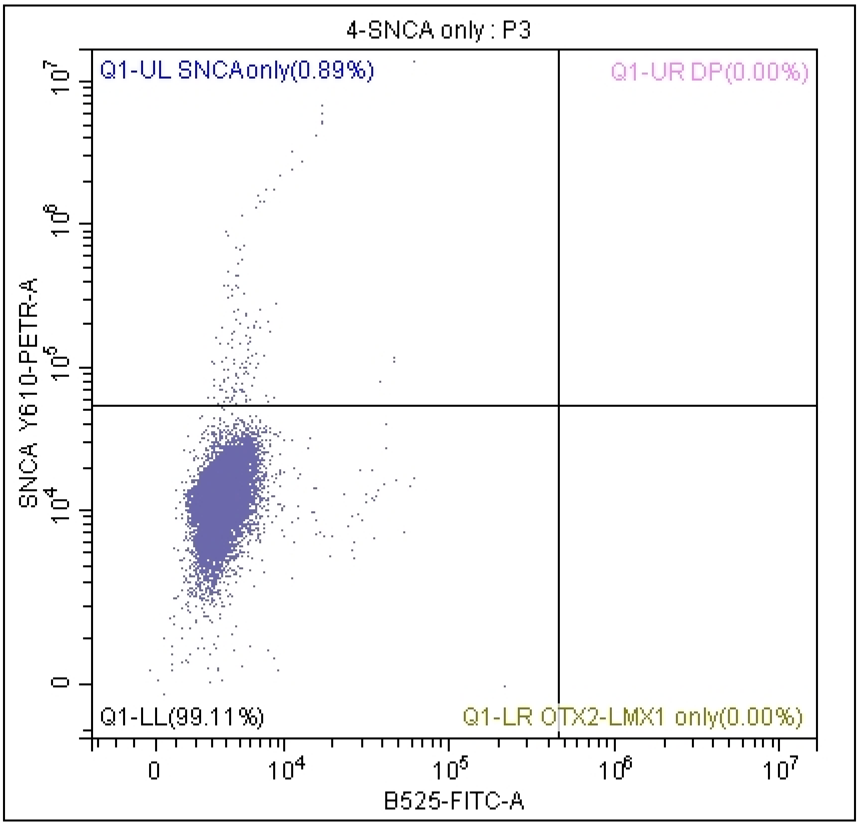

Tube Name: 4-SNCA only

Sample ID:

Population	Events	% Total	% Parent
All Events	40000	100.00%	100.00%
P1	16827	42.07%	42.07%
P2	15607	39.02%	92.75%
P3	15599	39.00%	99.95%
Q1-UR DP	0	0.00%	0.00%
Q1-UL SNCA only	139	0.35%	0.89%
Q1-LL	15460	38.65%	99.11%
Q1-LR OTX2-LMX1 only	0	0.00%	0.00%

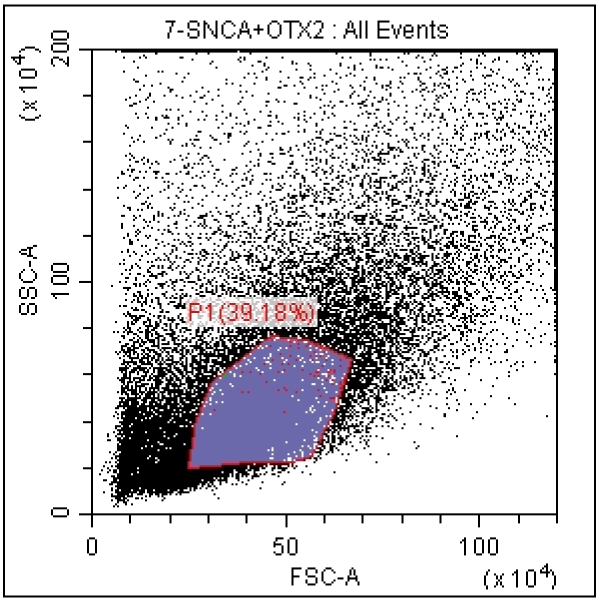

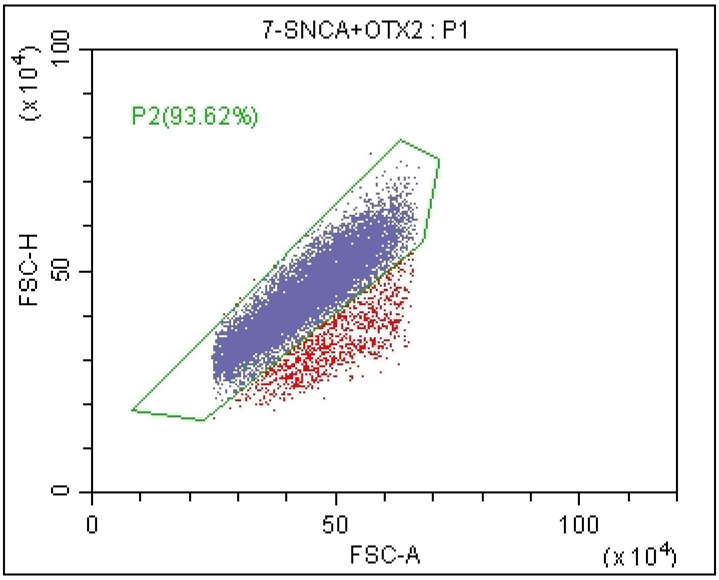

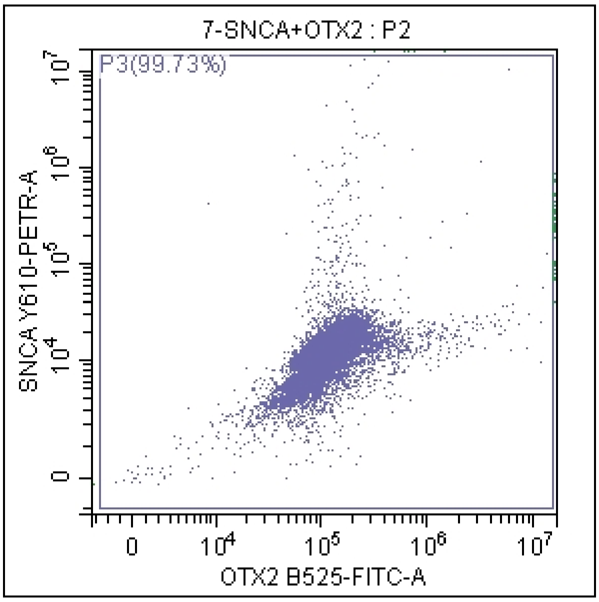

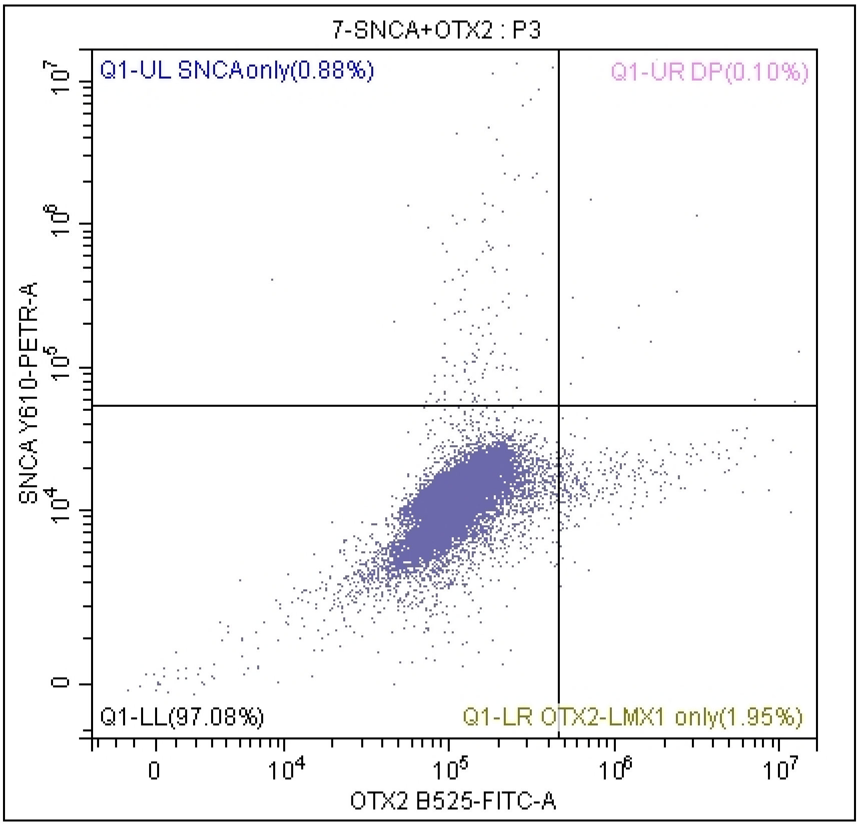

Tube Name: 7-SNCA+OTX2

Sample ID:

Population	Events	% Total	% Parent
All Events	40000	100.00%	100.00%
P1	15673	39.18%	39.18%
P2	14673	36.68%	93.62%
P3	14633	36.58%	99.73%
Q1-UR DP	14	0.04%	0.10%
Q1-UL SNCA only	129	0.32%	0.88%
Q1-LL	14205	35.51%	97.08%
Q1-LR OTX2-LMX1 only	285	0.71%	1.95%

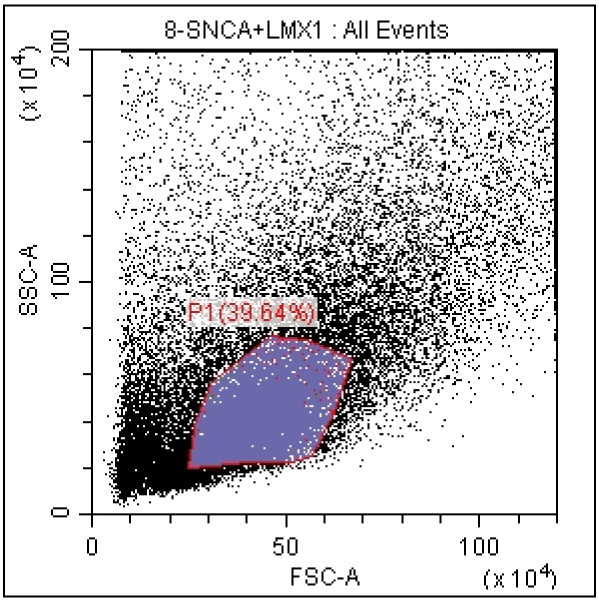

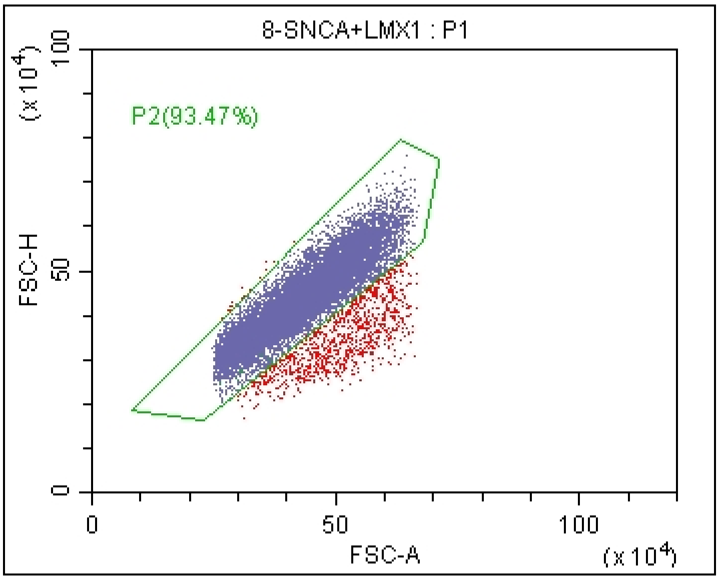

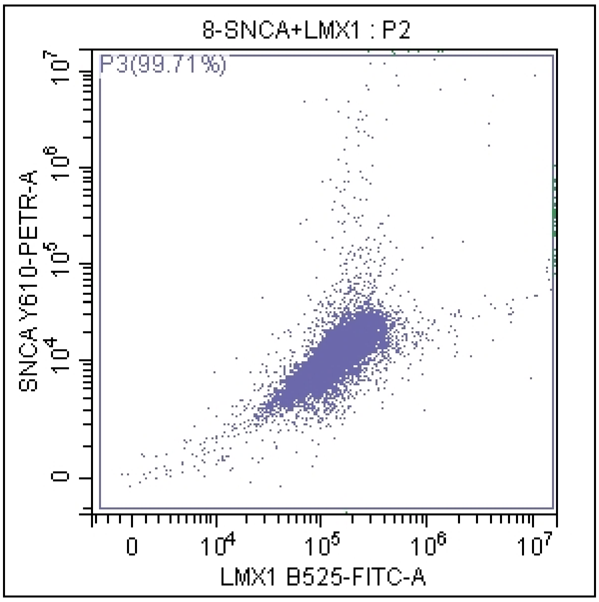

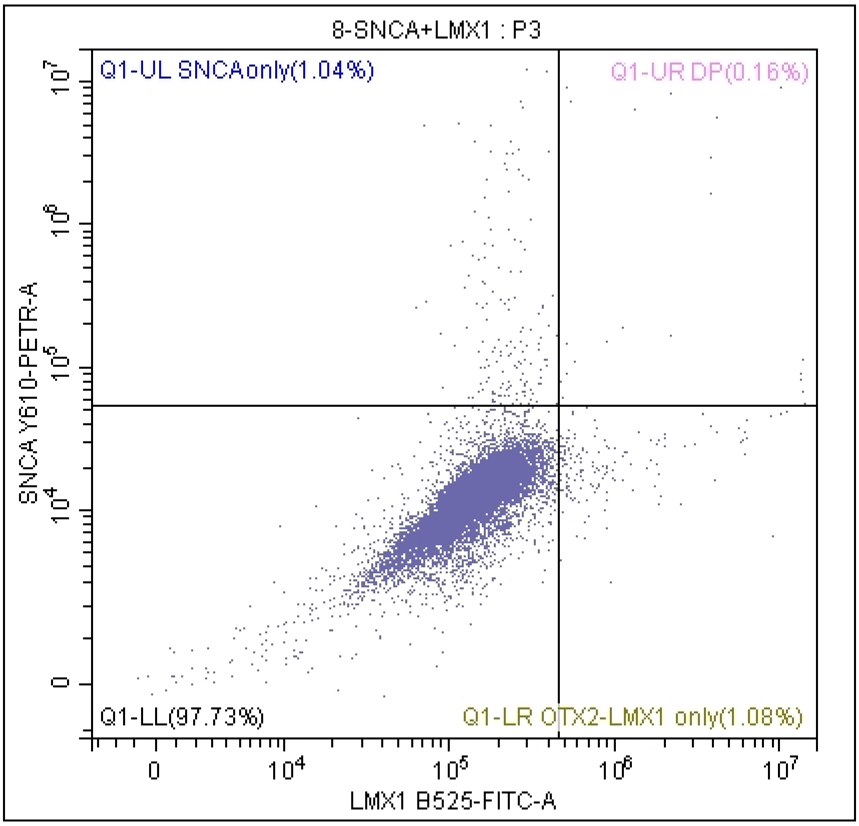

Tube Name: 8-SNCA+LMX1

Sample ID:

Population	Events	% Total	% Parent
All Events	40000	100.00%	100.00%
P1	15856	39.64%	39.64%
P2	14820	37.05%	93.47%
P3	14777	36.94%	99.71%
Q1-UR DP	23	0.06%	0.16%
Q1-UL SNCA only	153	0.38%	1.04%
Q1-LL	14441	36.10%	97.73%
Q1-LR OTX2-LMX1 only	160	0.40%	1.08%

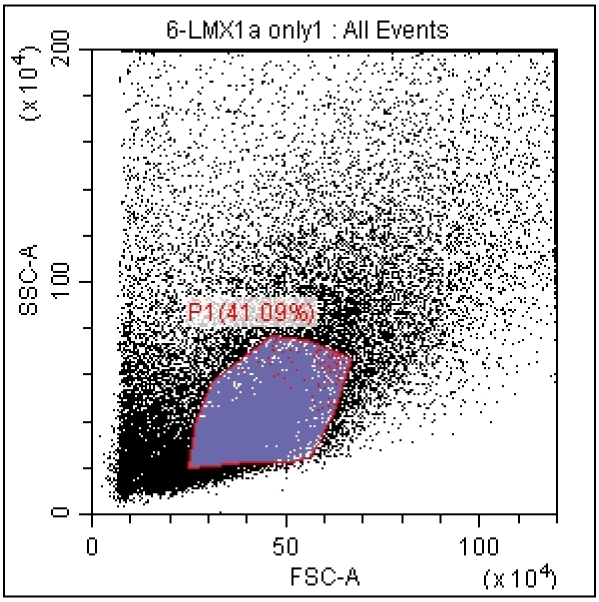

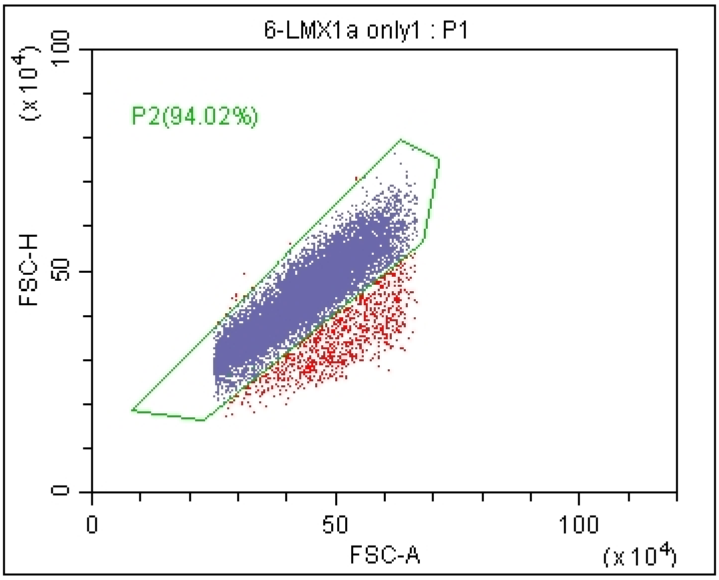

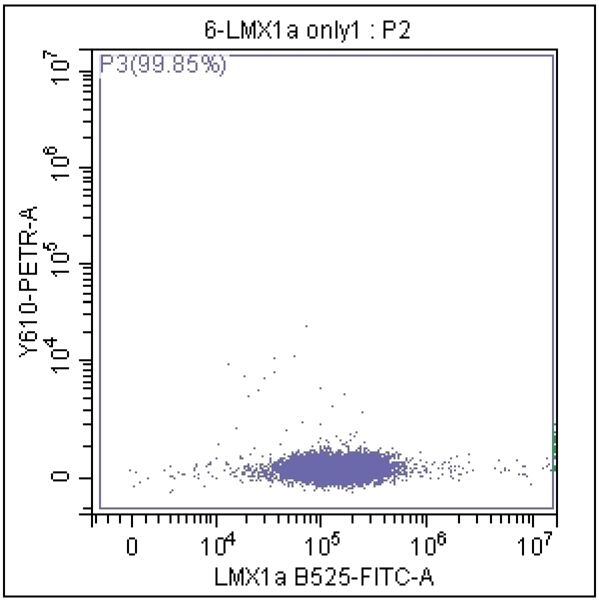

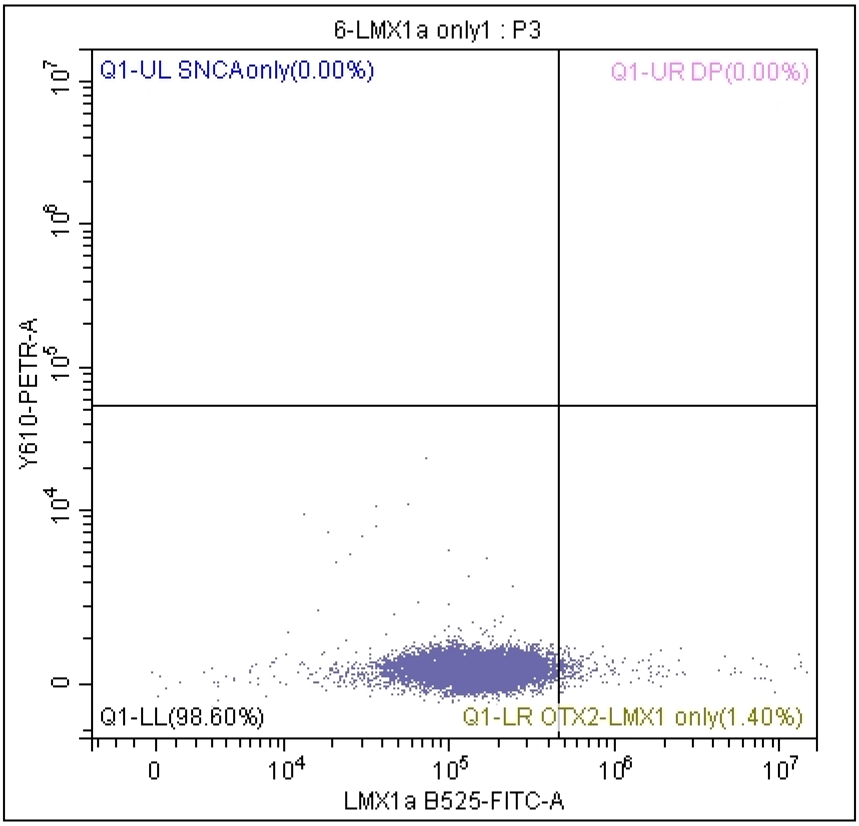

Tube Name: 6-LMX1a only1

Sample ID:

Population	Events	% Total	% Parent
All Events	40000	100.00%	100.00%
P1	16437	41.09%	41.09%
P2	15454	38.64%	94.02%
P3	15431	38.58%	99.85%
Q1-UR DP	0	0.00%	0.00%
Q1-UL SNCA only	0	0.00%	0.00%
Q1-LL	15215	38.04%	98.60%
Q1-LR OTX2-LMX1 only	216	0.54%	1.40%
