# Supplementary material for: Identification of an early subset of cerebellar nuclei neurons in mice
Source: eLife. 2024 Dec 16;13:RP93778. doi: 10.7554/eLife.93778 (PMC11649241; doi:10.7554/eLife.93778)
Supplement: Figure 3—source data 2. [file elife-93778-fig3-data2.zip › 190807 Maryam Celebellum flow.pdf]

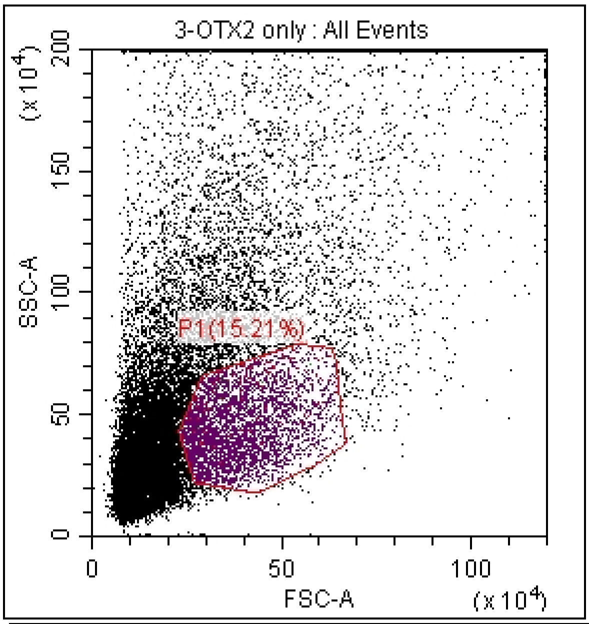

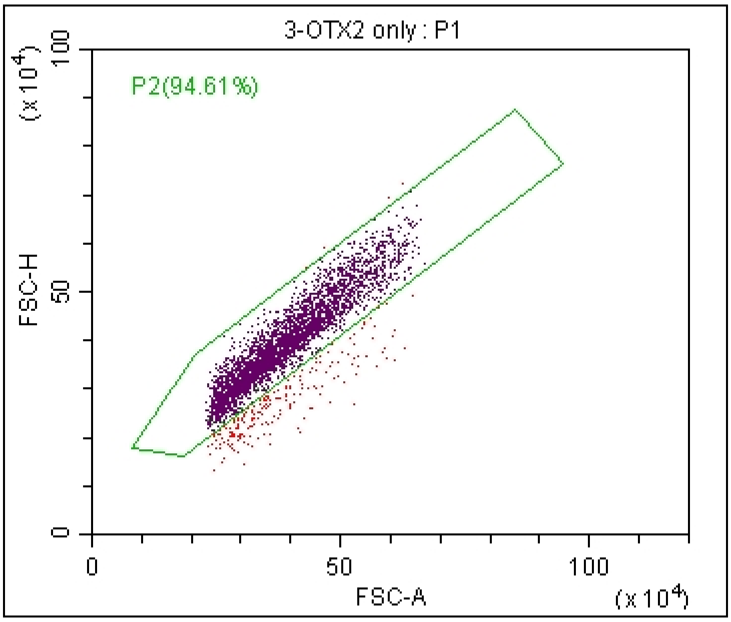

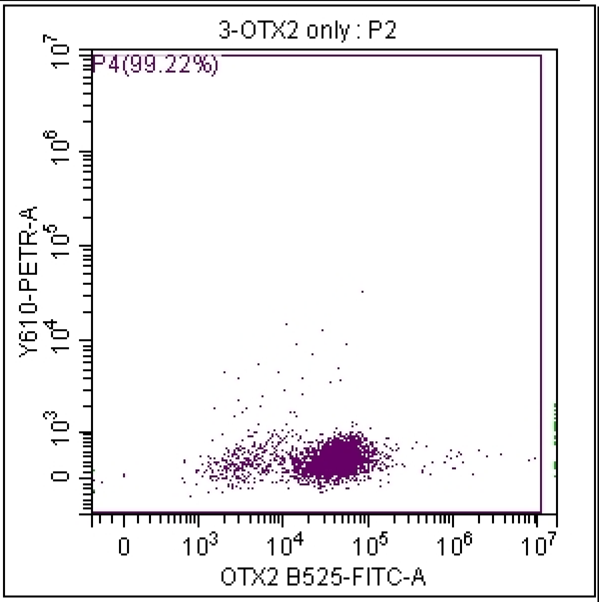

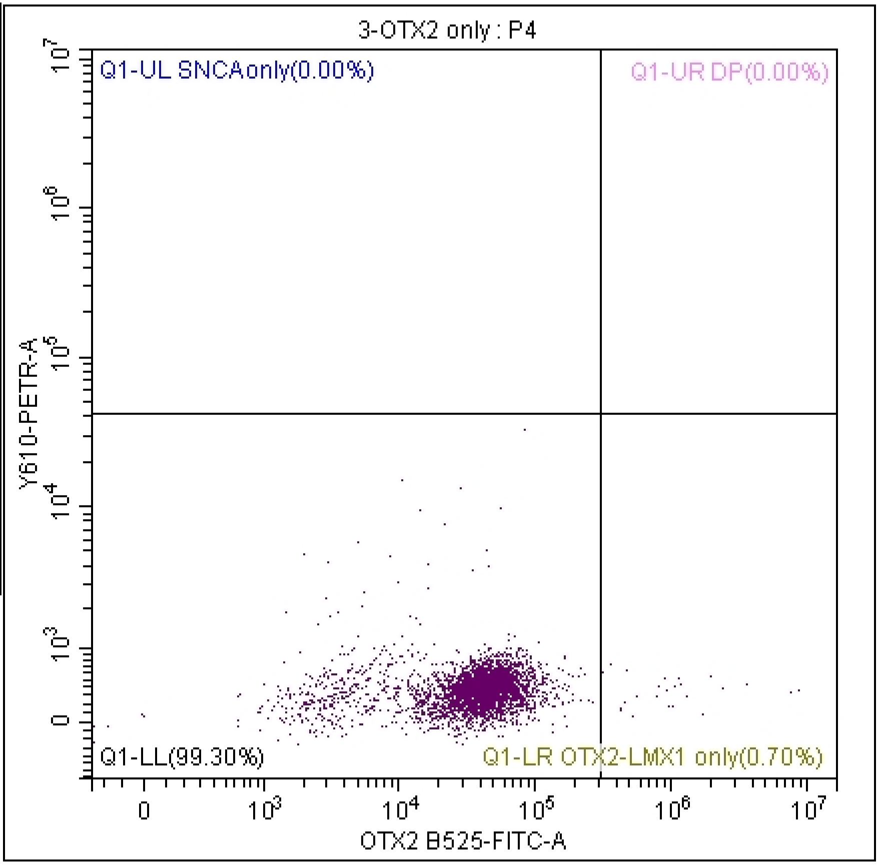

Tube Name: 3-OTX2 only

Sample ID:

Population	Events	% Total	% Parent
All Events	25000	100.00%	100.00%
P1	3802	15.21%	15.21%
P2	3597	14.39%	94.61%
P4	3569	14.28%	99.22%
Q1-UR DP	0	0.00%	0.00%
Q1-UL SNCA only	0	0.00%	0.00%
Q1-LL	3544	14.18%	99.30%
Q1-LR OTX2-LMX1 only	25	0.10%	0.70%

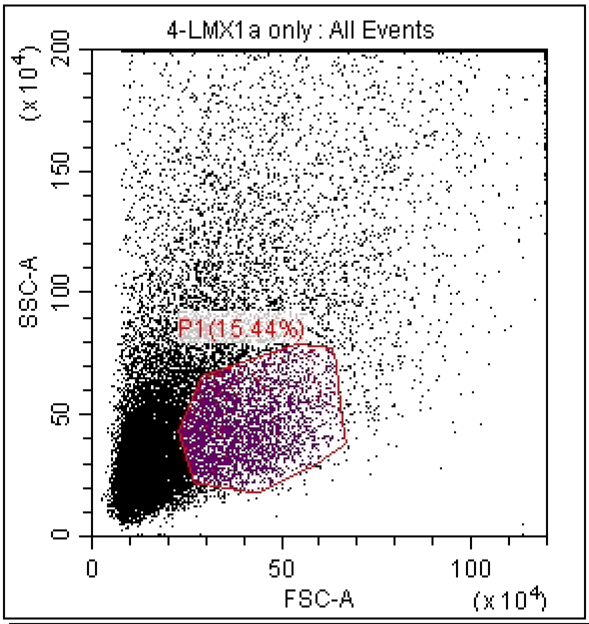

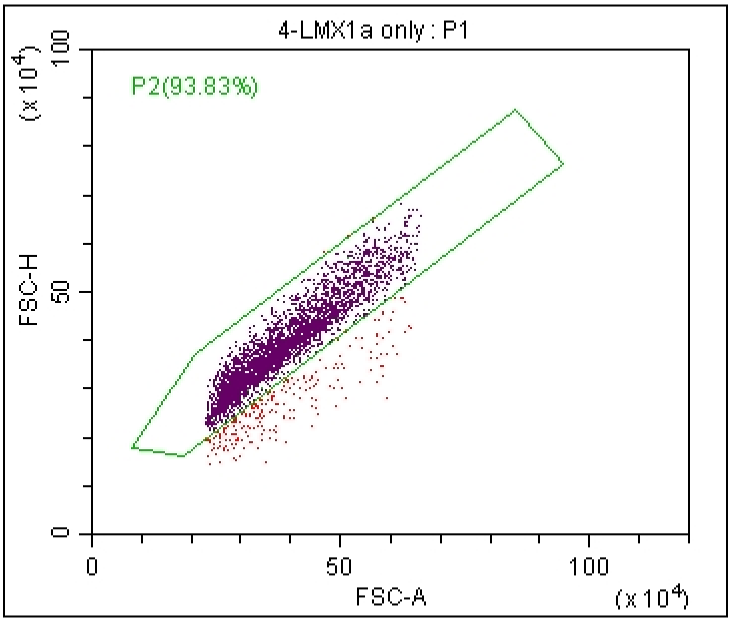

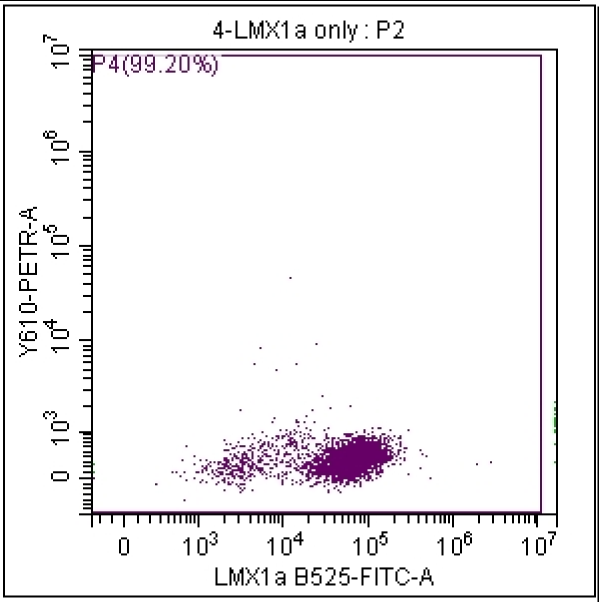

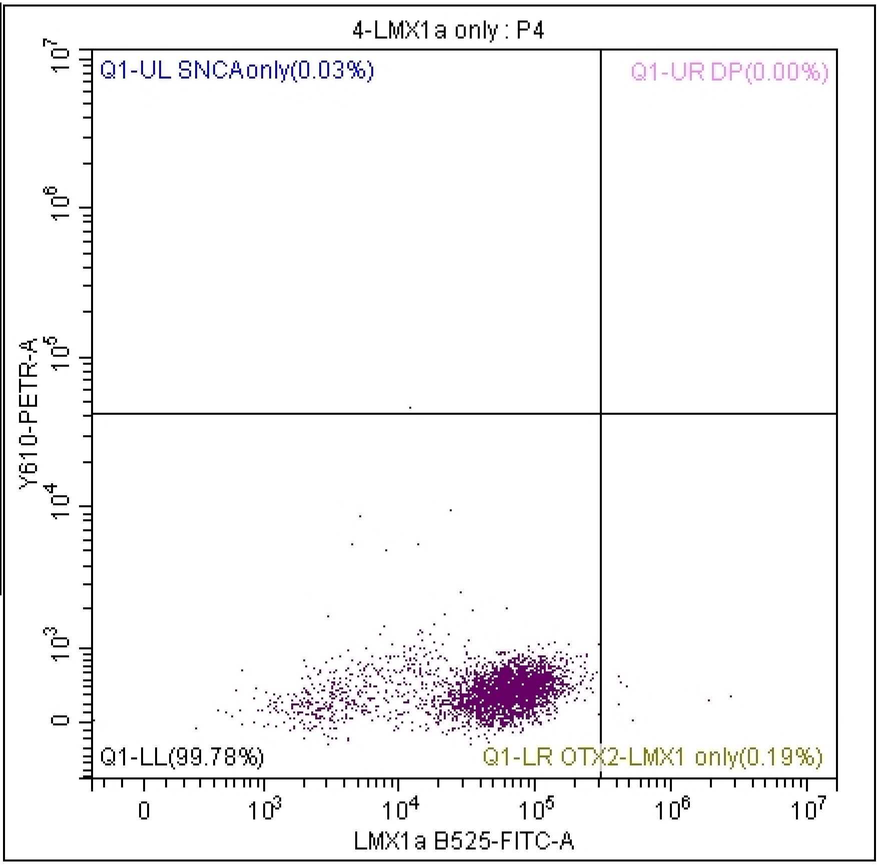

Tube Name: 4-LMX1a only

Sample ID:

Population	Events	% Total	% Parent
All Events	25000	100.00%	100.00%
P1	3859	15.44%	15.44%
P2	3621	14.48%	93.83%
P4	3592	14.37%	99.20%
Q1-UR DP	0	0.00%	0.00%
Q1-UL SNCA only	1	0.00%	0.03%
Q1-LL	3584	14.34%	99.78%
Q1-LR OTX2-LMX1 only	7	0.03%	0.19%

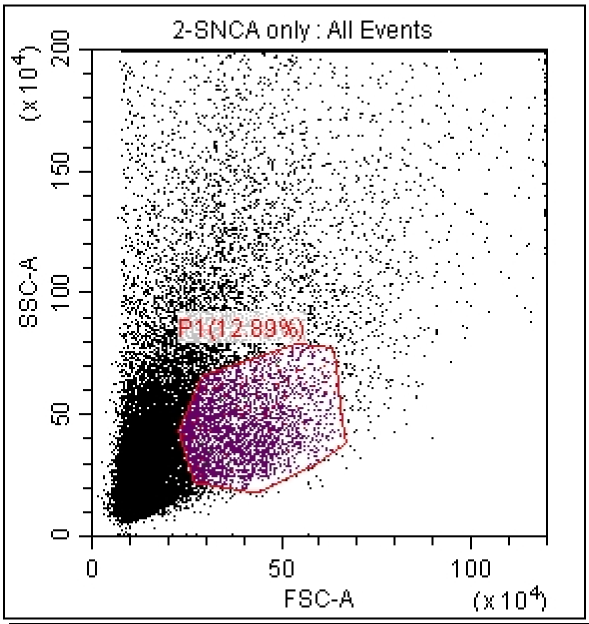

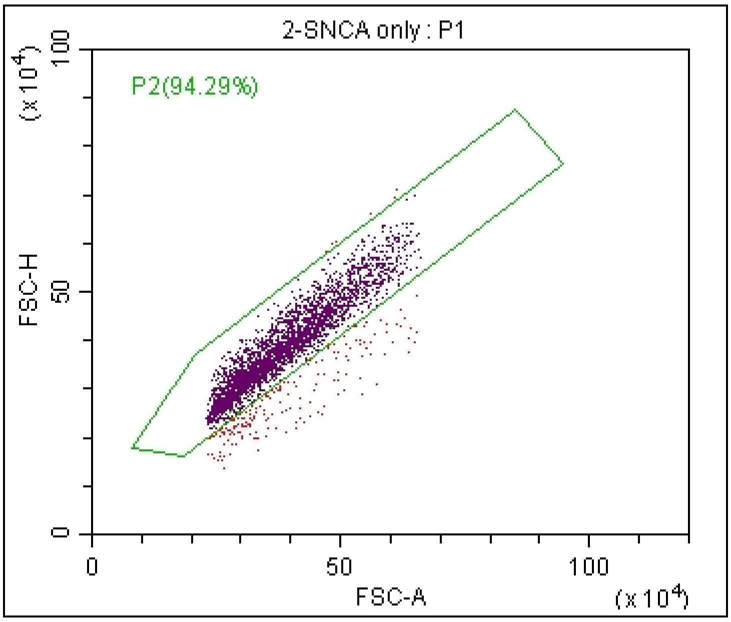

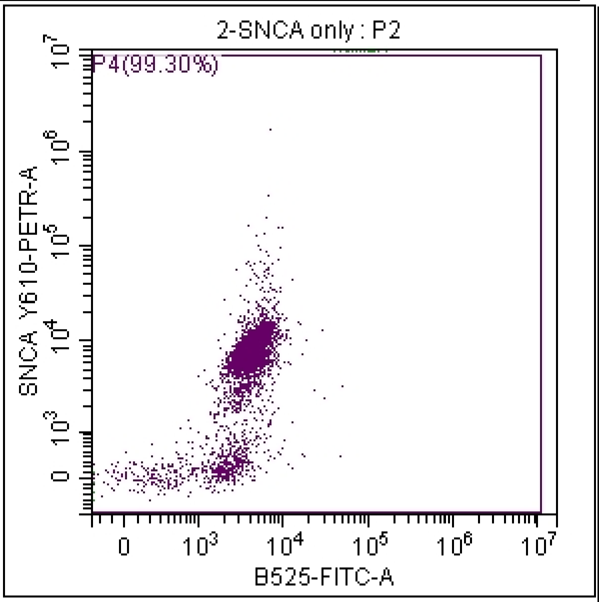

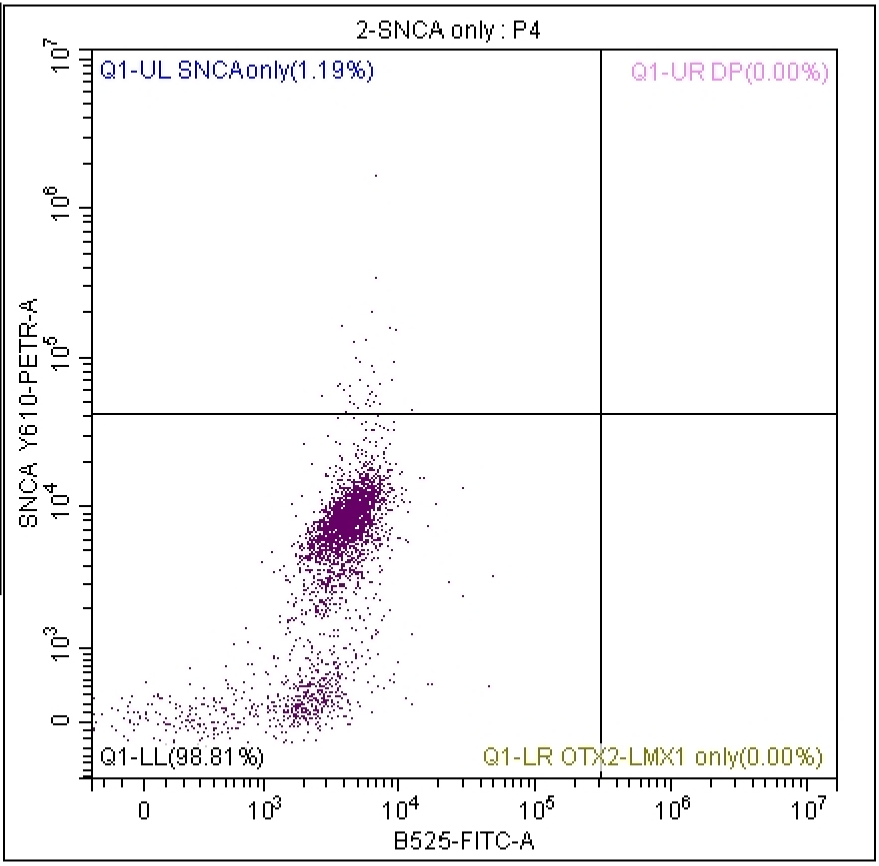

Tube Name: 2-SNCA only

Sample ID:

Population	Events	% Total	% Parent
All Events	25803	100.00%	100.00%
P1	3327	12.89%	12.89%
P2	3137	12.16%	94.29%
P4	3115	12.07%	99.30%
Q1-UR DP	0	0.00%	0.00%
Q1-UL SNCA only	37	0.14%	1.19%
Q1-LL	3078	11.93%	98.81%
Q1-LR OTX2-LMX1 only	0	0.00%	0.00%

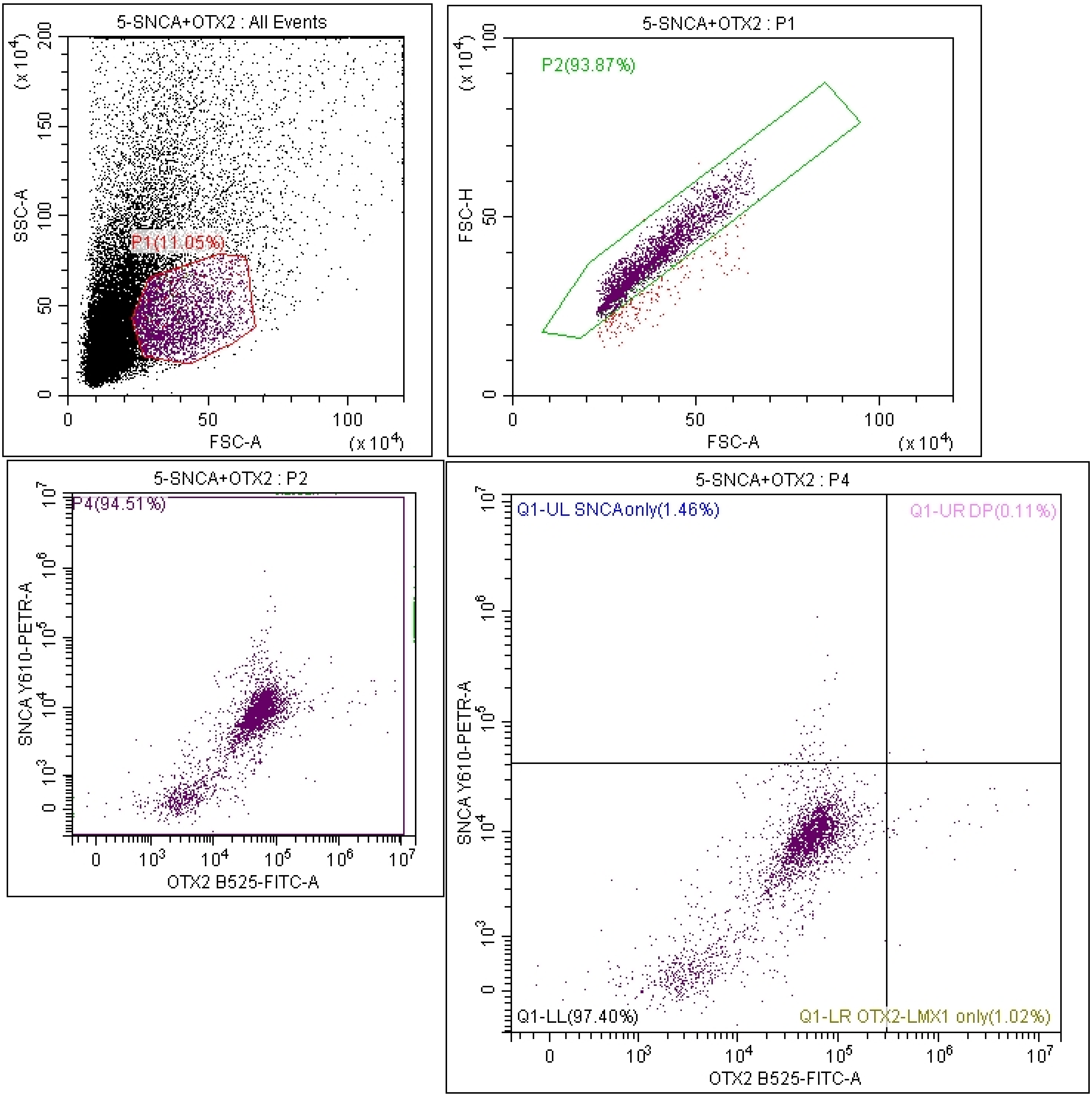

Tube Name: 5-SNCA+OTX2

Sample ID:

Population	Events	% Total	% Parent
All Events	27888	100.00%	100.00%
P1	3083	11.05%	11.05%
P2	2894	10.38%	93.87%
P4	2735	9.81%	94.51%
Q1-UR DP	3	0.01%	0.11%
Q1-UL SNCA only	40	0.14%	1.46%
Q1-LL	2664	9.55%	97.40%
Q1-LR OTX2-LMX1 only	28	0.10%	1.02%

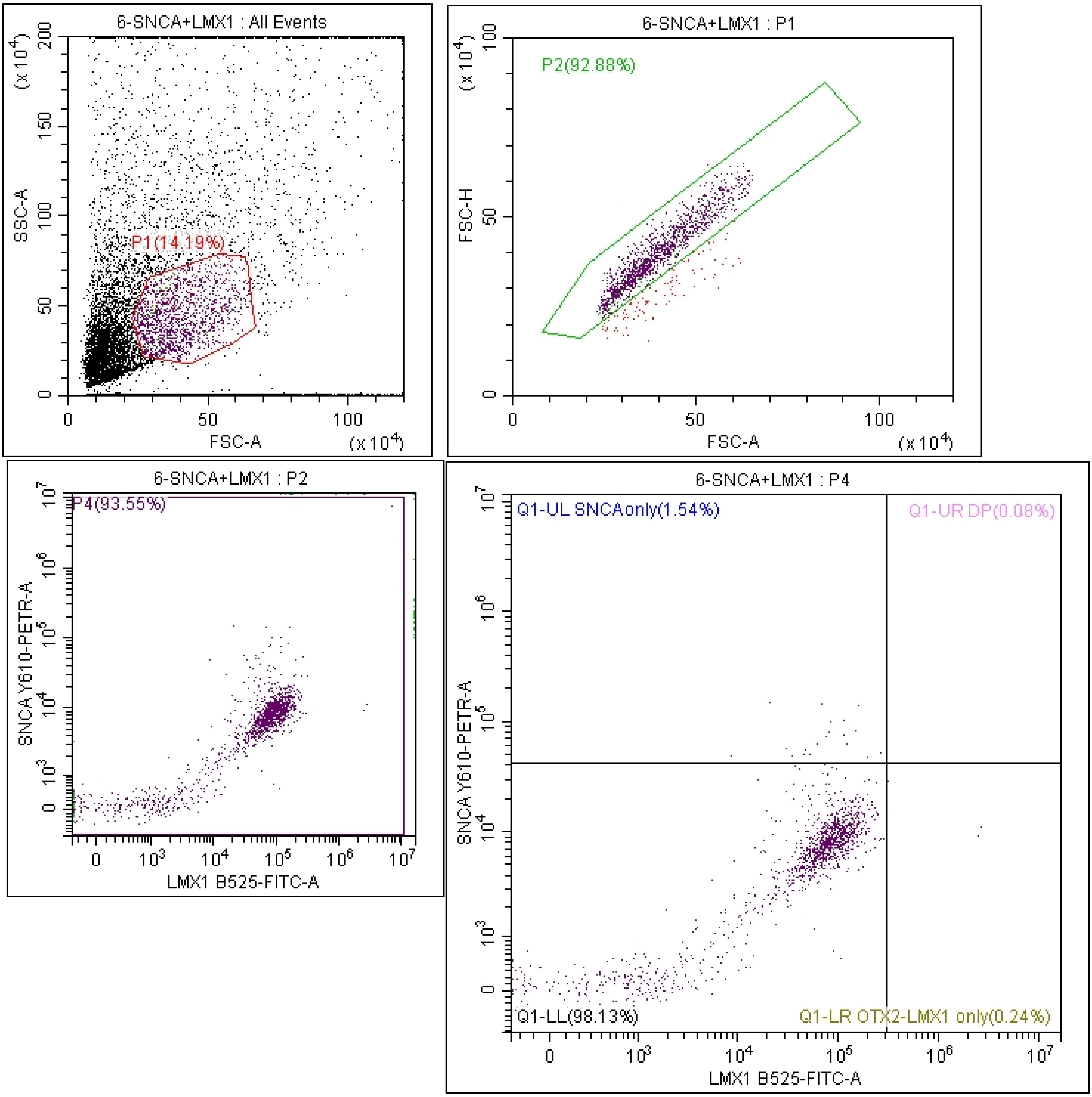

Tube Name: 6-SNCA+LMX1

Sample ID:

Population	Events	% Total	% Parent
All Events	10000	100.00%	100.00%
P1	1419	14.19%	14.19%
P2	1318	13.18%	92.88%
P4	1233	12.33%	93.55%
Q1-UR DP	1	0.01%	0.08%
Q1-UL SNCA only	19	0.19%	1.54%
Q1-LL	1210	12.10%	98.13%
Q1-LR OTX2-LMX1 only	3	0.03%	0.24%

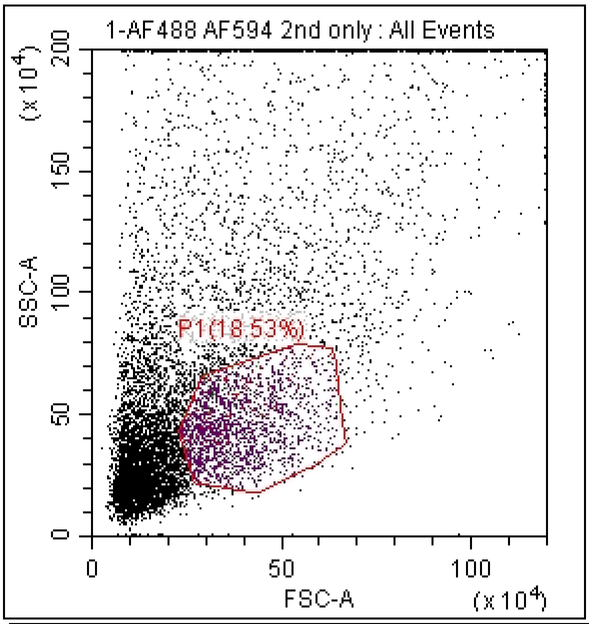

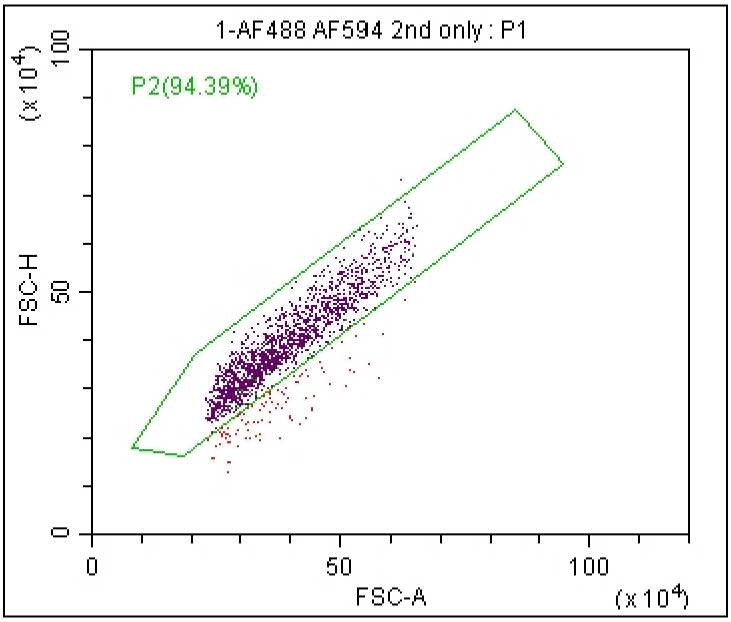

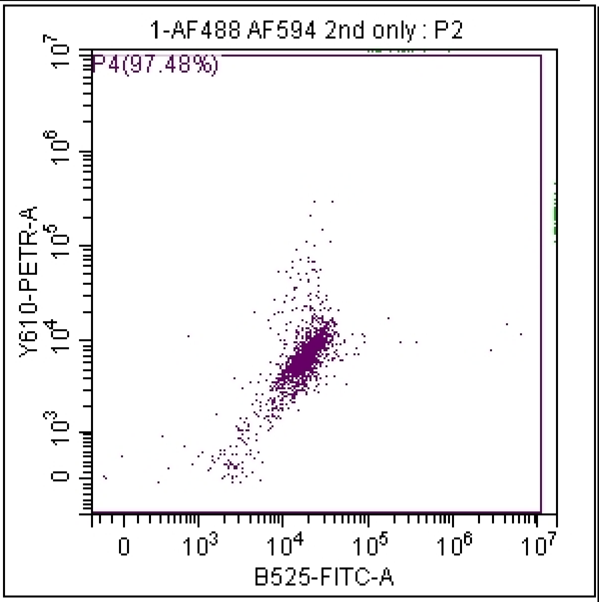

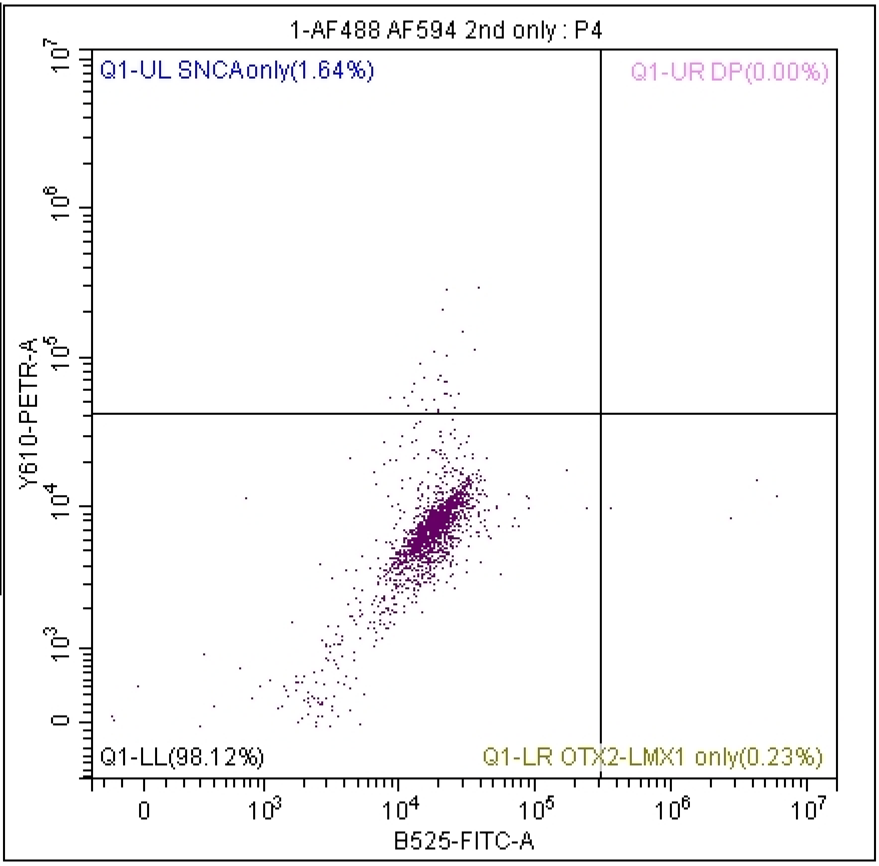

Tube Name: 1-AF488 AF594 2nd only

Sample ID:

Population	Events	% Total	% Parent
All Events	10000	100.00%	100.00%
P1	1853	18.53%	18.53%
P2	1749	17.49%	94.39%
P4	1705	17.05%	97.48%
Q1-UR DP	0	0.00%	0.00%
Q1-UL SNCA only	28	0.28%	1.64%
Q1-LL	1673	16.73%	98.12%
Q1-LR OTX2-LMX1 only	4	0.04%	0.23%

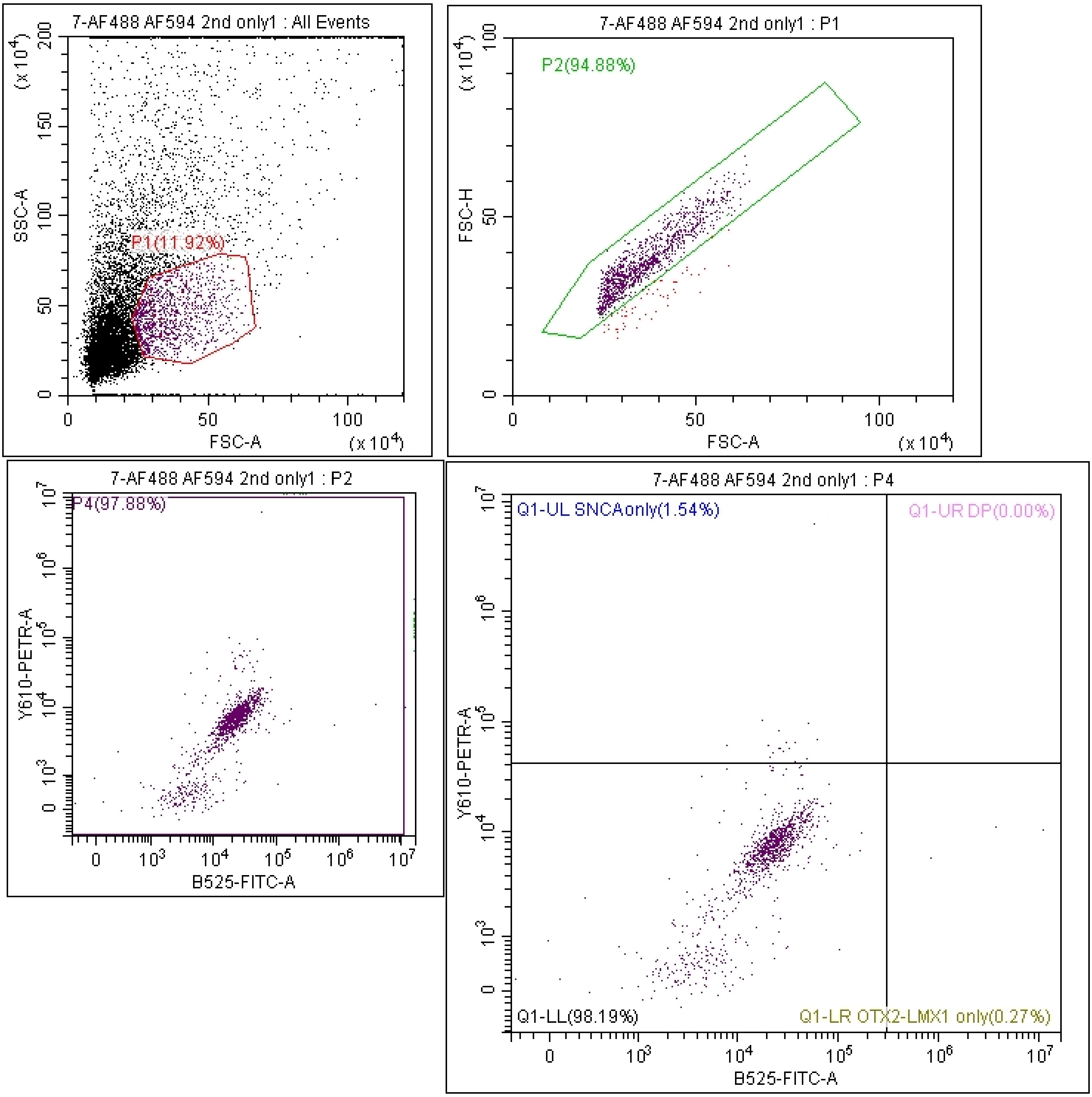

Tube Name: 7-AF488 AF594 2nd only1
Sample ID:

Population	Events	% Total	% Parent
All Events	10000	100.00%	100.00%
P1	1192	11.92%	11.92%
P2	1131	11.31%	94.88%
P4	1107	11.07%	97.88%
Q1-UR DP	0	0.00%	0.00%
Q1-UL SNCA only	17	0.17%	1.54%
Q1-LL	1087	10.87%	98.19%
Q1-LR OTX2-LMX1 only	3	0.03%	0.27%

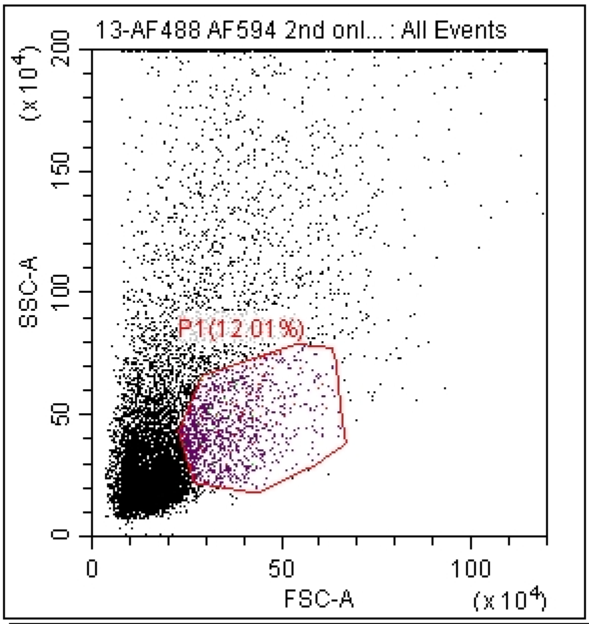

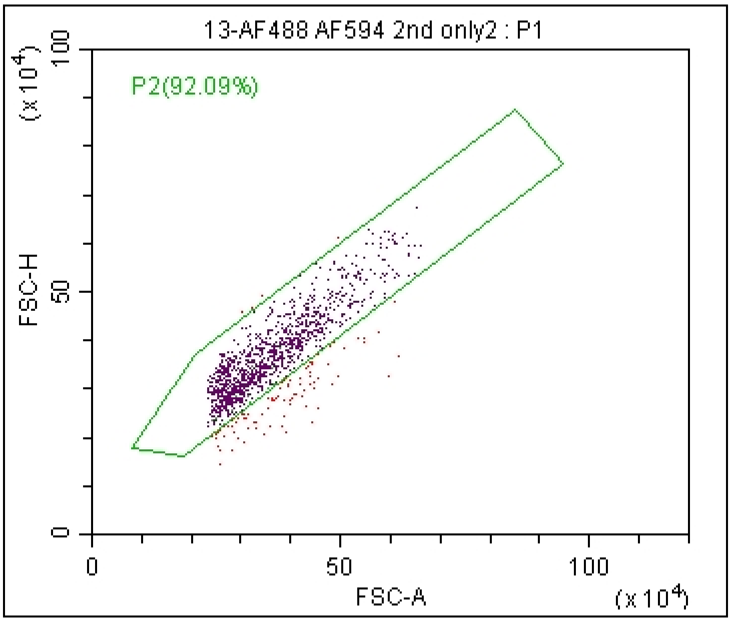

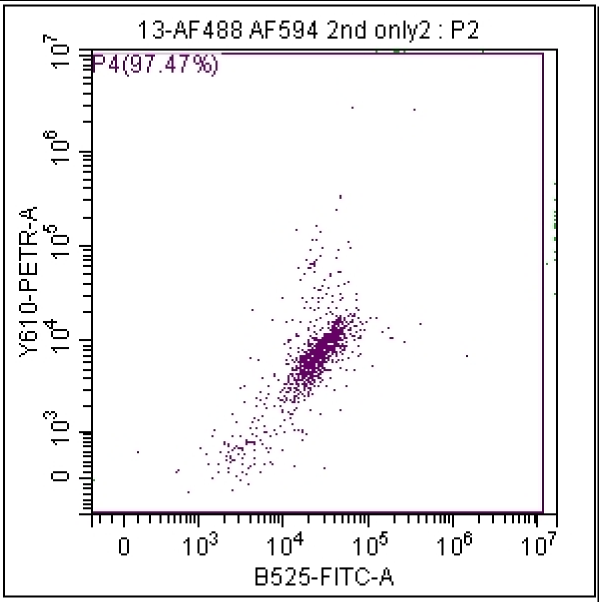

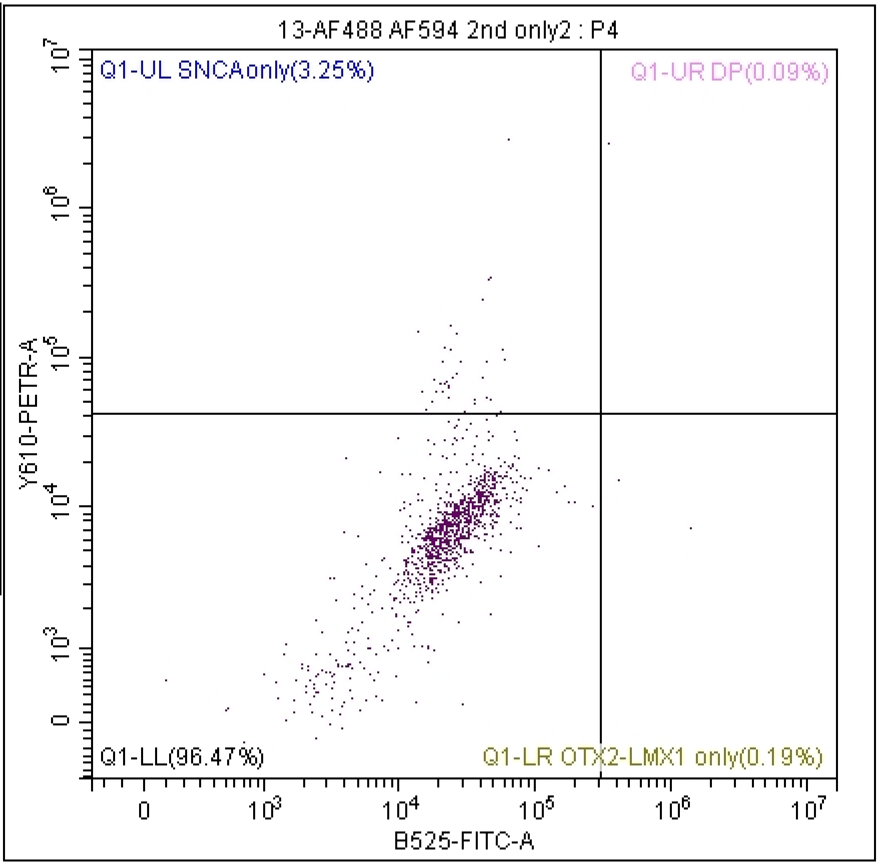

Tube Name: 13-AF488 AF594 2nd only2

Sample ID:

Population	Events	% Total	% Parent
All Events	10000	100.00%	100.00%
P1	1201	12.01%	12.01%
P2	1106	11.06%	92.09%
P4	1078	10.78%	97.47%
Q1-UR DP	1	0.01%	0.09%
Q1-UL SNCA only	35	0.35%	3.25%
Q1-LL	1040	10.40%	96.47%
Q1-LR OTX2-LMX1 only	2	0.02%	0.19%

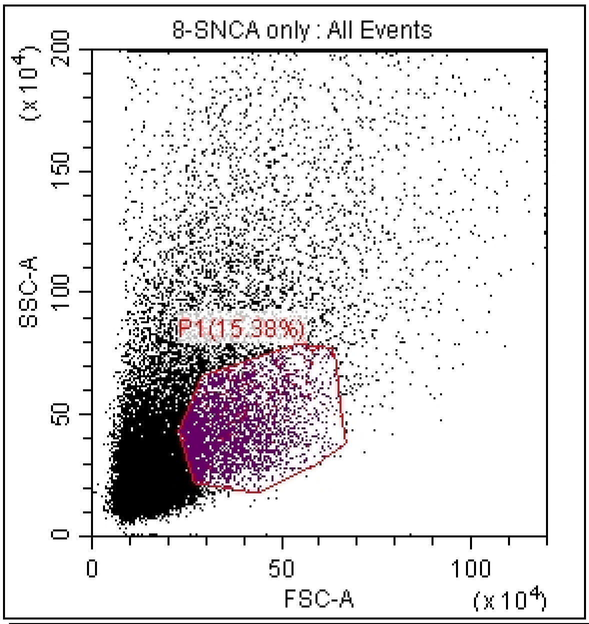

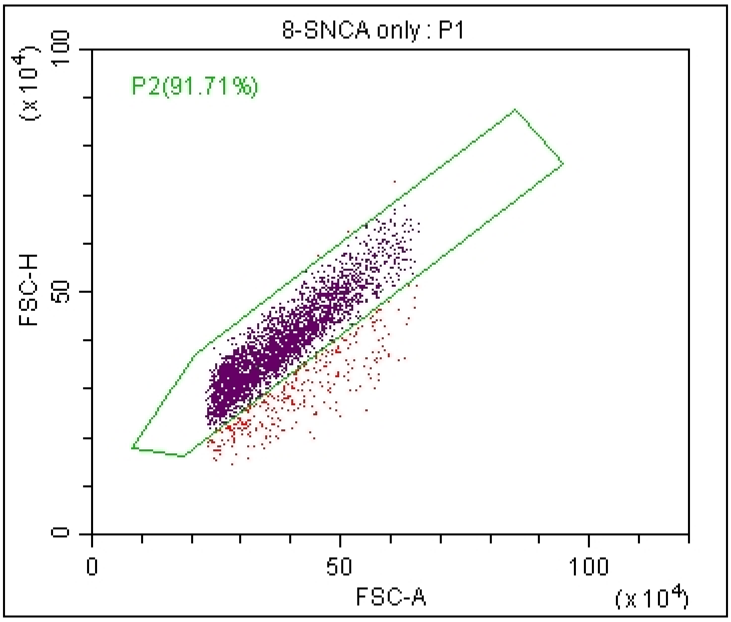

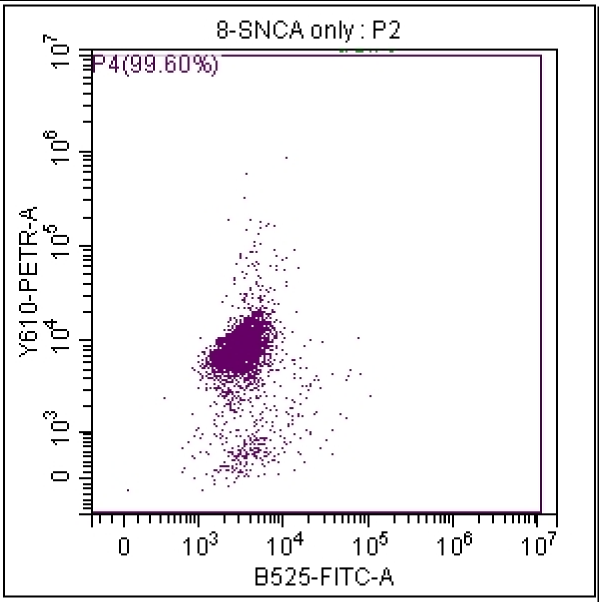

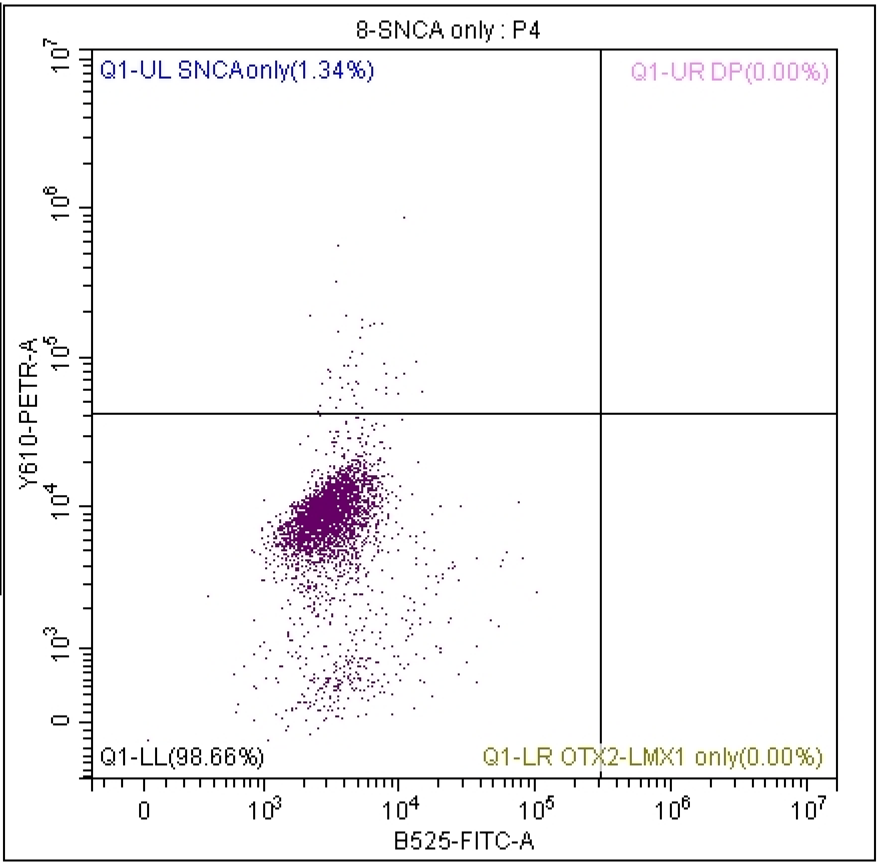

Tube Name: 8-SNCA only

Sample ID:

Population	Events	% Total	% Parent
All Events	25000	100.00%	100.00%
P1	3846	15.38%	15.38%
P2	3527	14.11%	91.71%
P4	3513	14.05%	99.60%
Q1-UR DP	0	0.00%	0.00%
Q1-UL SNCA only	47	0.19%	1.34%
Q1-LL	3466	13.86%	98.66%
Q1-LR OTX2-LMX1 only	0	0.00%	0.00%

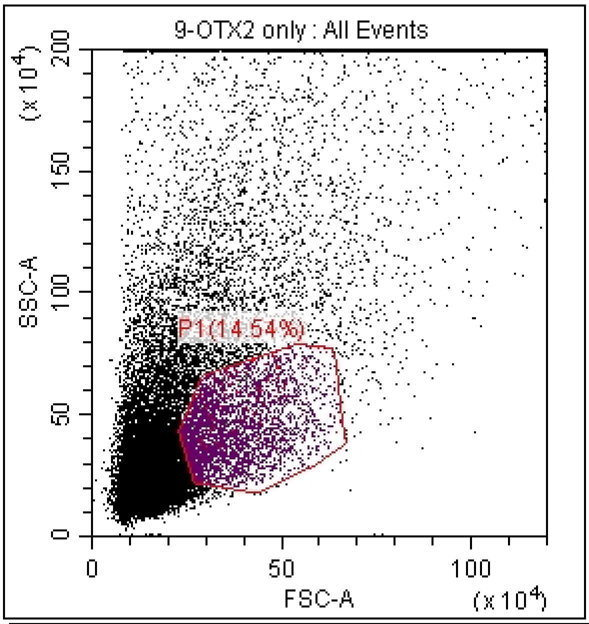

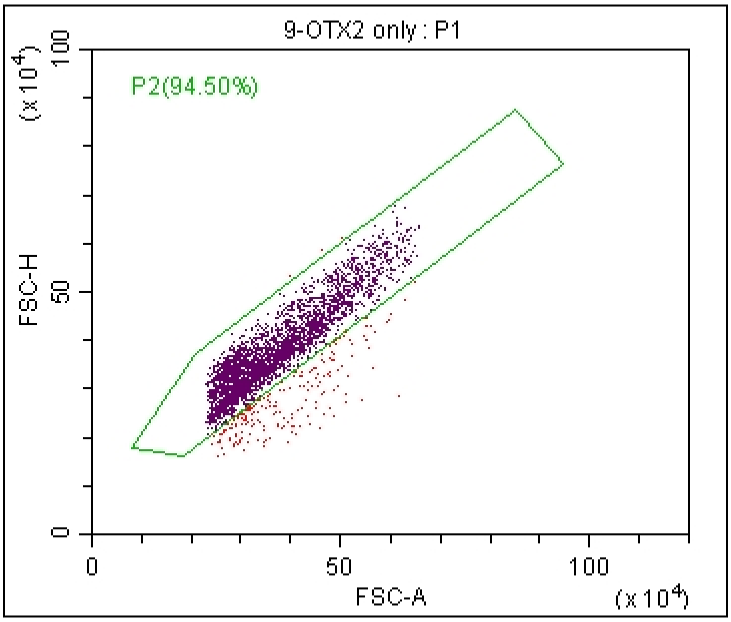

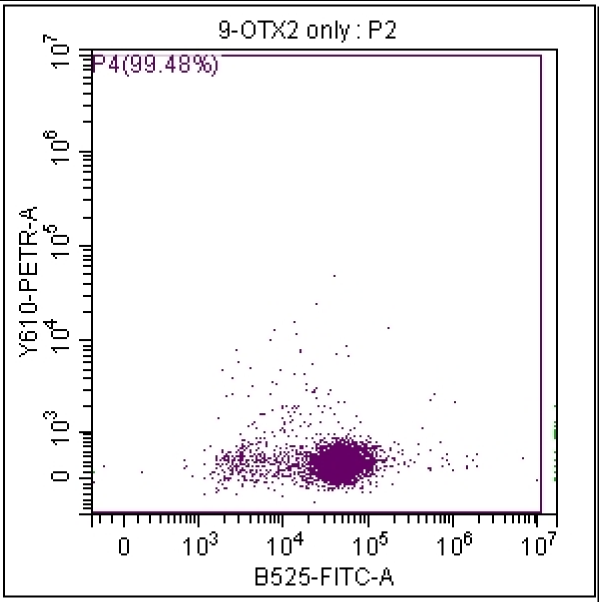

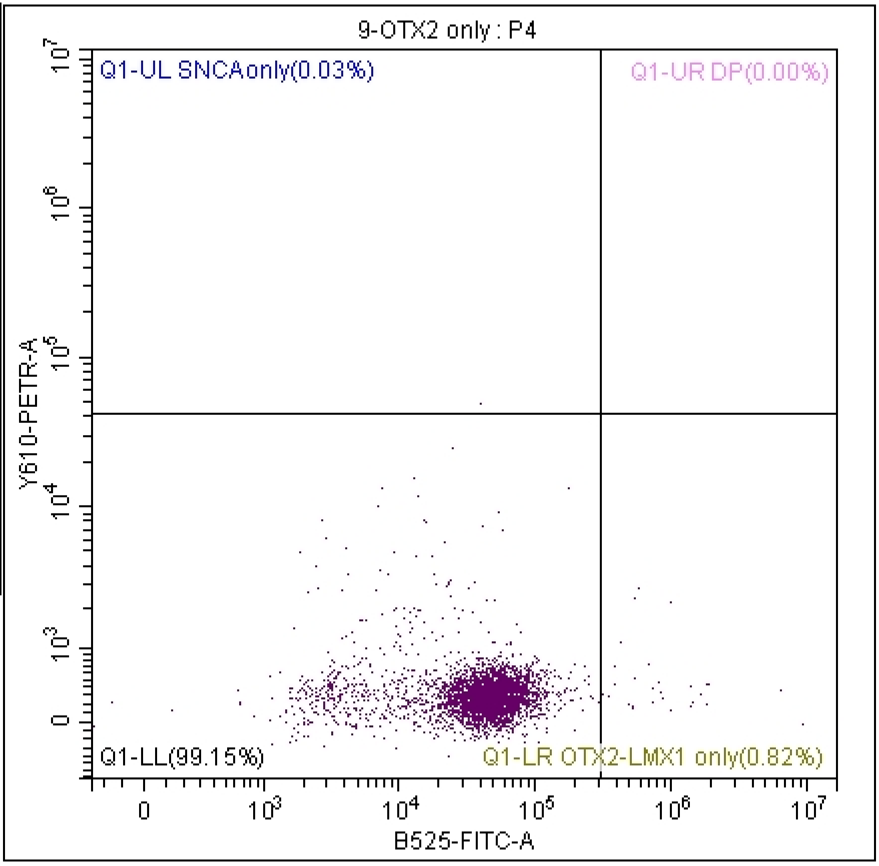

Tube Name: 9-OTX2 only

Sample ID:

Population	Events	% Total	% Parent
All Events	25000	100.00%	100.00%
P1	3635	14.54%	14.54%
P2	3435	13.74%	94.50%
P4	3417	13.67%	99.48%
Q1-UR DP	0	0.00%	0.00%
Q1-UL SNCA only	1	0.00%	0.03%
Q1-LL	3388	13.55%	99.15%
Q1-LR OTX2-LMX1 only	28	0.11%	0.82%

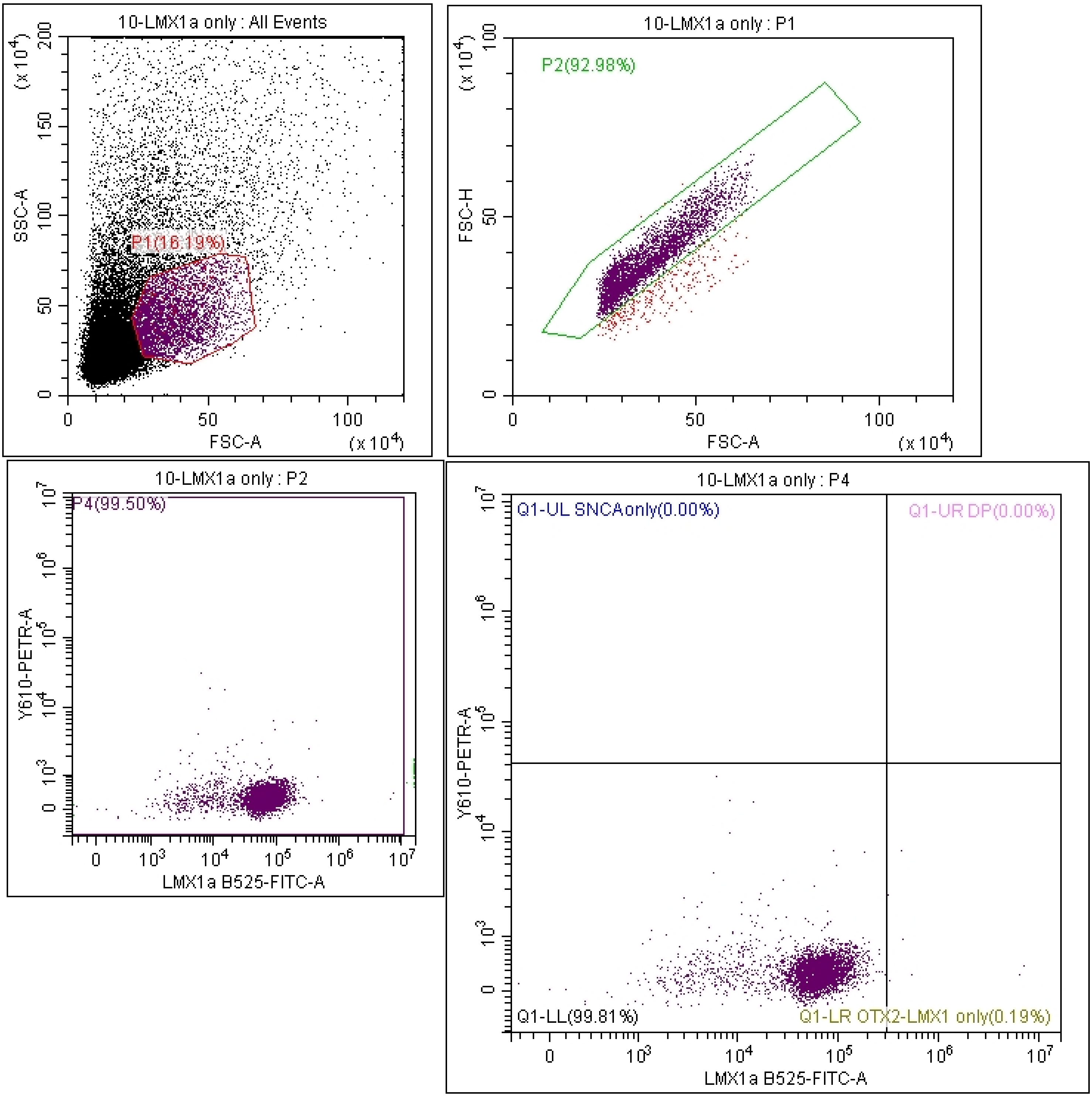

Tube Name: 10-LMX1a only

Sample ID:

Population	Events	% Total	% Parent
All Events	25000	100.00%	100.00%
P1	4047	16.19%	16.19%
P2	3763	15.05%	92.98%
P4	3744	14.98%	99.50%
Q1-UR DP	0	0.00%	0.00%
Q1-UL SNCA only	0	0.00%	0.00%
Q1-LL	3737	14.95%	99.81%
Q1-LR OTX2-LMX1 only	7	0.03%	0.19%

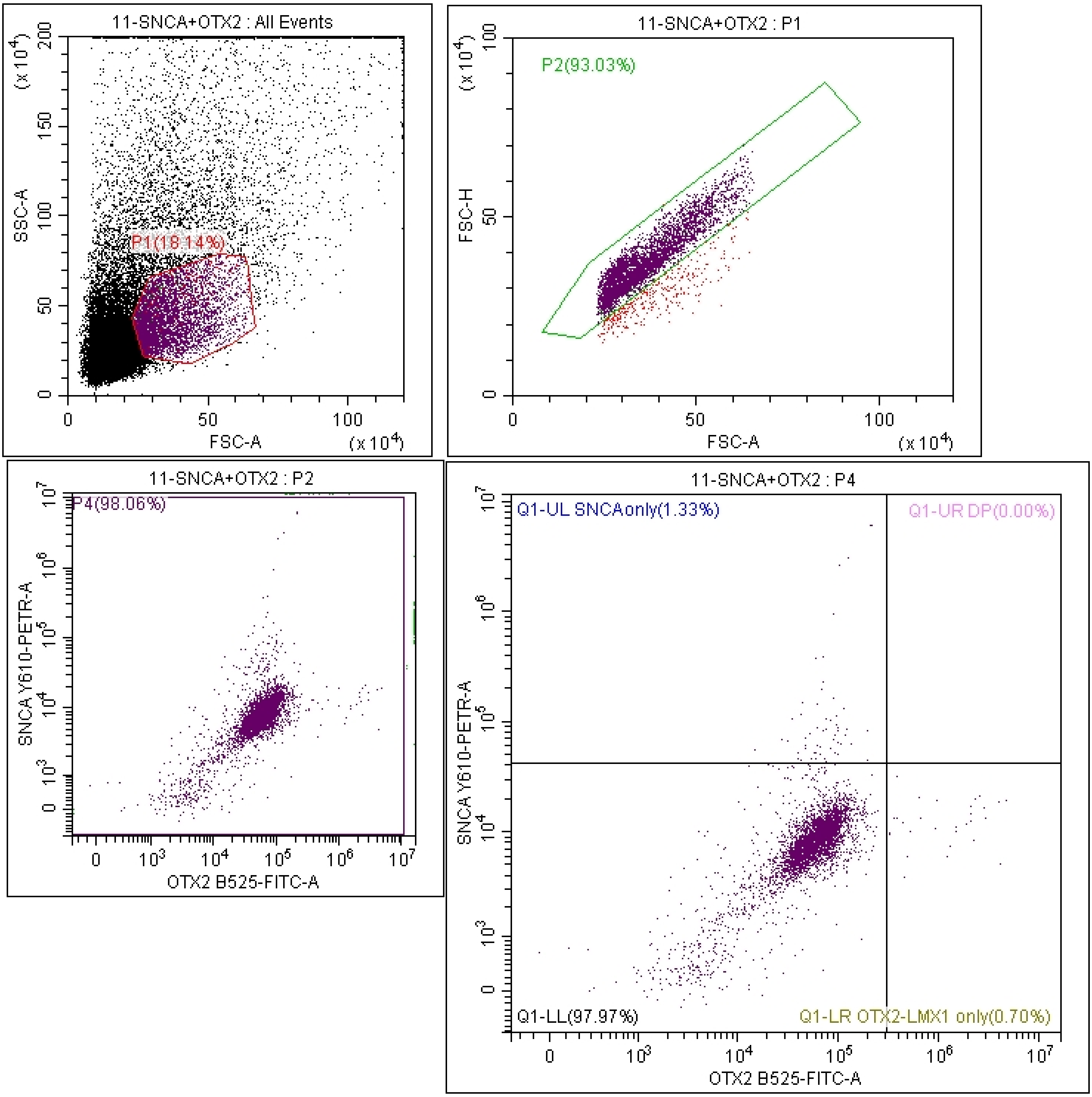

Tube Name: 11-SNCA+OTX2

Sample ID:

Population	Events	% Total	% Parent
All Events	25000	100.00%	100.00%
P1	4535	18.14%	18.14%
P2	4219	16.88%	93.03%
P4	4137	16.55%	98.06%
Q1-UR DP	0	0.00%	0.00%
Q1-UL SNCA only	55	0.22%	1.33%
Q1-LL	4053	16.21%	97.97%
Q1-LR OTX2-LMX1 only	29	0.12%	0.70%

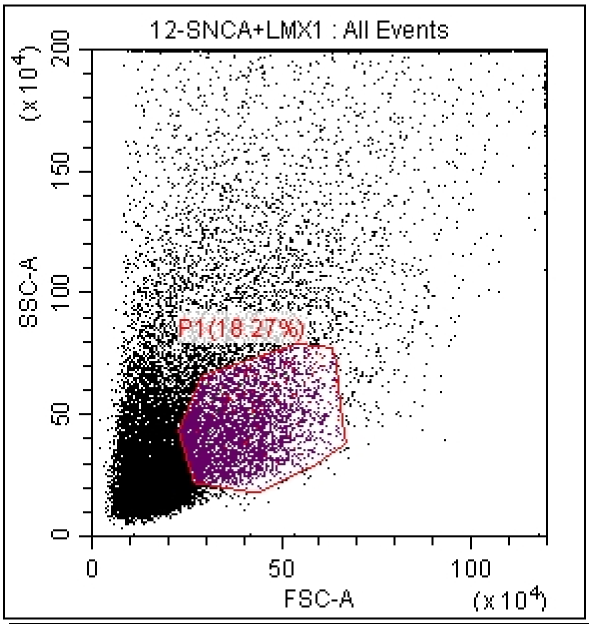

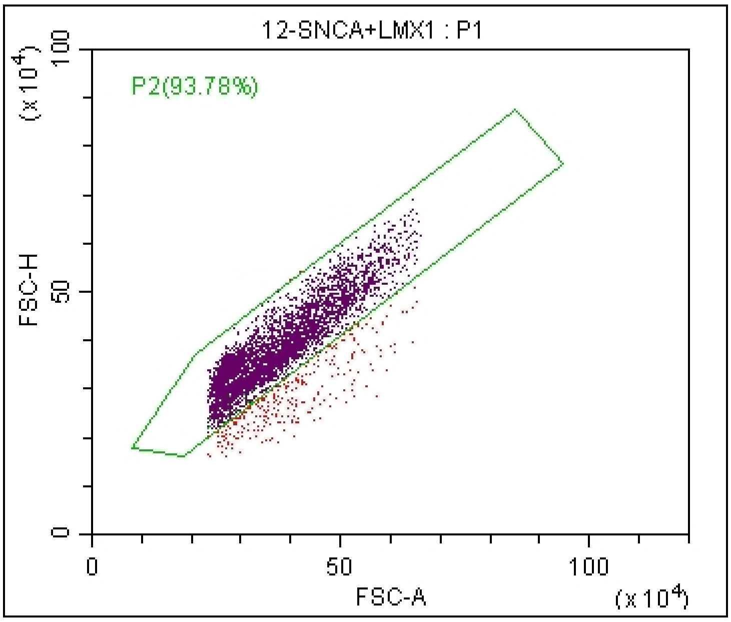

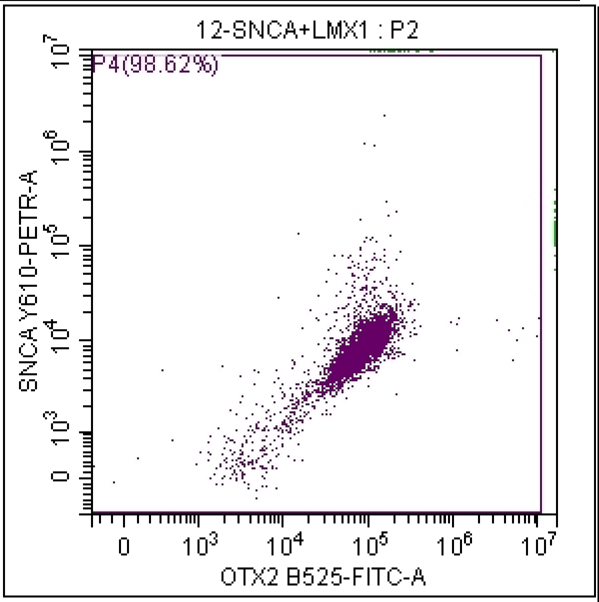

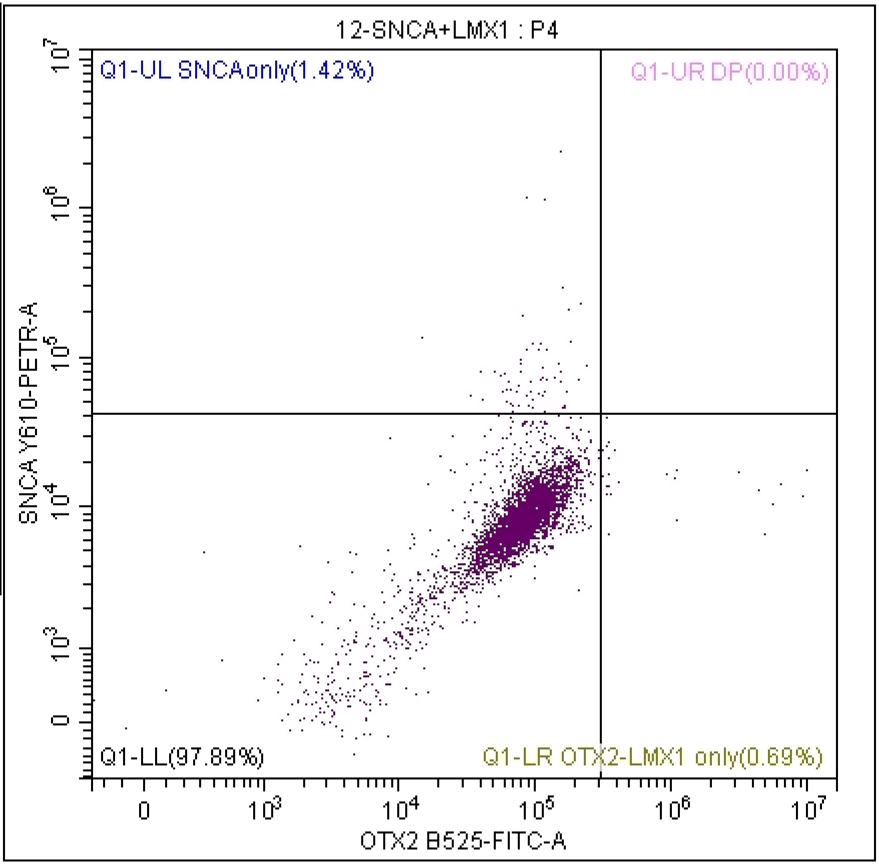

Tube Name: 12-SNCA+LMX1

Sample ID:

Population	Events	% Total	% Parent
All Events	25000	100.00%	100.00%
P1	4567	18.27%	18.27%
P2	4283	17.13%	93.78%
P4	4224	16.90%	98.62%
Q1-UR DP	0	0.00%	0.00%
Q1-UL SNCA only	60	0.24%	1.42%
Q1-LL	4135	16.54%	97.89%
Q1-LR OTX2-LMX1 only	29	0.12%	0.69%

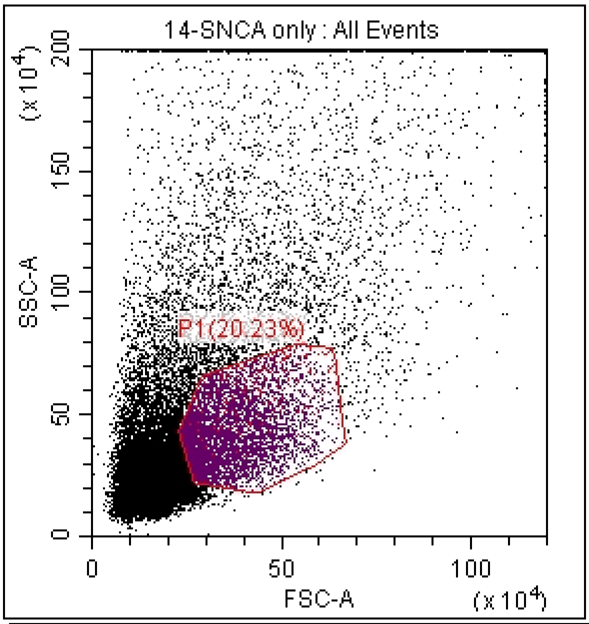

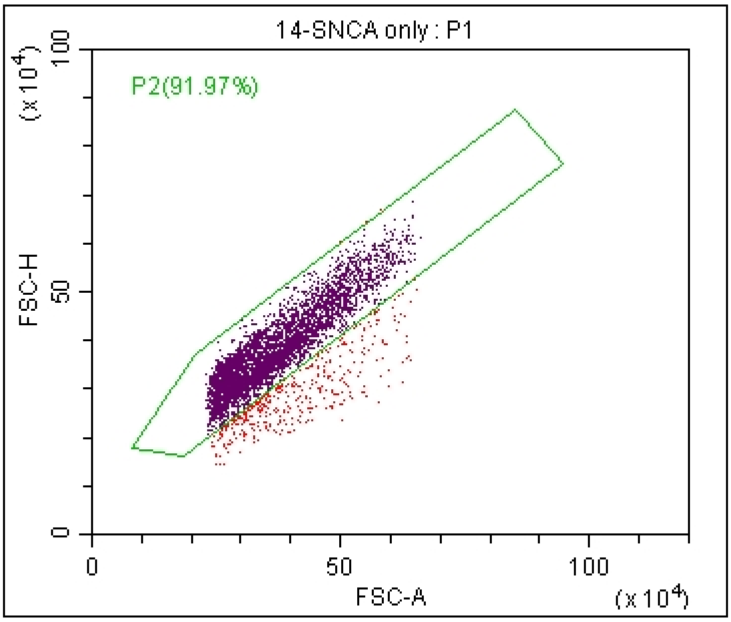

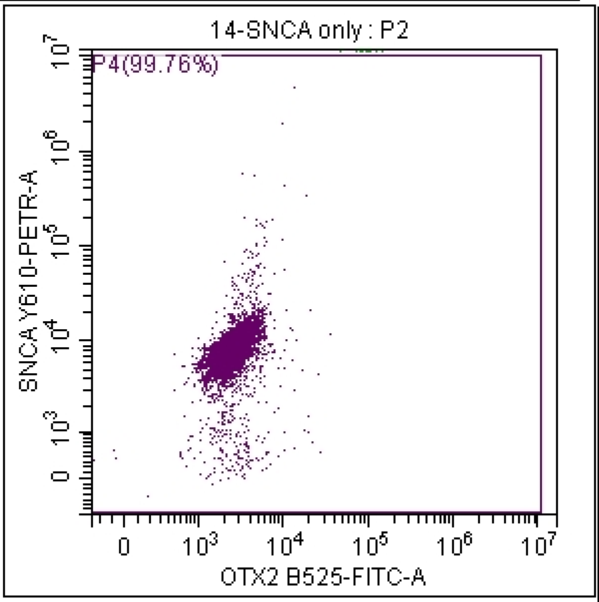

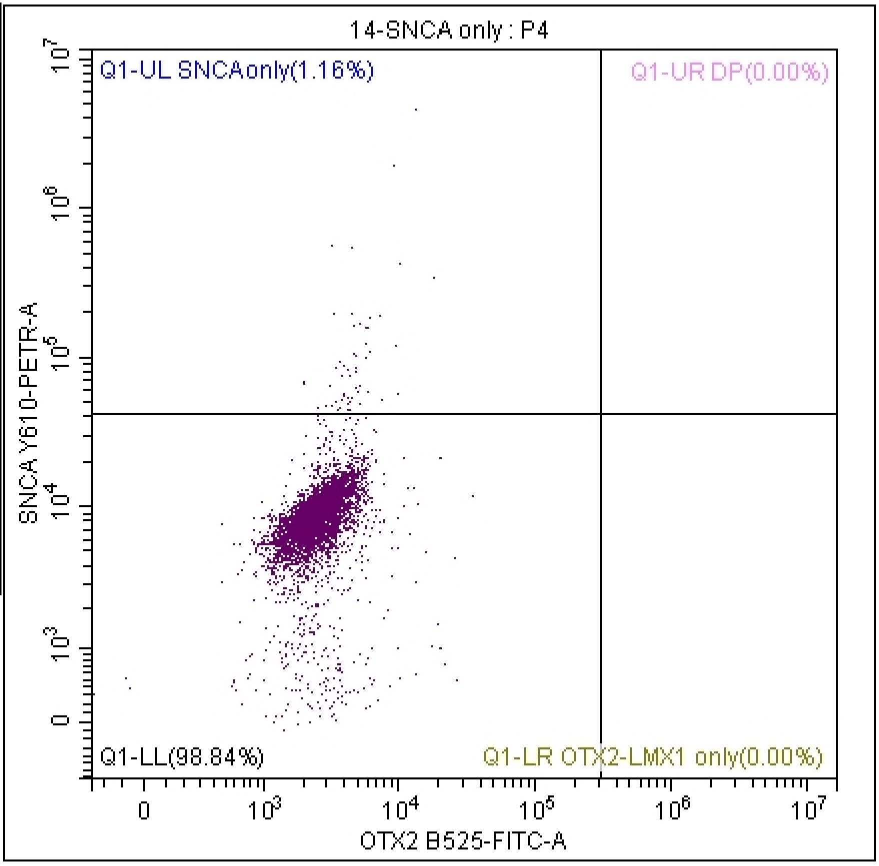

Tube Name: 14-SNCA only

Sample ID:

Population	Events	% Total	% Parent
All Events	25000	100.00%	100.00%
P1	5058	20.23%	20.23%
P2	4652	18.61%	91.97%
P4	4641	18.56%	99.76%
Q1-UR DP	0	0.00%	0.00%
Q1-UL SNCA only	54	0.22%	1.16%
Q1-LL	4587	18.35%	98.84%
Q1-LR OTX2-LMX1 only	0	0.00%	0.00%

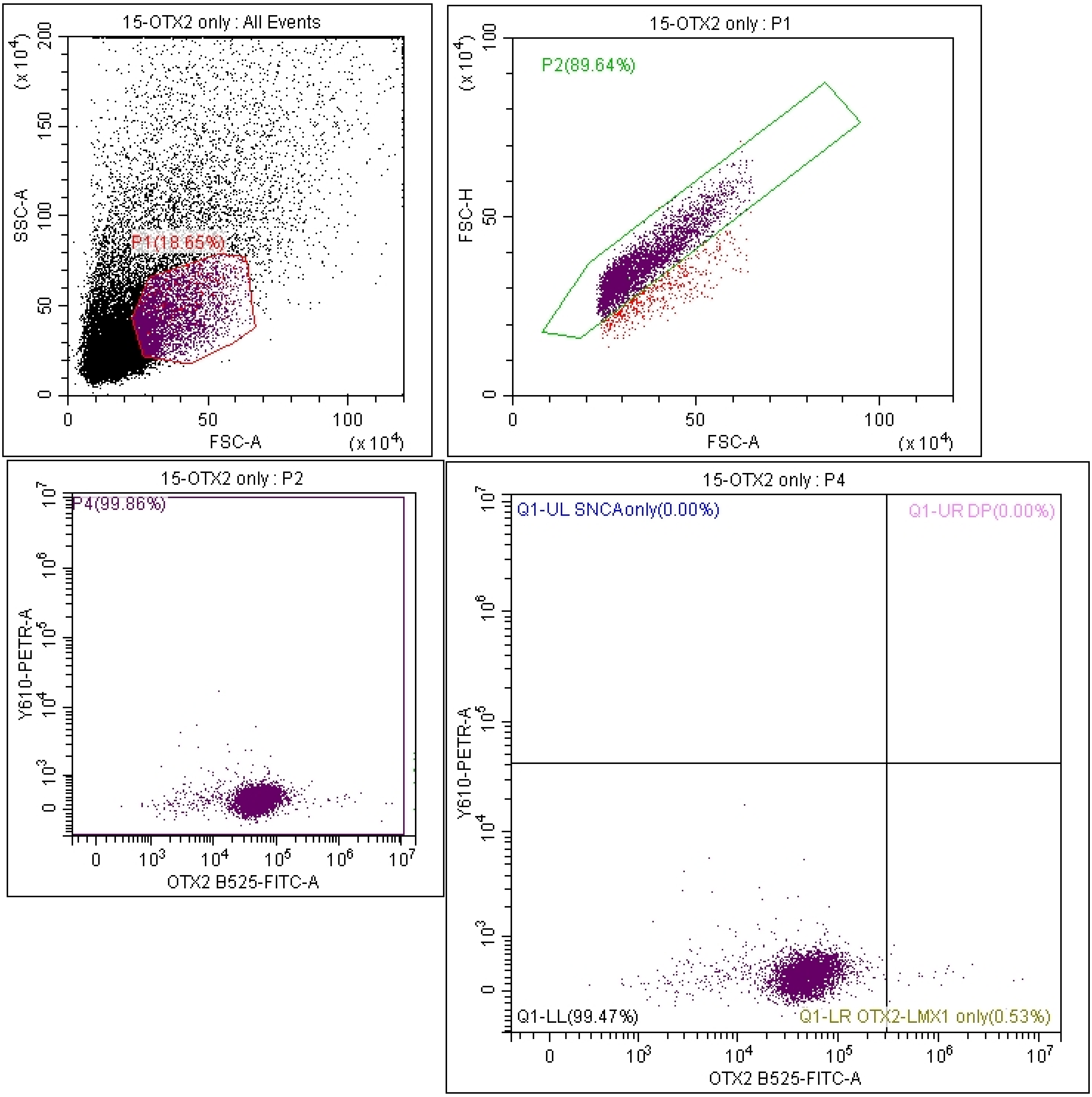

Tube Name: 15-OTX2 only

Sample ID:

Population	Events	% Total	% Parent
All Events	25000	100.00%	100.00%
P1	4662	18.65%	18.65%
P2	4179	16.72%	89.64%
P4	4173	16.69%	99.86%
Q1-UR DP	0	0.00%	0.00%
Q1-UL SNCA only	0	0.00%	0.00%
Q1-LL	4151	16.60%	99.47%
Q1-LR OTX2-LMX1 only	22	0.09%	0.53%

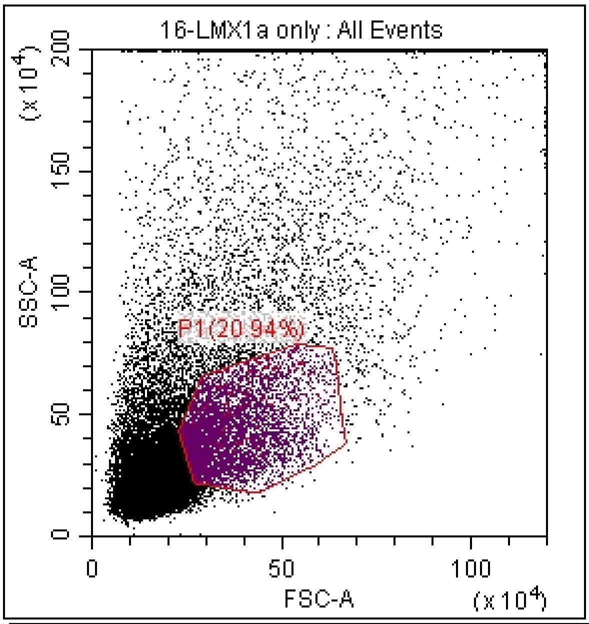

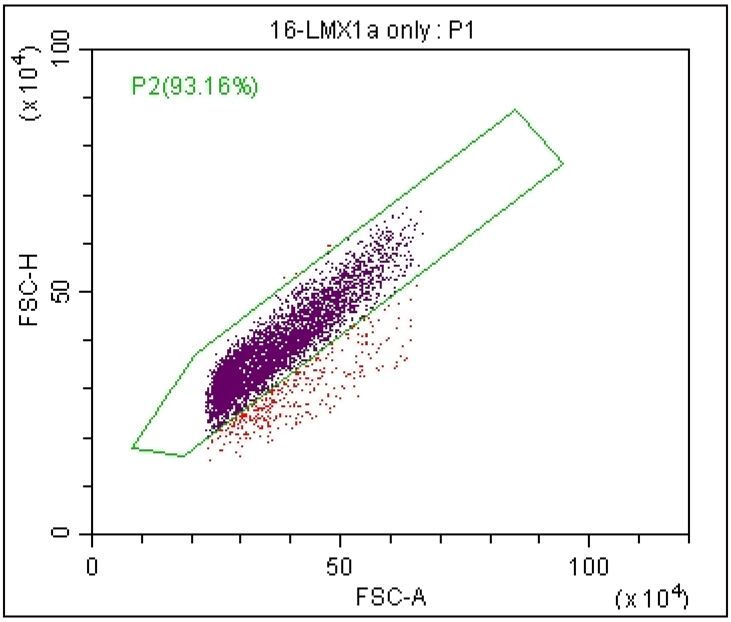

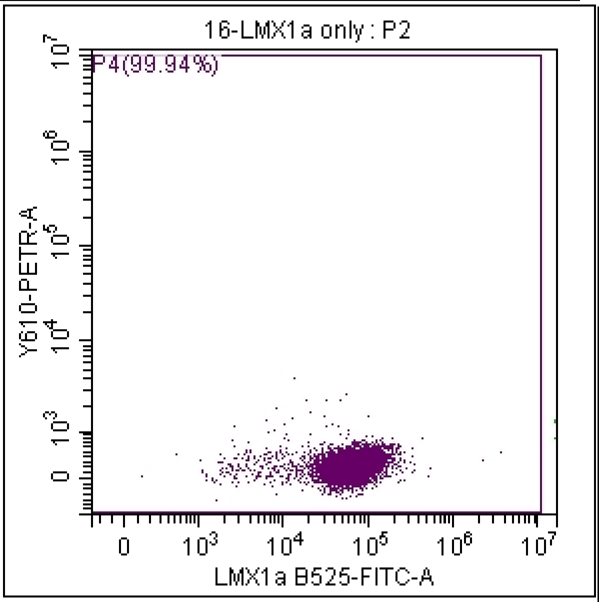

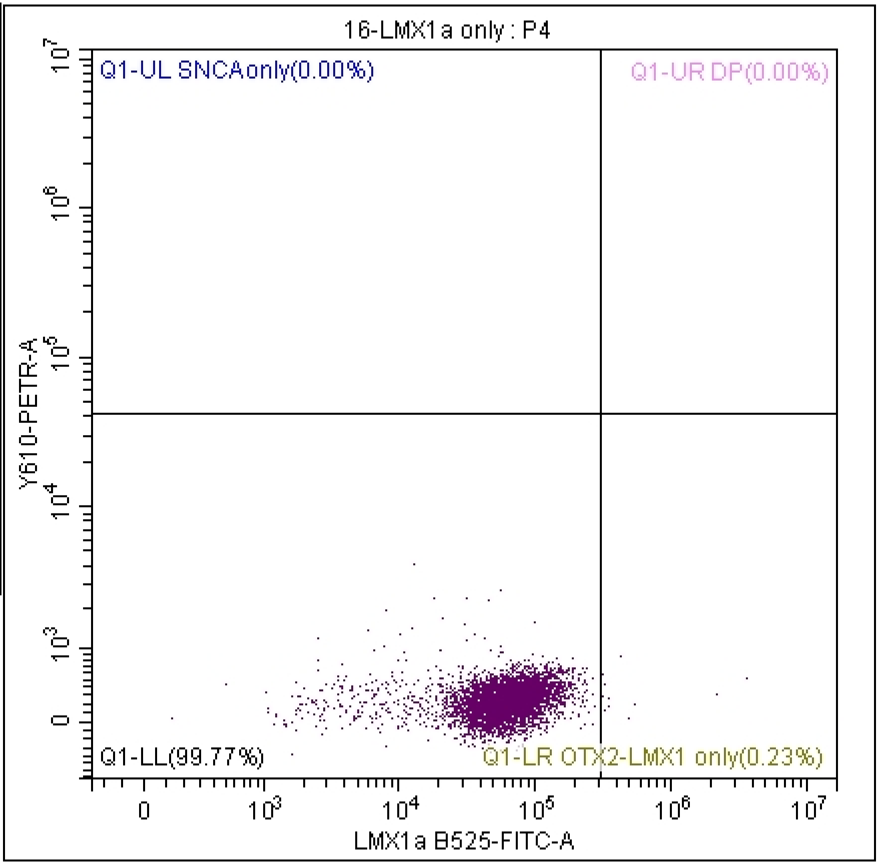

Tube Name: 16-LMX1a only

Sample ID:

Population	Events	% Total	% Parent
All Events	25000	100.00%	100.00%
P1	5234	20.94%	20.94%
P2	4876	19.50%	93.16%
P4	4873	19.49%	99.94%
Q1-UR DP	0	0.00%	0.00%
Q1-UL SNCA only	0	0.00%	0.00%
Q1-LL	4862	19.45%	99.77%
Q1-LR OTX2-LMX1 only	11	0.04%	0.23%

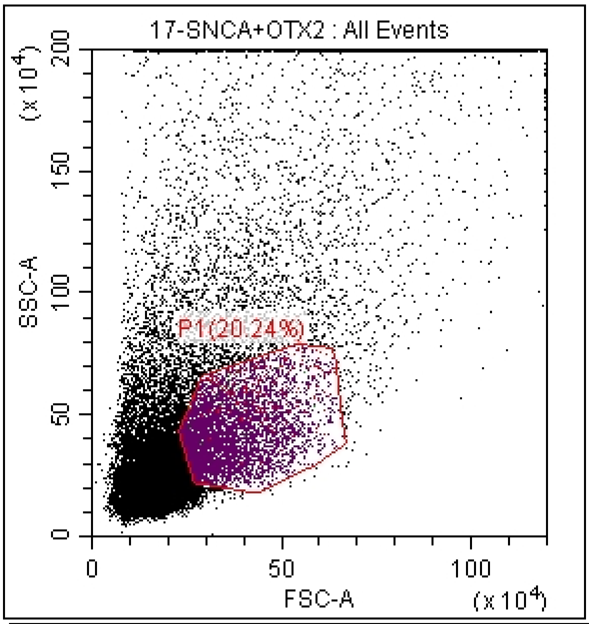

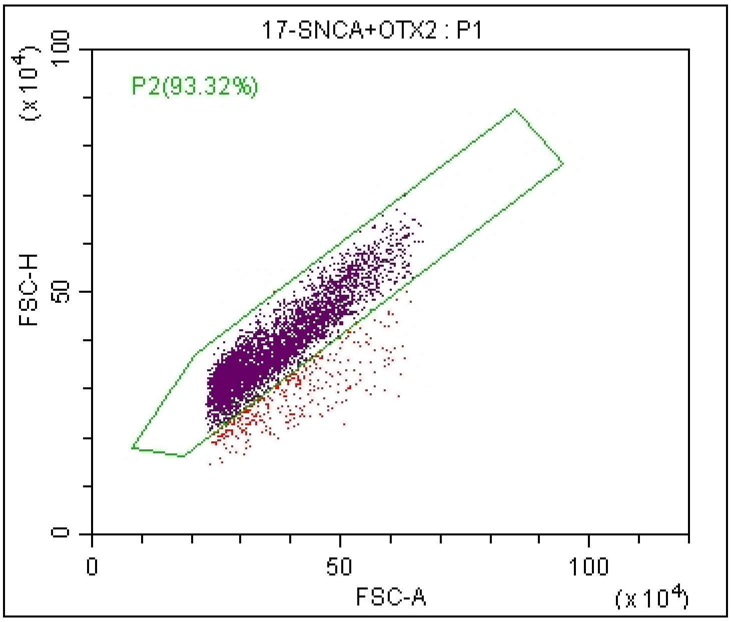

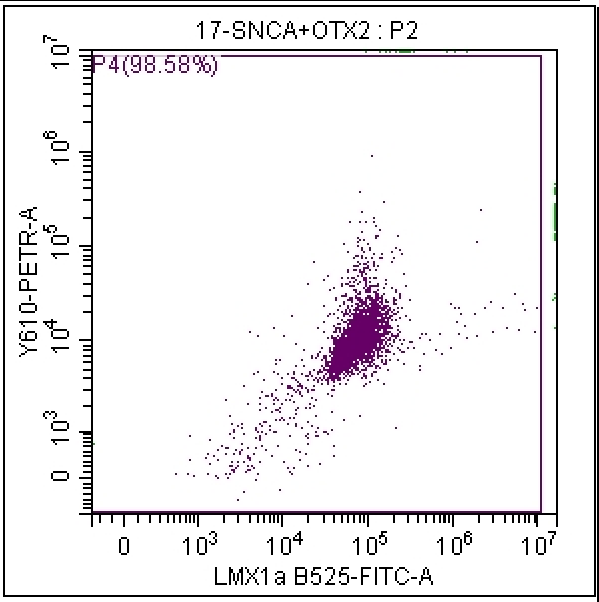

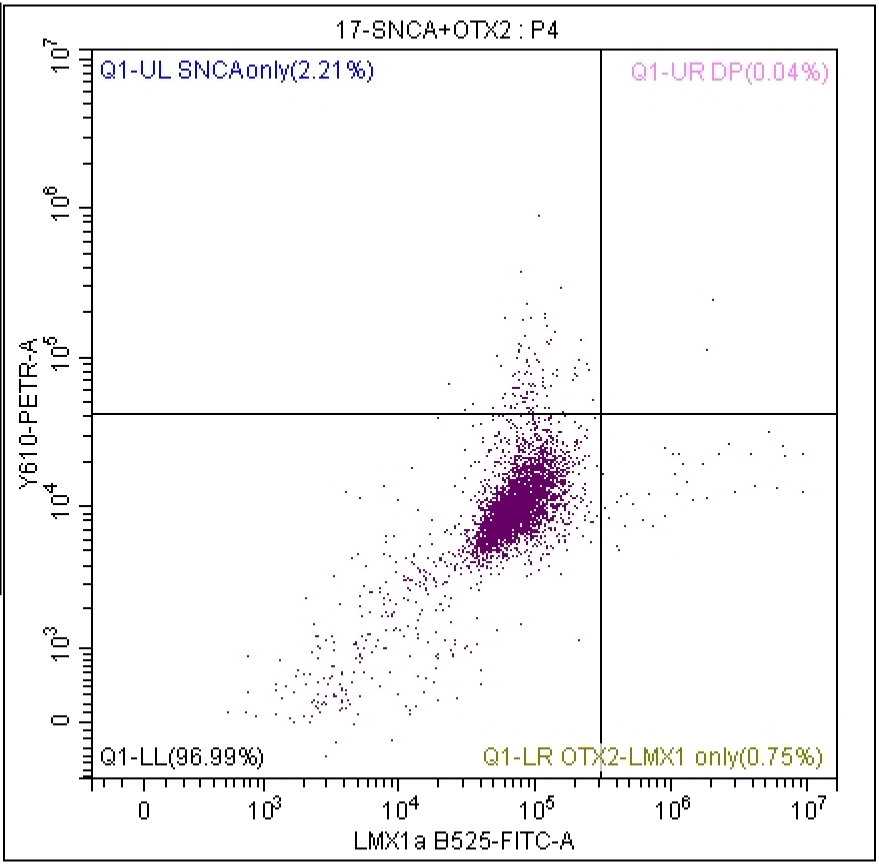

Tube Name: 17-SNCA+OTX2

Sample ID:

Population	Events	% Total	% Parent
All Events	25000	100.00%	100.00%
P1	5059	20.24%	20.24%
P2	4721	18.88%	93.32%
P4	4654	18.62%	98.58%
Q1-UR DP	2	0.01%	0.04%
Q1-UL SNCA only	103	0.41%	2.21%
Q1-LL	4514	18.06%	96.99%
Q1-LR OTX2-LMX1 only	35	0.14%	0.75%

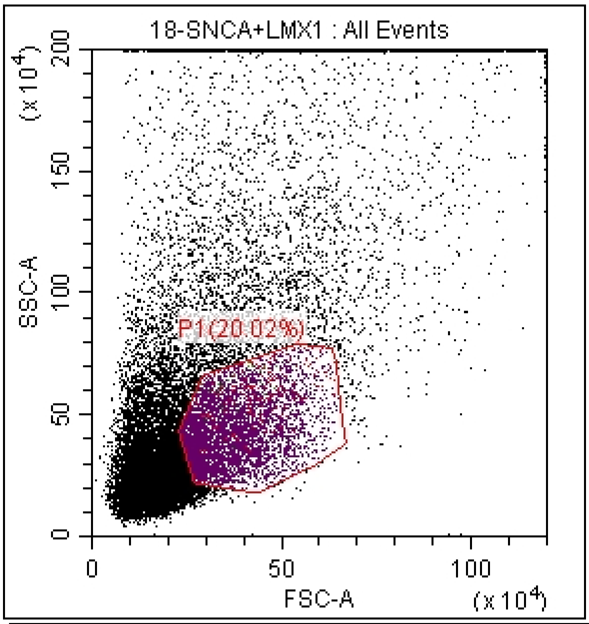

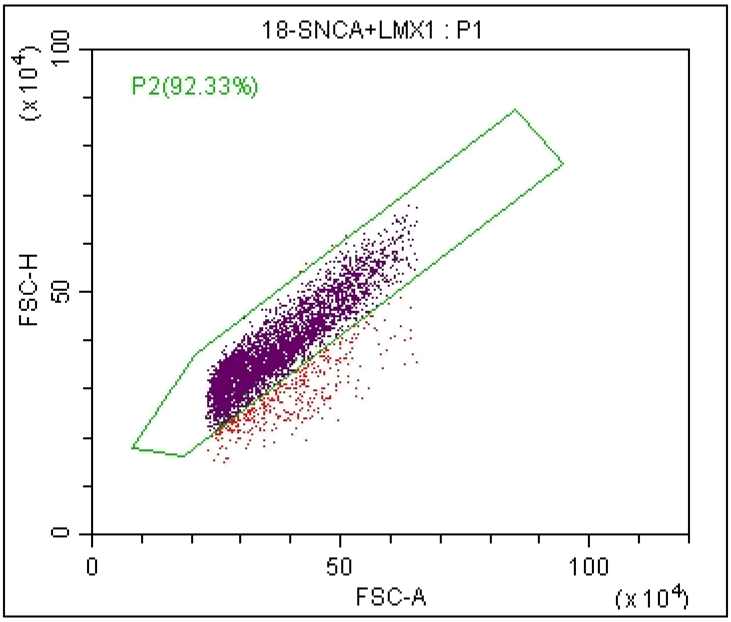

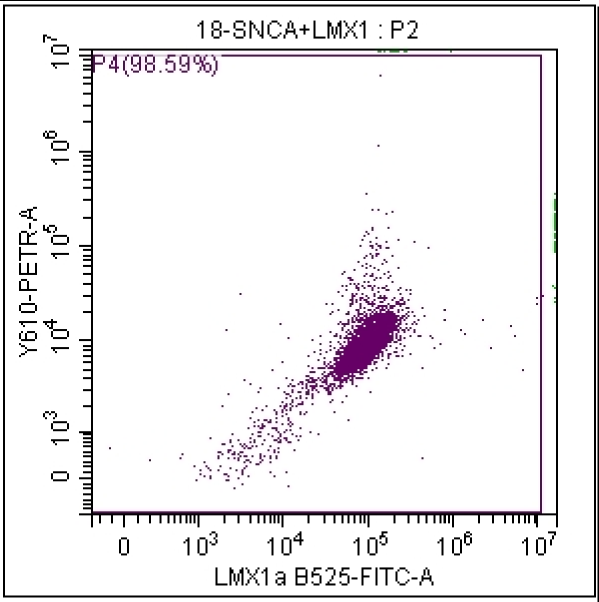

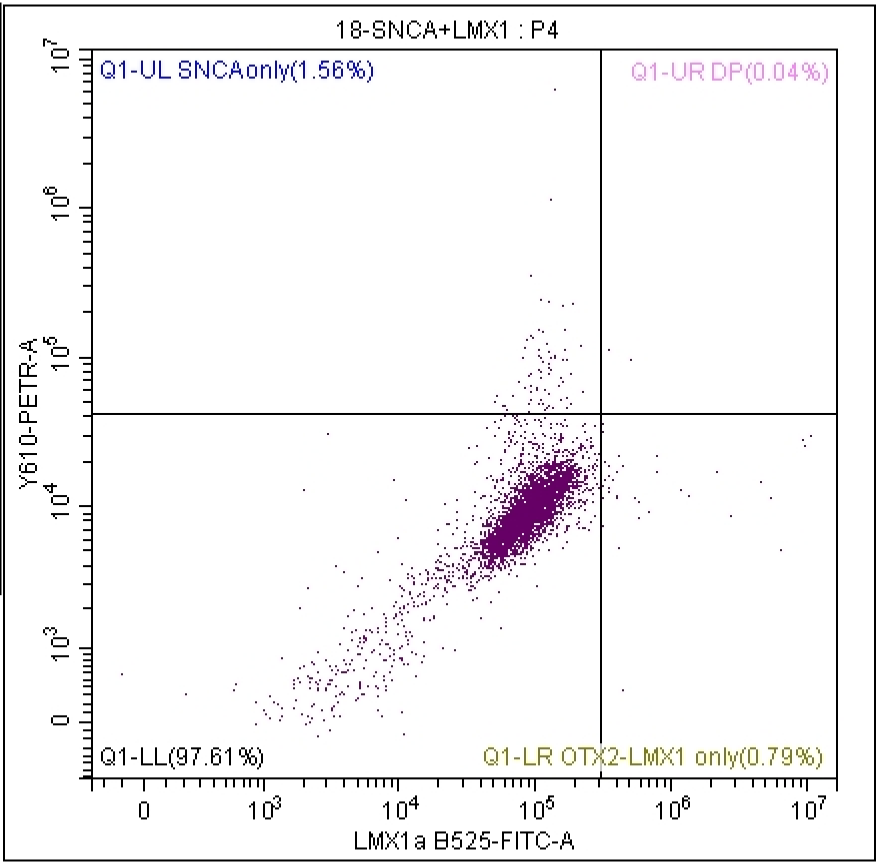

Tube Name: 18-SNCA+LMX1

Sample ID:

Population	Events	% Total	% Parent
All Events	25000	100.00%	100.00%
P1	5006	20.02%	20.02%
P2	4622	18.49%	92.33%
P4	4557	18.23%	98.59%
Q1-UR DP	2	0.01%	0.04%
Q1-UL SNCA only	71	0.28%	1.56%
Q1-LL	4448	17.79%	97.61%
Q1-LR OTX2-LMX1 only	36	0.14%	0.79%
